# Scheduled Intermittent Screening with Rapid Diagnostic Tests and Treatment with Dihydroartemisinin-Piperaquine versus Intermittent Preventive Therapy with Sulfadoxine-Pyrimethamine for Malaria in Pregnancy in Malawi: An Open-Label Randomized Controlled Trial

**DOI:** 10.1371/journal.pmed.1002124

**Published:** 2016-09-13

**Authors:** Mwayiwawo Madanitsa, Linda Kalilani, Victor Mwapasa, Anna M. van Eijk, Carole Khairallah, Doreen Ali, Cheryl Pace, James Smedley, Kyaw-Lay Thwai, Brandt Levitt, Duolao Wang, Arthur Kang’ombe, Brian Faragher, Steve M. Taylor, Steve Meshnick, Feiko O. ter Kuile

**Affiliations:** 1 College of Medicine, University of Malawi, Blantyre, Malawi; 2 Department of Clinical Sciences, Liverpool School of Tropical Medicine, Liverpool, United Kingdom; 3 National Malaria Control Programme, Ministry of Health, Lilongwe, Malawi; 4 Department of Epidemiology, Gillings School of Global Public Health, University of North Carolina at Chapel Hill, Chapel Hill, North Carolina, United States of America; 5 Department of Molecular Genetics and Microbiology, Duke University Medical Center, Durham, North Carolina, United States of America; 6 Division of Infectious Diseases, Department of Medicine, Duke University School of Medicine, Durham, North Carolina, United States of America; 7 Duke Global Health Institute, Duke University, Durham, North Carolina, United States of America; Mahidol-Oxford Tropical Medicine Research Unit, THAILAND

## Abstract

**Background:**

In Africa, most plasmodium infections during pregnancy remain asymptomatic, yet are associated with maternal anemia and low birthweight. WHO recommends intermittent preventive therapy in pregnancy with sulfadoxine-pyrimethamine (IPTp-SP). However, sulfadoxine-pyrimethamine (SP) efficacy is threatened by high-level parasite resistance. We conducted a trial to evaluate the efficacy and safety of scheduled intermittent screening with malaria rapid diagnostic tests (RDTs) and treatment of RDT-positive women with dihydroartemisinin-piperaquine (DP) as an alternative strategy to IPTp-SP.

**Methods and Findings:**

This was an open-label, two-arm individually randomized superiority trial among HIV-seronegative women at three sites in Malawi with high SP resistance. The intervention consisted of three or four scheduled visits in the second and third trimester, 4 to 6 wk apart. Women in the IPTp-SP arm received SP at each visit. Women in the intermittent screening and treatment in pregnancy with DP (ISTp-DP) arm were screened for malaria at every visit and treated with DP if RDT-positive. The primary outcomes were adverse live birth outcome (composite of small for gestational age, low birthweight [<2,500 g], or preterm birth [<37 wk]) in paucigravidae (first or second pregnancy) and maternal or placental plasmodium infection at delivery in multigravidae (third pregnancy or higher). Analysis was by intention to treat.

Between 21 July 2011 and 18 March 2013, 1,873 women were recruited (1,155 paucigravidae and 718 multigravidae). The prevalence of adverse live birth outcome was similar in the ISTp-DP (29.9%) and IPTp-SP (28.8%) arms (risk difference = 1.08% [95% CI −3.25% to 5.41%]; all women: relative risk [RR] = 1.04 [95% CI 0.90–1.20], *p* = 0.625; paucigravidae: RR = 1.10 [95% CI 0.92–1.31], *p* = 0.282; multigravidae: RR = 0.92 [95% CI 0.71–1.20], *p* = 0.543). The prevalence of malaria at delivery was higher in the ISTp-DP arm (48.7% versus 40.8%; risk difference = 7.85%, [95% CI 3.07%–12.63%]; all women: RR = 1.19 [95% CI 1.07–1.33], *p* = 0.007; paucigravidae: RR = 1.16 [95% CI 1.04–1.31], *p* = 0.011; multigravidae: RR = 1.29 [95% CI 1.02–1.63], *p* = 0.037). Fetal loss was more common with ISTp-DP (2.6% versus 1.3%; RR = 2.06 [95% CI 1.01–4.21], *p* = 0.046) and highest among non-DP-recipients (3.1%) in the ISTp-DP arm. Limitations included the open-label design.

**Conclusions:**

Scheduled screening for malaria parasites with the current generation of RDTs three to four times during pregnancy as part of focused antenatal care was not superior to IPTp-SP in this area with high malaria transmission and high SP resistance and was associated with higher fetal loss and more malaria at delivery.

**Trial Registration:**

Pan African Clinical Trials Registry PACTR201103000280319; ISRCTN Registry ISRCTN69800930

## Abstract 2

In an open-label randomized controlled trial, Feiko O. ter Kuile and colleagues found that ISTp-DP was not superior to IPTp-SP in an area with high malaria transmission and high SP resistance.

## Author Summary

**Why Was This Study Done?:**

- Malaria infection during the course of pregnancy can have devastating consequences on the mother and unborn child.
- Intermittent preventive treatment in pregnancy (IPTp) with the antimalarial sulfadoxine-pyrimethamine (SP) is one of the main interventions to protect pregnant women during pregnancy in malaria endemic areas in sub-Saharan Africa.
- The effectiveness of SP, however, is threatened by increasing resistance of the malaria parasite to this drug in east and southern Africa.
- We conducted this study to evaluate if an alternative strategy consisting of screening pregnant women for malaria with rapid diagnostic tests at regular intervals during pregnancy and then treating the test-positive women with dihydroartemisinin-piperaquine (DP) would reduce the risk of malaria infection and the adverse consequences to the mother and newborn. This strategy is called intermittent screening and treatment in pregnancy (ISTp).

**What Did the Researchers Do and Find?:**

- Our team conducted a two-arm, open-label trial to compare the effect of the new ISTp with DP (ISTp-DP) strategy against the existing IPTp with SP (IPTp-SP) strategy (the control arm) in 1,873 pregnant women in southern Malawi, where almost all of the malaria parasites were highly resistant to SP.
- We found that the rate of malaria infection was high in both groups and that the new ISTp-DP strategy was not any better than the existing IPTp-SP strategy in terms of reducing malaria infection or improving pregnancy outcomes; in fact, women in the ISTp-DP arm had more malaria than women in the IPTp-SP arm.

**What Do These Findings Mean?:**

- ISTp-DP with the current generation of rapid diagnostic tests is not a viable alternative strategy to replace IPTp-SP in malaria endemic areas in sub-Saharan Africa, despite the high levels of resistance to SP.
- IPTp with SP should still be used as one of the interventions against malaria in pregnancy in sub-Saharan Africa.
- Further studies to explore alternative drugs that can replace SP for IPTp will be required in these areas of high SP resistance.

## Introduction

Malaria during pregnancy is a major preventable cause of poor birth outcomes in sub-Saharan Africa [1]. In sub-Saharan Africa, the World Health Organization (WHO) currently recommends intermittent preventive treatment in pregnancy (IPTp) with sulfadoxine-pyrimethamine (SP) (IPTp-SP) for HIV-seronegative women. The effectiveness of IPTp-SP to clear peripheral parasitemia decreases in areas where parasites are resistant to SP; this resistance results from a series of mutations in the parasite genes that encode the targets of pyrimethamine (*dhfr*) and sulfadoxine (*dhps*). For example, in settings where >90% of parasites harbor high-level SP resistance encoded by five mutations in *dhfr* and *dhps*, up to 40% of asymptomatic parasitemic women who receive SP for IPTp are parasitemic again by day 42, reflecting the failure of SP to clear existing plasmodium infections and prevent new infections [2]. Nevertheless, even in these high resistance settings, SP retains some beneficial effect on birthweight [2,3]. However, an additional mutation at codon 581 in *dhps* is emerging in parasites in East Africa that renders IPTp-SP unable to inhibit parasite growth and may significantly compromise IPTp-SP when present [4–6]. Consequently, alternative approaches are required to prevent malaria during pregnancy.

Most of the proposed alternative drugs to replace SP are too poorly tolerated for IPTp use, including amodiaquine alone or combined with SP [7], mefloquine monotherapy [8,9], and the fixed-dose combination of chloroquine-azithromycin [10]. A proposed alternative strategy to IPTp consists of scheduled antenatal testing with rapid diagnostic tests (RDTs) and the treatment of RDT-positive women with artemisinin-based combination therapy (ACT), referred to as intermittent screening and treatment in pregnancy (ISTp) [11]. In West African settings, where parasite resistance to SP is low, ISTp with artemether-lumefantrine (AL) (ISTp-AL) was not inferior to IPTp-SP in reducing low birthweight and was well-accepted by providers and patients [12–14]. Nevertheless, in these studies, women in the ISTp-AL arm had lower mean birthweights and more clinical malaria during pregnancy.

We hypothesized that, owing to widespread parasite SP resistance, ISTp with the ACT dihydroartemisinin-piperaquine (DP) would be superior to IPTp-SP for the prevention of the adverse sequelae of malaria in pregnancy. However, a recent trial in an area with high levels of malaria transmission and parasite resistance to SP in western Kenya showed that ISTp with DP (ISTp-DP) was not superior to IPTp-SP and was associated with increased incidence of clinical malaria and malaria infection [15]. These findings need to be confirmed urgently in other areas that have high levels of parasite resistance to SP. Here we report the results of a similar trial comparing IPTp-SP against ISTp-DP in Malawi.

## Methods

### Ethics Statement

Ethical approval was obtained from the Liverpool School of Tropical Medicine (LSTM) and the Malawian National Health Science Research Committee. Written informed consent was obtained from all participants prior to randomization.

### Study Design and Participants

This was a three-site, open-label, two-arm individually randomized superiority trial using a stratified design with one strata for primi- and secundigravidae (paucigravidae) and one for multigravidae (third pregnancy or higher). The study was conducted at the Mpemba and Madziabango Health Centers and the Chikwawa District Hospital in southern Malawi. The area has moderate to intense year-round malaria transmission and high levels of SP resistance, as evidenced by near fixation of parasites harboring mutations at codons 51, 59, and 108 of *dhfr* and 437 and 540 of *dhps* [16,17].

Women of all gravidae attending their first antenatal visit were eligible if they were HIV-seronegative, were resident in the study catchment area and willing to deliver at the study clinics/hospital, had a hemoglobin > 70 g/l, had a pregnancy between 16 and 28 wk gestation, and had not yet received IPTp-SP. Exclusion criteria included multiple gestation and other high-risk pregnancies according to national guidelines, previous enrollment in the same study, and history of allergy to any of the study drugs.

### Randomization and Masking

Randomization sequences were computer-generated by the study statistician at LSTM, one for each gravidity strata and study site, using variable block randomization and an allocation ratio of 1:1. In each clinic, eligible women were allocated to the IPTp-SP or ISTp-DP arm by the coordinating study staff in order of their study identification number by drawing sequentially numbered opaque envelopes containing the allocation arm from a box corresponding to each gravidity stratum. Following allocation, women and care providers were aware of the arm allocation. All laboratory staff were blinded to the treatment assignment. The study statistician remained blinded until after database lock and approval of the statistical analysis plan by the data and safety monitoring board.

### Procedures

At enrollment, demographic, socioeconomic, and educational information was collected, a medical and obstetric history taken, and the gestational age ascertained by ultrasound. A 5-ml venous blood sample was taken for malaria microscopy, PCR, immunology, and testing for syphilis, HIV serostatus, and hemoglobin concentration (Hemocue). All women received a long-lasting insecticide-treated net.

Participants were randomized to receive either IPTp-SP or ISTp-DP, at enrollment and all subsequent scheduled antenatal visits. The IPTp-SP arm received three tablets of SP (500 mg/25 mg sulfadoxine/pyrimethamine tablets). If they had fever or history of fever, they were tested for malaria by RDT. RDT-positive women were treated with AL and then received their first course of SP during the first scheduled follow-up visit. Women in the ISTp-DP arm were screened for malaria using the histidine-rich protein 2 (HRP2)/plasmodium lactate dehydrogenase (pLDH) combination RDT (First Response Malaria pLDH/HRP2 Combo Test, Premier Medical Corporation). All RDT-positive women in the ISTp-DP arm received a standard 3-d course of DP (Eurartesim, Sigma Tau; 40 mg/320 mg dihydroartemisinin/piperaquine tablets) at a dose of 2.5, 3, 3.5, and 4 tablets for women weighing <50, 50–59, 60–69, and ≥70 kg, respectively. All SP and DP doses were provided with a slice of dry bread as directly observed therapy. All doses in both arms were supervised. In case of vomiting within 30–60 min, the full dose was repeated. If the repeat dose was vomited, the women received AL. Sigma Tau provided the Eurartesim free of charge.

The follow-up schedule consisted of three or four scheduled antenatal visits every 4 to 6 wk: four if enrolled at 16–24 wk gestation or three if enrolled at ≥25 wk gestation. At each such visit, a clinical and obstetric examination was conducted, and a blood sample taken for RDT (ISTp-DP arm), malaria microscopy, and PCR. Hemoglobin was assessed during the last scheduled visit. Women were encouraged to make unscheduled visits if they felt ill or were concerned about their pregnancy. In the IPTp-SP arm, women with uncomplicated clinical malaria (fever/history of fever and RDT-positive) during or in between scheduled visits received AL. Women with uncomplicated malaria in the ISTp-DP arm received DP, or AL if they had received DP within the previous 4 wk.

At delivery, a maternal venous sample was taken for the same malaria metrics, and a placental and cord-blood sample for histology, RDT, microscopy, and PCR. Children were weighed and the gestational age assessed using the modified Ballard score [18]. The presence of congenital abnormalities and jaundice was assessed at delivery, at day 7, and at the final visit at 6–8 wk, coinciding with their childhood vaccination visit. In between scheduled visits, infants were followed passively.

RDT results were used to determine care. RDT positivity was defined as either pLDH or HRP2 antigen positivity. See S1 Text for details of microscopy and real-time PCR used for detection and identification of parasites, as well as baseline parasite genotyping.

### Outcomes

The primary outcome among paucigravidae was “adverse live birth outcome,” defined as the composite of having a singleton baby born small for gestational age (SGA) [19] or with low birthweight (<2,500 g), or preterm (<37 wk) (S1 Text). The primary outcome among multigravidae was a composite of any evidence for plasmodium infection at delivery detected in peripheral maternal blood (microscopy, RDT, or PCR) or placenta (incision smear, impression smear, PCR, or active or past infection detected by histology) (S1 Text). The rationale for using a different primary outcome for multigravidae was based on systematic reviews showing that preventing plasmodium infection by IPTp-SP or long-lasting insecticide-treated nets is associated with improved birth outcomes primarily among women in their first and second pregnancies [20,21]. Plasmodium infection status at delivery was used as the primary outcome in multigravidae because plasmodium infection is associated with an increased risk of malaria [22–25] and anemia [26–28] in infancy, particularly in those born to multigravidae [24].

Key secondary efficacy outcomes included the individual components of the composite primary outcomes, fetal loss (spontaneous abortion at <28 wk gestation, stillbirth), any adverse birth outcome (adverse live birth outcome or fetal loss), maternal hemoglobin concentrations and anemia, clinical malaria (documented fever/history of fever plus positive malaria RDT), plasmodium infection, mean birthweight, mean gestational age at delivery, congenital plasmodium infection (cord blood positive at birth by microscopy, RDT, or PCR, or clinical malaria within 7 d of birth with parasitological confirmed diagnosis by microscopy or RDT), neonatal and infant (by 6–8 wk) clinical malaria, all-cause severe anemia and all-cause illness detected at scheduled or unscheduled postnatal visits, and perinatal and infant mortality by 6–8 wk.

The primary safety outcomes included maternal death, severe cutaneous skin reaction in the mothers within 30 d of drug intake, other serious adverse events (SAEs) in the mother or infant, congenital malformations, and neonatal jaundice.

### Statistical Analysis

See S1 Text for details about sample size calculations. Log binomial regression was used for binary endpoints to obtain relative risk (RR) values and corresponding 95% confidence intervals. The identity-link function was used to obtain risk differences. Linear regression was used for continuous variables, and results expressed as mean difference (95% CI). The unadjusted analysis, stratified by gravidity (pauci- and multigravidae), was considered the primary analysis. Secondary, covariate-adjusted analyses for the primary endpoints were conducted using seven prespecified covariates (in addition to gravidity and site) and simple imputation for missing covariates (<1%). These same covariates were included in subgroup analyses. Poisson regression with time of follow-up as an offset was used for count variables to obtain incidence rate ratios (95% CIs). A two-sided *p*-value < 0.05 was used to define statistical significance. The intention to treat (ITT) analytical population was defined as all eligible women who were randomized and contributed to the outcome. The per protocol population included women who attended every scheduled visit, who took all the daily study doses on each occasion, and who contributed to the endpoint. For the safety analysis, women in the ISTp-DP arm were considered overall and split by recipients and non-recipients of DP (i.e., those who were RDT-negative throughout). All analyses were prespecified, unless otherwise indicated, in a statistical analysis plan (see S2 Text) approved by the data and safety monitoring board. Analysis was done in SAS version 9.3 and Stata version 14.

## Results

### Baseline and Patient Disposition

**Fig 1:**
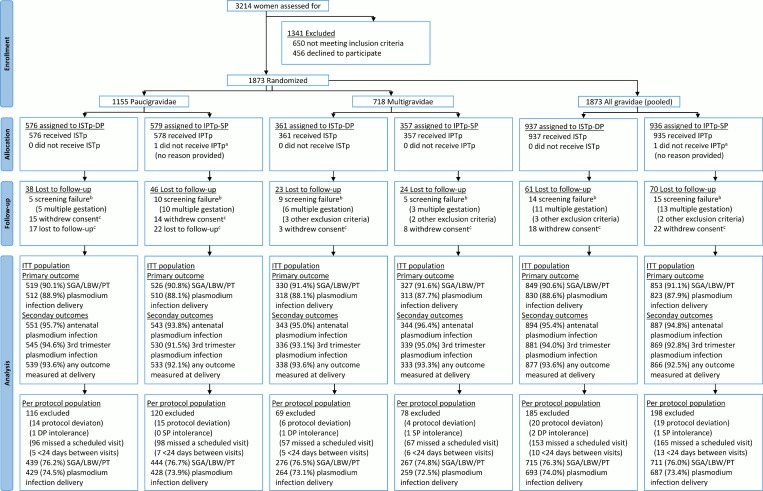
Flow chart. ^a^One woman randomized to IPTp-SP was erroneously recorded as being in the ISTp-DP arm on her antenatal care card and as a result received ISTp-DP. She was included in the ITT population under the IPTp-SP arm. ^b^Screening failures were not followed to delivery and were excluded from the modified ITT population. ^c^Women lost to follow-up prior to delivery and women who withdrew consent were included in the ITT population and contributed to the antenatal follow-up analyses (e.g., incidence of malaria). IPTp-SP, intermittent preventive therapy in pregnancy with sulfadoxine-pyrimethamine; ISTp-DP, intermittent screening and treatment in pregnancy with dihydroartemisinin-piperaquine; ITT, intention to treat; SGA/LBW/PT, small for gestational age or low birthweight or preterm.

Between 21 July 2011 and 18 March 2013, 3,214 women were screened for inclusion; 1,873 women were randomized (paucigravidae, *n* = 1,155; multigravidae, *n* = 718). Recruitment was stopped when the full sample size for paucigravidae had been reached (S1 Text). Of the randomized women, 1,743 (93.1%) were seen at delivery (Fig 1). Overall, 6,504 of 6,942 (93.7%) scheduled antenatal follow-up visits were attended (S1 Table), and 1,742 women (94.5%) attended all scheduled visits. Ultimately, 1,676 (89.5%) contributed to the primary endpoint, with proportions of participants equally distributed between the study arms (ISTp-DP arm, 89.3%; IPTp-SP arm, 89.6%) (S2 Table). The baseline characteristics were well balanced between the study arms, within each gravidity strata, and overall (Table 1). At baseline, about half of the women were infected with malaria parasites, and this proportion was slightly lower (not significant) in those not contributing to the primary analyses (S3 Table). Overall, 99.5% and 2.7% of the parasites harbored the *dhps* K540E and A581G mutation, respectively (S4 Table). In both arms, the median (interquartile range) follow-up time was 4.0 (3.2–4.7) mo, and the median (range) number of scheduled visits was 4 (1–4) (S1 Table). In the ISTp-DP arm, 48.8%, 38.0%, 12.4%, and 0.9% received 0, 1, 2, and 3 courses of DP, respectively (S1 Table). Overall, 3,048 and 604 courses of SP and DP were administered in the respective study arms, and in the IPTp-SP arm, 251 courses of AL were administered for clinical malaria (S1 Table).

**Table 1:** Baseline characteristics (intention to treat population).

Characteristic	Paucigravidae		Multigravidae		All Gravidae (Pooled)	
	ISTp-DP (*n =* 571)	IPTp-SP (*n =* 569)	ISTp-DP (*n =* 352)	IPTp-SP (*n =* 352)	ISTp-DP (*n =* 923)	IPTp-SP (*n =* 921)
**Maternal characteristics**						
**Study site**						
Madziabango	21.9% (125/571)	22.1% (126/569)	31.3% (110/352)	30.4% (107/352)	25.5% (235/923)	25.3% (233/921)
Mpemba	51.5% (294/571)	51.5% (293/569)	54.3% (191/352)	55.4% (195/352)	52.6% (485/923)	53.0% (488/921)
Chikwawa	26.6% (152/571)	26.4% (150/569)	14.5% (51/352)	14.2% (50/352)	22.0% (203/923)	21.7% (200/921)
**Maternal age (years)**	19.5 (2.7)	19.6 (2.8)	27.3 (4.3)	27.5 (4.2)	22.5 (5.1)	22.6 (5.1)
**Marital status**						
Single	8.1% (46/570)	8.8% (50/569)	2.0% (7/351)	0.3% (1/352)	5.8% (53/921)	5.5% (51/921)
Married	91.9% (524/570)	91.2% (519/569)	97.7% (343/351)	99.1.% (349/352)	94.1% (867/921)	94.2% (868/921)
Widowed/separated/divorced	0.0% (0/570)	0.0% (0/569)	0.3% (1/351)	0.6% (2/352)	0.1% (1/921)	0.2% (2/921)
**Used a bednet last night**	18.2% (104/571)	17.0% (97/569)	18.2% (64/351)	24.1% (85/352)	18.2% (168/922)	19.8% (182/921)
**Schooling (years completed)**	6.7 (3.4)	6.7 (3.3)	4.4 (3.4)	4.4 (3.9)	5.8 (3.6)	5.9 (3.7)
**SES index score (terciles)**						
Low	34.7% (198/570)	33.3% (189/567)	33.3% (117/351)	31.5% (111/352)	34.2% (315/921)	32.6% (300/919)
Medium	31.2% (178/570)	32.1% (182/567)	37.0% (130/351)	36.1% (127/352)	33.4% (308/921)	33.6% (309/919)
High	34.0% (194/570)	34.6% (196/567)	29.6% (104/351)	32.4% (114/352)	32.4% (298/921)	33.7% (310/919)
**Rainfall in the 3 mo before enrollment (average/month, in millimeters)**	22.8 (3.0–117.5)	22.8 (3.0–117.5)	23.8 (3.3–117.5)	24.7 (3.3–117.5)	22.8 (3.3–117.5)	22.8 (3.3–117.5)
**Pregnancy number (gravidity)**						
First	54.5% (311/571)	55.5% (316/569)	NA	NA	33.8% (311/921)	34.3% (316/920)
Second	45.5% (260/571)	44.5% (253/569)	NA	NA	28.2% (260/921)	27.5% (253/920)
Third	NA	NA	44.0% (154/352)	43.0% (151/351)	16.7% (154/921)	16.4% (151/920)
Fourth or higher	NA	NA	56.0% (196/352)	57.0% (200/351)	21.3% (196/921)	21.7% (200/920)
**Gestational age by ultrasound (days)**	145.1 (23.0)	144.5 (22.4)	149.7 (23.7)	149.1 (23.8)	146.8 (23.3)	146.2 (23.0)
**Had a previous stillbirth/abortion**	5.1% (29/571)	4.0% (23/569)	14.2% (50/352)	10.8% (38/352)	8.6% (79/923)	6.6% (61/921)
**Maternal weight (kilograms)**	54.1 (6.8)	54.4 (6.9)	56.1 (7.5)	57.0 (8.5)	54.8 (7.2)	55.4 (7.6)
**Maternal height (centimeters)**	153.6 (4.8)	154.0 (5.0)	154.2 (5.2)	154.5 (5.0)	153.8 (5.0)	154.2 (5.0)
**Laboratory findings**						
**Hemoglobin (g/dl)**	10.7 (1.5)	10.7 (1.5)	11.5 (1.3)	11.5 (1.3)	11.0 (1.5)	11.0 (1.4)
**Plasmodium infection**						
RDT	43.8% (250/571)	NA	19.4% (68/351)	NA	34.5% (318/922)	NA
Microscopy	17.7% (100/565)	20.8% (117/562)	11.8% (41/347)	9.4% (33/351)	15.5% (141/912)	16.4% (150/913)
PCR	51.3% (285/556)	51.7% (290/561)	33.1% (115/347)	28.7% (98/342)	44.3% (400/903)	43.0% (388/903)
Microscopy or PCR	52.5% (300/571)	54.9% (312/568)	36.4% (128/352)	31.5% (111/352)	46.4% (428/923)	46.0% (423/920)
**Parasite density** ^**a**^	1,000 (320–7,200)	2,427 (507–9,600)	320 (160–3,200)	907 (213–4,267)	800 (267–5,867)	2,400 (320–8,000)

Data are percent (*n/N*), mean (standard deviation), or median (interquartile range).

^a^Parasite density per microliter, assessed by microscopy.

IPTp-SP, intermittent preventive therapy in pregnancy with sulfadoxine-pyrimethamine; ISTp-DP, intermittent screening and treatment in pregnancy with dihydroartemisinin-piperaquine; NA, not applicable; RDT, rapid diagnostic test; SES, socioeconomic status.

### Primary Outcome

**Fig 2:**
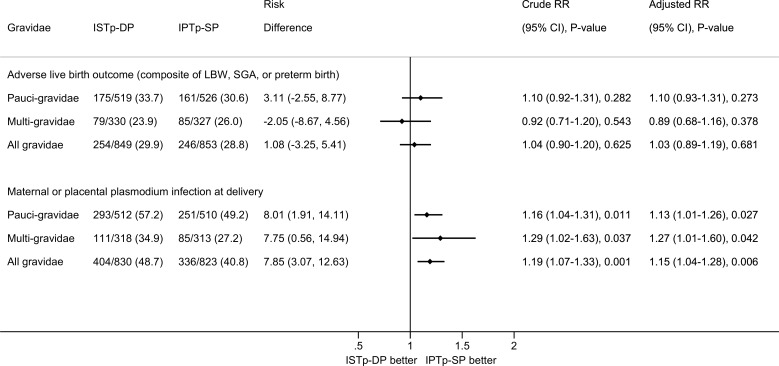
Efficacy of ISTp-DP versus IPTp-SP on the primary outcomes of adverse live birth outcome and maternal or placental plasmodium infection at delivery (any measure). Adjusted RR values obtained from multivariate log binomial regression models with missing values imputed and adjusting for gravidity, study site, and seven other prespecified covariates: malaria status at enrollment (binary), season during pregnancy (terciles based on average ranked rainfall during the last 6 mo of pregnancy), maternal height (terciles), hemoglobin status at enrollment (terciles), maternal years of schooling (terciles), socioeconomic status (terciles of socioeconomic index calculated using principal component analysis), and gestational age at first antenatal visit (binary based on median). There were no differences in effect size for paucigravidae versus multigravidae (*p*-value for interaction term: *p* = 0.271 for adverse live birth outcome and *p* = 0.454 for plasmodium infection at delivery). IPTp-SP, intermittent preventive therapy in pregnancy with sulfadoxine-pyrimethamine; ISTp-DP, intermittent screening and treatment in pregnancy with dihydroartemisinin-piperaquine; LBW, low birthweight; RR, relative risk; SGA, small for gestational age.

Among paucigravidae, the prevalence of adverse live birth outcome was similar in the ISTp-DP (33.7%) and IPTp-SP (30.6%) arms (RR = 1.10 [95% CI 0.92–1.31], *p* = 0.282; Fig 2). The prevalence was also similar between arms among multigravidae.

Among multigravidae, the risk of malaria at delivery was higher in the ISTp-DP (34.9%) than in the IPTp-SP (27.2%) arm (RR = 1.29 [95% CI 1.02–1.63], *p* = 0.037). This increased risk was also evident among paucigravidae and all gravidae. In absolute terms, the risk of malaria was increased in multigravidae by 7.8% (95% CI 0.6%–14.9%) and amongst all gravidae by 7.9% (95% CI 3.1%–12.6%) (Fig 2).

Similar results for both primary outcomes were obtained from prespecified covariate-adjusted analyses, with and without prespecified imputation for missing covariates (S5 Table), with per protocol population analysis (S6 Table), and in a sensitivity analysis that restricted analysis to birthweight obtained within 24 h of delivery (S7 Table). Results were also consistent across subgroups (S1 and S2 Figs), although the increased risk of malaria at delivery appeared lowest in primigravidae (S2 Fig).

### Secondary Efficacy Outcomes

**Fig 3:**
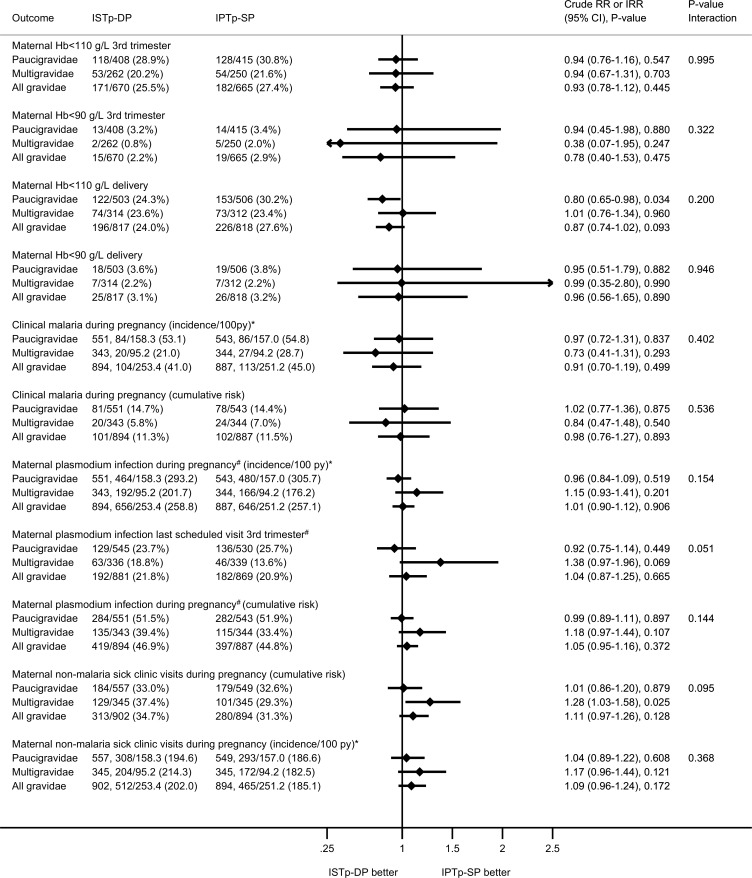
Secondary maternal outcomes: anemia and malaria. The *p*-value for the interaction term depicts the *p*-value for differences in effect size between paucigravidae and multigravidae. *Data given as the number of women with an event, the number of events/person-years of follow-up, and, in parentheses, the incidence rate per 100 person-years. ^#^Maternal plasmodium infection detected by PCR, microscopy, or RDT. RDT data considered only when women were symptomatic (febrile). To allow for comparison between study arms, the routine scheduled RDT data in the ISTp-DP arm were not included. Hb, hemoglobin; IPTp-SP, intermittent preventive therapy in pregnancy with sulfadoxine-pyrimethamine; IRR, incidence rate ratio; ISTp-DP, intermittent screening and treatment in pregnancy with dihydroartemisinin-piperaquine; py, person-years; RR, relative risk.

**Fig 4:**
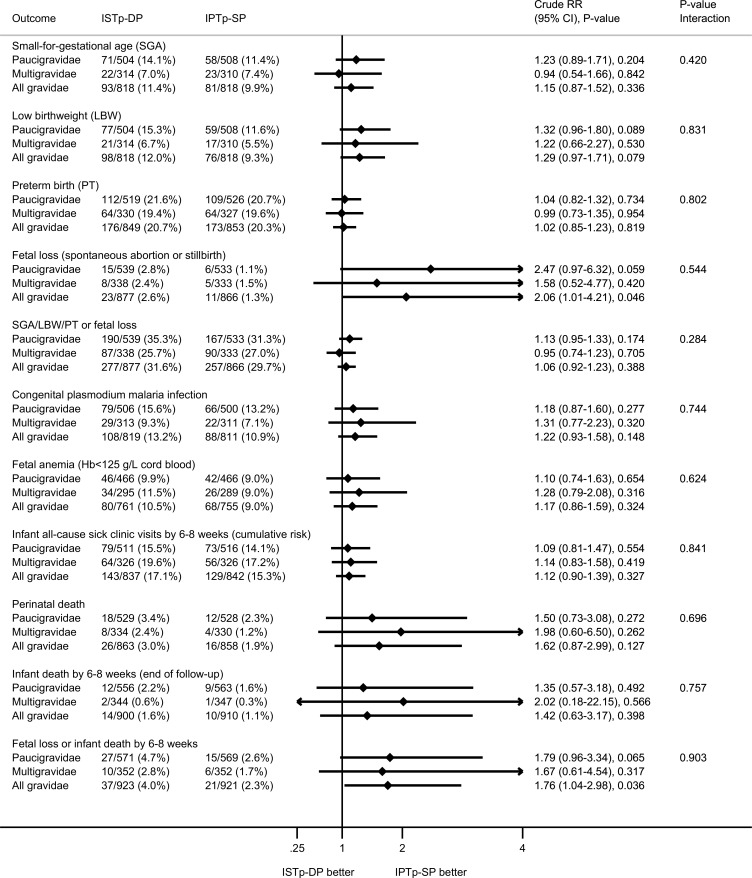
Secondary newborn outcomes: birth outcomes and neonatal follow-up. The *P*-value for the interaction term depicts the *p*-value for differences in effect size between paucigravidae and multigravidae. IPTp-SP, intermittent preventive therapy in pregnancy with sulfadoxine-pyrimethamine; ISTp-DP, intermittent screening and treatment in pregnancy with dihydroartemisinin-piperaquine; RR, relative risk.

Following enrollment, 45.8% of women had ≥1 episode of plasmodium infection prior to delivery (PCR, microscopy, or RDT), and 11.4% had ≥1 episode of clinical malaria. These proportions were similar in both arms (Fig 3). At delivery, 22.2% of women had peripheral malaria detected by PCR, RDT, or microscopy. This value was higher in the ISTp-DP arm (RR = 1.34 [95% CI 1.12–1.61], *p* = 0.002; S3 Fig), particularly for subpatent infections (PCR-positive, RDT- or microscopy-negative; S3 Fig). The overall prevalence of placental malaria detected by histology, PCR, RDT, or microscopy was 38.0%, and this value was higher in the ISTp-DP arm (RR = 1.16 [95% CI 1.03–1.32], *p* = 0.018; S4 Fig), reflecting differences in acute rather than chronic or past histological infections (S4 Fig). Congenital malaria was common (12.0%) in both groups (Fig 4).

At delivery, relative to the IPTp-SP arm, paucigravidae in the ISTp-DP arm had higher mean hemoglobin concentrations (S8 Table) and a lower prevalence of anemia (hemoglobin < 110 g/l) (Fig 3). The individual components of the primary endpoint adverse live birth outcome are provided in Fig 4. Low birthweight was more common in the ISTp-DP arm (RR = 1.29 [95% CI 0.97–1.71], *p* = 0.079).

### Adherence, Tolerance, Fetal Loss, Mortality, and Other Safety Outcomes

Overall, DP was well tolerated (S9 Table). There was no difference between arms in the number of maternal SAEs or deaths (S10 Table). There were no severe cutaneous reactions. Fetal loss was highest in the ISTp-DP arm (2.6% versus 1.3%; Fig 4). Further stratified analysis within the ISTp-DP arm showed fetal loss was highest among women who had never received DP (i.e., who remained RDT-negative throughout) (3.1% versus 2.2% in DP recipients; S10 Table). Perinatal and infant (by 6–8 wk) mortality were not statistically different between the arms (perinatal mortality: RR = 1.62 [95% CI 0.87–2.99], *p* = 0.127; infant mortality: RR = 1.42 [95% CI 0.63–3.17], *p* = 0.398) (Fig 4), but overall, the composite of fetal loss or infant death by the end of follow-up (6–8 wk) occurred more often in the ISTp-DP arm (4.0% versus 2.3%, RR = 1.76 [95% CI 1.04–2.98], *p* = 0.036; Fig 4; S10 Table). One case of neonatal jaundice was detected in the ISTp-DP arm (mother was a non DP-recipient), and none in the IPTp-SP arm. The frequency of congenital malformations was 1.2% in the ISTp-DP arm (0.9% in infants whose mother was a DP-recipient) and 1.0% in the IPTp-SP arm (RR = 1.11 [95% CI 0.45–2.71], *p* = 0.824).

## Discussion

Despite the high levels of parasite resistance to SP, ISTp-DP was not superior to the standard IPTp-SP regimen in this trial: ISTp-DP was not associated with improvements in the composite outcome of small for gestational age, low birthweight, and preterm birth (primary outcome for paucigravidae) and was associated with more malaria at delivery (primary outcome for multigravidae) and more fetal loss. Although the relative increase in malaria risk was modest, this affected an additional eight out every 100 pregnancies. These results suggest that ISTp-DP may not be a suitable alternative strategy to replace IPTp-SP in settings similar to ours and may even predispose to unfavorable pregnancy outcomes in these settings.

The results may not be representative of areas where >10% of parasites harbor the “sextuple mutant” haplotype carrying the *dhps* A581G mutation [29]; however, our efficacy findings are similar to those reported recently from areas in western Kenya [15] with similarly high transmission (malaria prevalence detected by PCR at enrollment 33% versus 44% in this study) and SP resistance (5.8% *dhps* A581G mutation), and are also consistent with two previous non-inferiority trials conducted in West Africa, despite marked geographic differences in prevailing SP resistance, which is low in West Africa [11,12]. In both West African studies, ISTp was non-inferior to IPTp-SP in the reduction in low birthweight among paucigravidae; mean birthweights were higher in the IPTp-SP recipients than in those receiving ISTp-AL (*p* = 0.04) [12], but there was no significant difference compared to those receiving ISTp with amodiaquine-artesunate (*p* = 0.06) [11]. Additionally, the incidence of clinical malaria was higher in the ISTp-AL arm compared to the IPTp-SP arm. This was not observed in our trial, but the trial in western Kenya also observed higher incidence of clinical malaria as well as of plasmodium infection during pregnancy [15].

Because DP has very high anti-parasitic efficacy in Africa [30], the lack of superiority of ISTp-DP may result either from the ineffectiveness of ISTp as a strategy in high malaria transmission areas or from the continued effectiveness of IPTp-SP despite prevalent SP resistance. To this latter point, in our study area in Malawi, 99.5% of parasites harbor the “quintuple mutant” haplotype, but only 2.6%–4% carry the additional *dhps* A581G mutation [29]. Therefore, it is likely that IPTp-SP continued to provide some benefits, as has been observed in settings with similar parasite populations [2,3]. Another factor likely contributing to continued effectiveness of IPTp-SP was our use of the frequent dosing regimen [31] now recommended by WHO, which may mitigate the shortening of posttreatment prophylaxis that results from SP resistance [2]. It would also be of interest to further explore whether SP, which also has broad antimicrobial activity, may have conferred additional protection from other pathogens [32].

It is unlikely that suboptimal dosing or subtherapeutic levels of DP contributed to the non-superior performance of ISTp-DP: each dose was supervised, and there is no evidence that pregnancy alters the pharmacokinetics of DP to a degree that requires dose adjustment [33]. The same DP regimen was shown to be highly effective (PCR-corrected success rate by day 63: 99%) in a concurrent treatment trial conducted by the same team in this area using the same batch [34].

ISTp-DP may also have been ineffective owing to a failure to detect low-level parasitemias, although the biological impact of such infections during pregnancy is unclear [35]. RDTs detected about 45% of the PCR-positive infections in paucigravidae and about 30% in multigravidae, thereby allowing the majority of infections to persist in the placenta. Conceptually, ISTp is intended to prevent both existing infections from progressing and new infections from occurring for up to 6 wk after each DP course. Because only the RDT-positive women receive treatment, many do not benefit from the posttreatment prophylaxis. Furthermore, the infrequency of screening (approximately monthly) in a high transmission setting may have allowed new infections to develop and persist between scheduled visits. These factors combined may explain the higher prevalence of plasmodium infections at the time of delivery in the ISTp-DP arm.

The ineffectiveness of ISTp to prevent malaria in high transmission settings may also explain the higher rate of fetal loss in the ISTp-DP arm (2.6% versus 1.3%), consistent with the results from previous meta-analyses that showed a 1.5-fold higher risk of fetal loss among women randomized to control arms in trials of insecticide-treated nets [36]. The excess risk of fetal loss was not due to an adverse effect of DP, as the risk was highest among women who had never received DP (3.1% versus 2.2%). An alternative explanation could be that the broad antimicrobial effect of SP reduced the risk of fetal loss relative to ISTp [32]. Lastly, the effect could also be a chance finding, as the trial in Kenya did not observe an excess risk of fetal loss in the ISTp arm [15].

Overall, DP was well tolerated, which is consistent with the results of a recent four-arm treatment trial comparing the four fixed-dose ACTs in the case management of malaria in pregnancy [34]. This is important as almost all RDT-positive women in our trial were asymptomatic, and tolerance can be a major factor determining adherence.

ISTp is a labor-intensive strategy, but a separate qualitative substudy using in-depth interviews and focus group discussions showed it was highly acceptable to both patients and clinic staff. Although ISTp requires more frequent blood sampling, women appreciated its importance and the fact that they could be shown the RDT test results, corroborating findings from similar acceptability studies in Ghana [14,37]. The venous sampling at the first antenatal visit was deemed more convenient by women than repeated finger pricks, as it allowed health workers to tests for malaria, anemia, syphilis, and HIV testing with a single blood draw.

Limitations of our trial include the open-label design used. Another limitation is that we were not able to include a third arm with IPTp with DP as there was insufficient safety information for repeat courses of DP available at the time this trial was designed. Approximately 9% of the randomized women did not contribute to the primary outcome of adverse live birth outcome and 12% did not contribute to the primary outcome of plasmodium infection at delivery. However, this loss to follow-up was well balanced between the study arms, with little differences in baseline characteristics between those who contributed to the primary endpoint versus those who did not; thus, this loss to follow-up is unlikely to have biased the findings. The proportion of multigravidae reporting using a bednet the night prior to enrollment was slightly lower in the ISTp-DP arm; however, this did not explain the observed difference in the risk of plasmodium infection at delivery, as all women received an insecticide-treated net on enrollment, and bednet use thereafter was near universal in both arms (99% in each arm).

ISTp-DP was not superior to the existing IPTp-SP regimen in this area with high SP resistance in southern Malawi. These results should be equally relevant to other high endemic areas in east and southern Africa with similar or lower levels of parasite SP resistance. The identification of alternative drugs to replace SP remains a pressing research priority for the control of malaria in pregnancy before levels of SP resistance render IPTp-SP fully ineffective.

## Supporting Information

S1 CONSORT Checklist (DOC)

S1 Abstract French translation of the Abstract and Author Summary. (DOCX)

S1 Fig Subgroup analysis of the effect of ISTp-DP versus IPTp-SP on the composite primary outcome of small for gestational age, low birthweight, or preterm birth. Hb, hemoglobin; RR, relative risk. Season was defined by ranking the average rainfall in the 6 mo prior to delivery.
(TIF)

S2 Fig Subgroup analysis of the effect of ISTp-DP versus IPTp-SP on the primary outcome maternal or placental plasmodium infection at delivery. Hb, hemoglobin; RR, relative risk. Season was defined by ranking the average rainfall in the 6 mo prior to delivery. The *p*-value for the interaction term comparing the treatment effect for maternal or placental plasmodium infection at delivery (any measure) of primigravidae (RR = 1.08) versus multigravidae and secundigravidae pooled (RR = 1.30) was *p* = 0.08.
(TIF)

S3 Fig Maternal patent and subpatent plasmodium infection at delivery by microscopy, RDT, or PCR. *Patent infection defined as PCR-positive and RDT- or microscopy-positive; subpatent infection defined as PCR-positive and RDT- or microscopy-negative. RDTs detected 44.9% of the PCR-positive infections in paucigravidae and 29.2% in multigravidae. The *p*-value for the interaction term depicts the *p*-value for differences in effect size between paucigravidae and multigravidae.
(TIF)

S4 Fig Placental plasmodium infection by microscopy, RDT, PCR, and placental histology. *p-Value by Fisher’s exact test. ^#^PCR-positive or histology-positive (active infection) and RDT- or microscopy-positive (patent) or RDT- or microscopy-negative (subpatent). The *p*-value for the interaction term depicts the *p*-value for differences in effect size between paucigravidae and multigravidae.
(TIF)

S5 Fig Incidence of infant morbidity by the end of follow-up at 6–8 wk. Data in columns on the left represent the number of infants with an event, the number of events/person-time of follow-up, and, in parentheses, the incidence rate per 100 person-years. *p-Value by Fisher’s exact test. The *p*-value for the interaction term depicts the *p*-value for differences in effect size of infants born to paucigravidae versus multigravidae. In addition, the study was designed to collect information on the incidence of symptomatic severe anemia in the infants up to the age of 6–8 wk (registered outcome 21; S3 Text), but no events occurred.
(TIF)

S1 Table Adherence to follow-up visit schedule and number of courses received by pregnant women (intention to treat population). (DOCX)

S2 Table Proportion of women with missing data for the primary outcomes, by treatment arm. (DOCX)

S3 Table Baseline characteristics comparing women that contributed to the primary endpoint versus those that did not (missing). (DOCX)

S4 Table Mutant allele frequencies in *Plasmodium falciparum* parasites collected at study enrollment by study site and overall. (DOCX)

S5 Table Intention to treat analysis population: covariate-adjusted analysis of primary endpoint, with and without imputation for missing variables. (DOCX)

S6 Table Per protocol analysis population: unadjusted and covariate-adjusted analysis of primary endpoint, with missing values for covariates imputed. (DOCX)

S7 Table Sensitivity analysis to determine the effect of the use of corrected versus uncorrected birthweight, using the intention to treat analysis population. (DOCX)

S8 Table Effect of ISTp-DP versus IPTp-SP on mean maternal hemoglobin, birthweight, gestational age, and birthweight-for-gestational age *Z*-score (crude analysis). (DOCX)

S9 Table Adherence and tolerance of study drugs and regimen by pregnant women. (DOCX)

S10 Table Fetal loss and perinatal and infant death among DP recipients and non-recipients in the ISTp-DP arm, compared with the IPTp-SP arm (post hoc analysis). (DOCX)

S1 Text Supplementary methods. (DOCX)

S2 Text Prespecified statistical analysis plan. (PDF)

S3 Text Study protocol. (PDF)

S4 Text Trial registration. (PDF)

## Abbreviations

ACT: artemisinin-based combination therapy
AL: artemether-lumefantrine
DP: dihydroartemisinin-piperaquine
IPTp: intermittent preventive therapy in pregnancy
IPTp-SP: intermittent preventive therapy in pregnancy with sulfadoxine-pyrimethamine
ISTp: intermittent screening and treatment in pregnancy
ISTp-AL: intermittent screening and treatment in pregnancy with artemether-lumefantrine
ISTp-DP: intermittent screening and treatment in pregnancy with dihydroartemisinin-piperaquine
ITT: intention to treat
LSTM: Liverpool School of Tropical Medicine
RDT: rapid diagnostic test; RR, relative risk
SAE: serious adverse event
SP: sulfadoxine-pyrimethamine
