# Supplementary material for: Scheduled Intermittent Screening with Rapid Diagnostic Tests and Treatment with Dihydroartemisinin-Piperaquine versus Intermittent Preventive Therapy with Sulfadoxine-Pyrimethamine for Malaria in Pregnancy in Malawi: An Open-Label Randomized Controlled Trial
Source: PLoS Med. 2016 Sep 13;13(9):e1002124. doi: 10.1371/journal.pmed.1002124 (PMC5021271; doi:10.1371/journal.pmed.1002124)
Supplement: S1 CONSORT Checklist — (DOC) [file pmed.1002124.s001.doc]

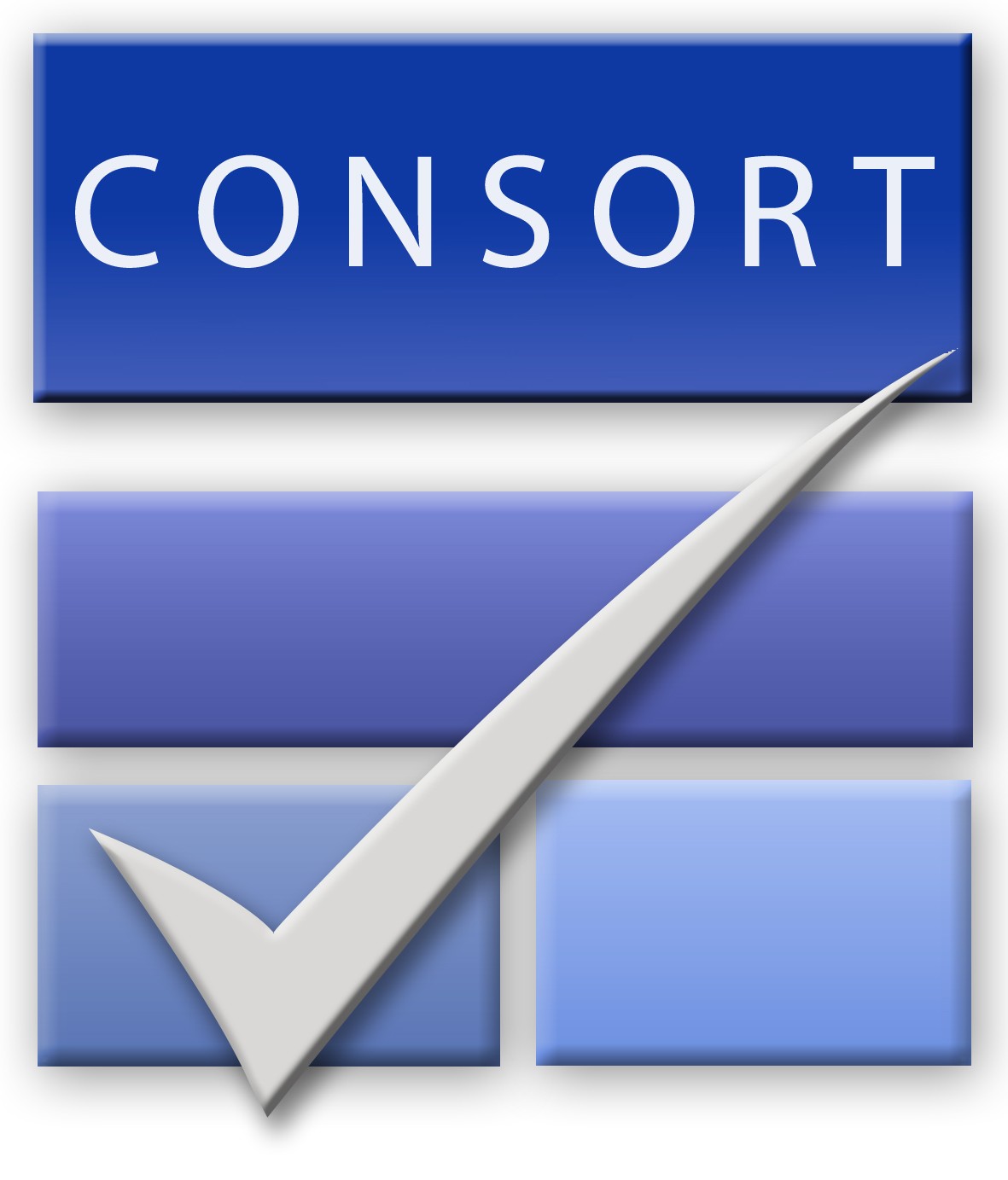
CONSORT 2010 checklist of information to include when reporting a randomised trial*

| Section/Topic | Item No | Checklist item | Reported on page No |
| --- | --- | --- | --- |
| Title and abstract | | | |
|  | 1a | Identification as a randomised trial in the title | Title page |
|  | 1b | Structured summary of trial design, methods, results, and conclusions (for specific guidance see CONSORT for abstracts) | Abstract page |
| Introduction | | | |
| Background and objectives | 2a | Scientific background and explanation of rationale | Introduction paragraph 1&2 |
|  | 2b | Specific objectives or hypotheses | Introduction last para |
| Methods | | | |
| Trial design | 3a | Description of trial design (such as parallel, factorial) including allocation ratio | Methods, study design and participants, paragraph 1 |
|  | 3b | Important changes to methods after trial commencement (such as eligibility criteria), with reasons | None, other than point 6b below |
| Participants | 4a | Eligibility criteria for participants | Methods, study design and participants: paragraph 2 |
|  | 4b | Settings and locations where the data were collected | Methods, study design and participants, paragraph 1 |
| Interventions | 5 | The interventions for each group with sufficient details to allow replication, including how and when they were actually administered | Methods, procedures: paragraph 2 |
| Outcomes | 6a | Completely defined pre-specified primary and secondary outcome measures, including how and when they were assessed | Methods, outcome, paragraph 1 and 2 |
|  | 6b | Any changes to trial outcomes after the trial commenced, with reasons | S1 Text,  1.Definitions of morbidity endpoints, paragraph Small for gestational age (SGA) &  2.Laboratory methods, paragraph hemoglobin concentrations |
| Sample size | 7a | How sample size was determined | Methods, statistical analysis: paragraph 1 and S1 Text Supplementary methods: Sample size calculations, paragraph 1 |
|  | 7b | When applicable, explanation of any interim analyses and stopping guidelines | Not applicable |
| Randomisation: |  |  |  |
| Sequence generation | 8a | Method used to generate the random allocation sequence | Methods: paragraph ‘Randomization and masking’ |
|  | 8b | Type of randomisation; details of any restriction (such as blocking and block size) | Methods: paragraph ‘Randomization and masking’ |
| Allocation concealment mechanism | 9 | Mechanism used to implement the random allocation sequence (such as sequentially numbered containers), describing any steps taken to conceal the sequence until interventions were assigned | Methods: paragraph ‘Randomization and masking’ |
| Implementation | 10 | Who generated the random allocation sequence, who enrolled participants, and who assigned participants to interventions | Methods: paragraph ‘Randomization and masking’ |
| Blinding | 11a | If done, who was blinded after assignment to interventions (for example, participants, care providers, those assessing outcomes) and how | Methods: paragraph ‘Randomization and masking’ |
|  | 11b | If relevant, description of the similarity of interventions | Not applicable (open label) |
| Statistical methods | 12a | Statistical methods used to compare groups for primary and secondary outcomes | Methods, paragraph ‘Statistical analysis’ |
|  | 12b | Methods for additional analyses, such as subgroup analyses and adjusted analyses | Methods, paragraph ‘Statistical analysis’ |
| Results | | | |
| Participant flow (a diagram is strongly recommended) | 13a | For each group, the numbers of participants who were randomly assigned, received intended treatment, and were analysed for the primary outcome | Results: paragraph ‘Baseline and patient disposition’ and Fig 1, and S1 Table |
|  | 13b | For each group, losses and exclusions after randomisation, together with reasons | Results: paragraph ‘Baseline and patient disposition’, and S1 Table |
| Recruitment | 14a | Dates defining the periods of recruitment and follow-up | Results: paragraph ‘Baseline and patient disposition’ |
|  | 14b | Why the trial ended or was stopped | Results: paragraph ‘Baseline and patient disposition’ |
| Baseline data | 15 | A table showing baseline demographic and clinical characteristics for each group | Table 1 |
| Numbers analysed | 16 | For each group, number of participants (denominator) included in each analysis and whether the analysis was by original assigned groups | Fig 1 |
| Outcomes and estimation | 17a | For each primary and secondary outcome, results for each group, and the estimated effect size and its precision (such as 95% confidence interval) | Fig2, Fig3, Fig4, S3 Fig, S4 Fig, S5 Fig, S5 Table to S10 Table |
|  | 17b | For binary outcomes, presentation of both absolute and relative effect sizes is recommended | Fig2 |
| Ancillary analyses | 18 | Results of any other analyses performed, including subgroup analyses and adjusted analyses, distinguishing pre-specified from exploratory | S1 Fig and S2 Fig  S5 Table to S10 Table  Pre-specified statement: Methods, paragraph ‘Statistical analysis’, one but last sentence, and S10 Table note |
| Harms | 19 | All important harms or unintended effects in each group (for specific guidance see CONSORT for harms) | S9 Table, S10 Table |
| Discussion | | | |
| Limitations | 20 | Trial limitations, addressing sources of potential bias, imprecision, and, if relevant, multiplicity of analyses | Discussion, one but last paragraph |
| Generalisability | 21 | Generalisability (external validity, applicability) of the trial findings | Discussion, Paragraph 2 |
| Interpretation | 22 | Interpretation consistent with results, balancing benefits and harms, and considering other relevant evidence | Discussion, Paragraph 2, and final concluding paragraph |
| Other information | | |  |
| Registration | 23 | Registration number and name of trial registry | Abstract page |
| Protocol | 24 | Where the full trial protocol can be accessed, if available | S3 Text, referred to in S5 Table note, and S1 text |
| Funding | 25 |  | See funding statements entered in the online submission forms |

*We strongly recommend reading this statement in conjunction with the CONSORT 2010 Explanation and Elaboration for important clarifications on all the items. If relevant, we also recommend reading CONSORT extensions for cluster randomised trials, non-inferiority and equivalence trials, non-pharmacological treatments, herbal interventions, and pragmatic trials. Additional extensions are forthcoming: for those and for up to date references relevant to this checklist, see [www.consort-statement.org](http://www.consort-statement.org/).
