# Supplementary material for: Scheduled Intermittent Screening with Rapid Diagnostic Tests and Treatment with Dihydroartemisinin-Piperaquine versus Intermittent Preventive Therapy with Sulfadoxine-Pyrimethamine for Malaria in Pregnancy in Malawi: An Open-Label Randomized Controlled Trial
Source: PLoS Med. 2016 Sep 13;13(9):e1002124. doi: 10.1371/journal.pmed.1002124 (PMC5021271; doi:10.1371/journal.pmed.1002124)
Supplement: S1 Abstract — (DOCX) [file pmed.1002124.s002.docx]

# Résumé

Contexte: En Afrique, la plupart des infections à Plasmodium pendant la grossesse restent asymptomatiques, et sont pourtant associées à l’anémie maternelle et au petit poids de naissance. L’OMS recommande un traitement préventif intermittent pendant la grossesse à la sulphadoxine-pyriméthamine (TPI-SP). Cependant, l’efficacité de SP est compromise à cause de la haute résistance des parasites. Nous avons conduit une étude pour évaluer l’efficacité et la sécurité d’emploi du dépistage intermittent préventif avec les tests de diagnostic rapide du paludisme (TDR) et du traitement des femmes TDR-positives avec de la dihydroartémisinine-pipéraquine (DP) comme alternative à la stratégie de TPI-SP.

Méthodes et résultats: Nous avons mené un essai clinique ouvert, de supériorité, avec deux groupes randomisés chez les femmes HIV-séronégatives dans 3 régions du Malawi avec une grande résistance à la SP. L’intervention a consisté en 3 à 4 visites pendant les 2^nd^ et 3^ème^ trimestres, avec 4 à 6 semaines d’intervalle. Le groupe TPI-SP a reçu SP à chaque visite. L’autre groupe a été dépisté pour le paludisme à chaque visite et traité avec DP si le test de diagnostic rapide était positif. Les principaux critères d’évaluation ont été les issues défavorables de l’accouchement chez les primigestes (le faible développement par rapport à l’âge gestationnel, le petit poids de naissance [<2500gr] ou la naissance prématurée [<37s]) et l’infection palustre à la naissance chez les multigestes. L’analyse a été faite en intention-de-traiter.

Entre le 21 juillet 2011 et le 18 mars 2013, 1873 femmes ont été recruté (1155 primi-secundigestes [paucigestes], 718 multigestes). La prévalence des issues défavorables de l’accouchement était similaire dans le groupe dépistage puis traitement avec la DP (29.9%) et le groupe TPI-SP (28.8%) (Différence des risques : 1,08% (Intervalle de Confiance à 95% [IC] : -3,25-5,41) ; Risque relatif (RR)=1,04 (0,90-1,20), p=0,625, (paucigeste: RR=1,10 [0,92-1,31], p=0.282 ; multigeste: RR=0,92 [0,71-1,20], p=0,543). La prévalence du paludisme à l’accouchement était plus élevé dans le groupe TPI-SP (48,7% vs. 40,8%): Différence des risques=7,85 (3,07-12,63) ; RR=1,19 (1,07-1,33), p=0,007 (paucigeste: RR=1,16 [1,04-1,31], p=0,011; multigeste: RR=1,29 [1,02-1,63], p=0,037). Le décès fœtal était plus présent dans le groupe de dépistage et traitement avec DP (2,6% vs 1,3%; RR=2,06 [1,01-4,21], p=0,046) et plus élevé parmi les non-receveurs de DP (3,1%). La conception ouverte de l’étude est une de ses limites.

Conclusions: Le dépistage planifié des parasites du paludisme avec la nouvelle génération de TDR réalisé 3 à 4 fois pendant la grossesse dans le cadre des soins prénataux n’était pas supérieur au TPI-SP dans cette région de haute transmission palustre et de haute résistance à la SP et est associé à un taux plus élevé de décès fœtaux et d’infections palustre à l’accouchement.

Enregistrement de l’*é*tude: PACTR.org: PACTR201103000280319; ISRCTN.com: ISRCTN69800930

Author’s summary (Résumé)

Pourquoi avoir réalisé l’étude?

- Le paludisme pendant la grossesse peut avoir des conséquences dévastatrices sur la mère et l’enfant.
- Le traitement préventif intermittent pendant la grossesse (TPI) à la sulphadoxine-pyriméthamine (SP) est l’une des principales interventions pour protéger la femme enceinte pendant la grossesse dans les zones endémiques d’Afrique subsaharienne.
- L’efficacité de la SP est cependant menacée par la résistance croissante du parasite dans l’est et le sud de l’Afrique.
- Nous avons mené cette étude pour évaluer si une stratégie alternative consistant à dépister les femmes enceintes avec les tests de diagnostic rapides régulièrement pendant la grossesse et traiter les cas positifs avec de la dihydroartémisinine-pipéraquine (DP), un antipaludique très efficace, pourrait réduire le risque de l’infection palustre et ses conséquences chez la mère et l’enfant. Cette stratégie est nommée le dépistage et traitement intermittents pendant la grossesse.

Ce que les chercheurs ont fait et trouvé?

- Notre équipe a réalisé un essai clinique pour comparer l’impact de la nouvelle stratégie de dépistage et traitement avec DP par rapport à l’existant TPI avec SP (groupe contrôle) parmi 1873 femmes enceintes dans le sud du Malawi, région où presque la totalité des parasites sont très résistants à SP.
- Notre étude a montré que la transmission du paludisme était élevée dans les deux groupes et que la nouvelle stratégie de dépistage et traitement avec DP n’était pas meilleur que la stratégie existante TPI avec SP en ce qui concerne la réduction de l’infection palustre et l’amélioration de la grossesse ; ainsi, les femmes dans le groupe de dépistage et traitement avec DP avaient plus le paludisme que celles dans le groupe de TPI avec SP.

Que signifient ces résultats?

- Le dépistage et traitement avec la génération actuelle des diagnostics de tests rapides n’est pas une stratégie viable pour remplacer le TPI avec SP dans les régions endémiques d’Afrique subsaharienne, malgré les taux élevés de résistance à SP.
- TPI avec SP peut encore être utilisé dans la lutte contre le paludisme en Afrique subsaharienne.
- Des études supplémentaires sur les traitements alternatifs à SP pour le TPI doivent être conduites dans ces régions où la résistance à SP est élevée.
