# Supplementary material for: Scheduled Intermittent Screening with Rapid Diagnostic Tests and Treatment with Dihydroartemisinin-Piperaquine versus Intermittent Preventive Therapy with Sulfadoxine-Pyrimethamine for Malaria in Pregnancy in Malawi: An Open-Label Randomized Controlled Trial
Source: PLoS Med. 2016 Sep 13;13(9):e1002124. doi: 10.1371/journal.pmed.1002124 (PMC5021271; doi:10.1371/journal.pmed.1002124)
Supplement: S1 Table — (DOCX) [file pmed.1002124.s008.docx]

| **S1 Table: Adherence to follow-up visit schedule and number of courses received by pregnant women (intention to treat population)** | | | | | | | | |
| --- | --- | --- | --- | --- | --- | --- | --- | --- |
|  | **Paucigravidae** | |  | **Multigravidae** | |  | **All gravidae** | |
|  | **ISTp-DP** | **IPTp-SP** |  | **ISTp-DP** | **IPTp-SP** |  | **ISTp-DP** | **IPTp-SP** |
|  | **(n=571)** | **(n=569)** |  | **(n=352)** | **(n=352)** |  | **(n=923)** | **(n=921)** |
| **Planned No. of scheduled visits, including enrolment, excluding delivery,^a^ No. (%)** | | | | | | | | |
| 3 | 58 (10.2) | 58 (10.2) |  | 50 (14.2) | 48 (13.6) |  | 108 (11.7) | 106 (11.5) |
| 4 | 513 (89.8) | 511 (89.8) |  | 302 (85.8) | 304 (86.4) |  | 815 (88.3) | 815 (88.5) |
| Total visits | 2226 | 2218 |  | 1358 | 1360 |  | 3584 | 3578 |
| **Possible No. of scheduled visits adjusted for early delivery, including enrolment, excluding delivery,^b^ No. (%)** | | | | | | | | |
| 1 | 4 (0.4) | 5 (0.9) |  | 0 (0) | 0 (0) |  | 4 (0.4) | 5 (0.9) |
| 2 | 19 (3.3) | 15 (2.6) |  | 11 (3.1) | 8 (2.3) |  | 30 (3.3) | 23 (2.5) |
| 3 | 90 (15.8) | 79 (13.9) |  | 64 (18.2) | 68 (19.3) |  | 154 (16.7) | 147 (16.0) |
| 4 | 458 (80.2) | 470 (82.6) |  | 277 (78.7) | 276 (78.4) |  | 735 (79.6) | 746 (81.0) |
| Total visits | 2144 | 2152 |  | 1322 | 1324 |  | 3466 | 3476 |
| **Achieved number of scheduled visits, including enrolment, excluding delivery, No. (%)** | | | | | | | | |
| 1 | 21 (3.7) | 27 (4.7) |  | 12 (3.4) | 8 (2.3) |  | 33 (3.6) | 35 (3.8) |
| 2 | 38 (6.7) | 34 (6.0) |  | 22 (6.3) | 26 (7.4) |  | 60 (6.5) | 60 (6.5) |
| 3 | 133 (23.3) | 115 (20.2) |  | 80 (22.7) | 100 (28.4) |  | 213 (23.1) | 215 (23.3) |
| 4 | 379 (66.4) | 393 (69.1) |  | 238 (67.6) | 218 (61.9) |  | 617 (66.8) | 611 (66.3) |
| Total visits | 2012 | 2012 |  | 1248 | 1232 |  | 3260 | 3244 |
| **Number of DP or SP/AL courses received (scheduled or unscheduled visits), No. (%)** | | | | | | | | |
| 0 | 220 (38.5) | 1 (0.2) |  | 230 (65.3) | 0 (0) |  | 450 (48.8) | 1 (0.1) |
| 1 | 244 (42.7) | 28 (4.9) |  | 106 (30.1) | 11 (3.1) |  | 350 (37.9) | 39 (4.2) |
| 2 | 99 (17.3) | 35 (6.1) |  | 16 (4.6) | 26 (7.4) |  | 115 (12.5) | 61 (6.6) |
| 3 | 8 (1.4) | 113 (19.9) |  | 0 | 101 (28.7) |  | 8 (0.9) | 214 (23.2) |
| 4 | 0 | 344 (60.5) |  | 0 | 201 (57.1) |  | 0 | 545 (59.2) |
| 5+ | 0 | 48 (8.4) |  | 0 | 13 (3.7) |  | 0 | 61 (6.6) |
| DP/SP courses received | 466 | 1863 |  | 138 | 1185 |  | 604 | 3048 |
| AL courses received | 0 | 199 |  | 0 | 52 |  | 0 | 251 |
| Total courses received | 466 | 2062 |  | 138 | 1237 |  | 604 | 3299 |
| **Person days contributed till delivery or till lost to follow-up, median (IQR)** | | | | | | | | |
|  | 117  (97-143) | 117  (102-142) |  | 115  (98-139) | 116  (95-141) |  | 116  (97-142) | 117  (99-142) |
| 1. The number of scheduled visits was dependent on the gestational age at enrolment. This was either 4 visits (including enrolment) if women were enrolled between 16 to 25 weeks gestation, or 3 visits if they were enrolled between 26 and 28 weeks gestation. 2. Adjusted for early delivery (i.e. excludes all planned antenatal visits that could not have occurred because the pregnancy ended before that scheduled date)   IQR=interquartile range | | | | | | | | |
