# Supplementary material for: Scheduled Intermittent Screening with Rapid Diagnostic Tests and Treatment with Dihydroartemisinin-Piperaquine versus Intermittent Preventive Therapy with Sulfadoxine-Pyrimethamine for Malaria in Pregnancy in Malawi: An Open-Label Randomized Controlled Trial
Source: PLoS Med. 2016 Sep 13;13(9):e1002124. doi: 10.1371/journal.pmed.1002124 (PMC5021271; doi:10.1371/journal.pmed.1002124)
Supplement: S2 Table — (DOCX) [file pmed.1002124.s009.docx]

| **S2 Table: Proportion of women with missing data for the primary outcome, by treatment arm** | | | | |
| --- | --- | --- | --- | --- |
|  | | **no/No (%) of patients with missing primary endpoint** | | **Risk Ratio (95% CI), p-value** |
| **Outcome** | | **ISTp-DP** | **IPTp-SP** |  |
| **Primary endpoint gravidity specific*** | | |  |  |
|  | Paucigravidae | 57/576 (9.9) | 53/579 (9.2) | 1.08 (0.76, 1.54), 0.668 |
|  | Multigravidae | 43/361 (11.9) | 44/357 (12.3) | 0.97 (0.65, 1.43), 0.865 |
|  | All gravidae | 100/937 (10.7) | 97/936 (10.4) | 1.03 (0.79, 1.34), 0.827 |
| **SGA/LBW/PT** | |  |  |  |
|  | Paucigravidae | 57/576 (9.9) | 53/579 (9.2) | 1.08 (0.76, 1.54), 0.668 |
|  | Multigravidae | 31/361 (8.6) | 30/357 (8.4) | 1.02 (0.63, 1.66), 0.930 |
|  | All gravidae | 88/937 (9.4) | 83/936 (8.9) | 1.06 (0.80, 1.41), 0.694 |
| **Malaria infection at delivery** | | |  |  |
|  | Paucigravidae | 64/576 (11.1) | 69/579 (11.9) | 0.93 (0.68, 1.29), 0.668 |
|  | Multigravidae | 43/361 (11.9) | 44/357 (12.3) | 0.97 (0.65, 1.43), 0.865 |
|  | All gravidae | 107/937 (11.4) | 113/936 (12.1) | - 1. (0.74, 1.21), 0.661 |
| *For paucigravidae this endpoint is SGA/LBW/PT and for multigravidae it is malaria infection at delivery | | | | |
