# Supplementary material for: Scheduled Intermittent Screening with Rapid Diagnostic Tests and Treatment with Dihydroartemisinin-Piperaquine versus Intermittent Preventive Therapy with Sulfadoxine-Pyrimethamine for Malaria in Pregnancy in Malawi: An Open-Label Randomized Controlled Trial
Source: PLoS Med. 2016 Sep 13;13(9):e1002124. doi: 10.1371/journal.pmed.1002124 (PMC5021271; doi:10.1371/journal.pmed.1002124)
Supplement: S3 Table — (DOCX) [file pmed.1002124.s010.docx]

| **S3 Table: Baseline characteristics comparing women that contributed to the primary endpoint versus those that did not (missing)** | | | | | | | | | |  |
| --- | --- | --- | --- | --- | --- | --- | --- | --- | --- | --- |
|  |  | **Paucigravidae (N=1,055)** | | | **Multigravidae (N=718)** | | | **All gravidae (pooled, N=1,873)** | | |
|  |  | **Contributes** | **Missing** |  | **Contributes** | **Missing** |  | **Contributes** | **Missing** |  |
|  |  | **(n=1,046)** | **(n=109)** | **P** | **(n=631)** | **(n=87)** | **P** | **(n=1,676)** | **(n=197)** | **P** |
| **Maternal characteristics** | |  |  |  |  |  |  |  |  |  |
| Study site | |  |  |  |  |  |  |  |  |  |
|  | Madziabango | 21.7% (227/1,045) | 25.5% (28/110) | 0.425 | 30.1% (190/631) | 34.5% (30/87) | 0.311 | 24.9% (417/1,676) | 29.40% (58/197) | 0.086 |
|  | Mpemba | 51.2% (535/1,045) | 52.7% (58/110) |  | 54.8% (346/631) | 56.3% (49/87) |  | 52.6% (881/1,676) | 54.3% (107/197) |  |
|  | Chikwawa | 27.1% (283/1,045) | 21.8% (24/110) |  | 15.1% (95/631) | 9.2% (8/87) |  | 22.6% (378/1,676) | 16.2% (32/197) |  |
| Maternal age (years) | | 19.6 (2.8) | 19.7 (2.9) | 0.522 | 27.4 (4.3) | 27.1 (4.0) | 0.330 | 22.5 (5.1) | 22.9 (5.1) | 0.320 |
| Marital status | |  |  |  |  |  |  |  |  |  |
|  | Single | 8.5% (89/1,044) | 6.4% (7/110) | 0.435 | 1.3% (8/631) | 1.2% (1/86) | 0.811 | 5.8% (97/1,675) | 4.1% (8/196) | 0.514 |
|  | Married | 91.5% (955/1,044) | 93.6% (102/110) |  | 98.3% (620/631) | 98.8% (85/86) |  | 94.0% (1,575/1,675) | 95.9% (188/196) |  |
|  | Widowed/separated/divorced | 0.0% (0/1,044) | 0.0% (0/110) |  | 0.5% (3/631) | 0.0% (0/86) |  | 0.2% (3/1,675) | 0.0% (0/196) |  |
| Used a bednet last night | | 17.6% (184/1,045) | 17.3% (19/110) | 0.930 | 21.2% (134/631) | 22.1% (19/86) | 0.856 | 19.0% (318/1,676) | 19.4% (38/196) | 0.889 |
| Schooling (years completed) | | 6.7 (3.3) | 6.6 (3.9) | 0.610 | 4.4 (3.7) | 4.7 (3.6) | 0.569 | 5.9 (3.6) | 5.7 (3.9) | 0.625 |
| SES index score (terciles) | |  |  |  |  |  |  |  |  |  |
|  | Low | 34.9% (364/1,042) | 25.5% (28/110) | 0.060 | 32.0% (202/631) | 33.7% (29/86) | 0.808 | 33.8% (566/1,673) | 29.1% (57/196) | 0.354 |
|  | Medium | 31.6% (329/1,042) | 30.9% (34/110) |  | 36.6% (231/631) | 38.4% (33/86) |  | 33.5% (560/1,673) | 34.2% (67/196) |  |
|  | High | 33.5% (349/1,042) | 43.6% (48/110) |  | 31.4% (198/631) | 27.9% (24/86) |  | 32.7% (547/1,673) | 36.7% (72/196) |  |
| Rainfall (average mm/month) 3 months before enrolment (median, IQR) | | 22.8 (3.0-117.5) | 19.2 (3.3-85.3) | 0.827 | 24.7 (3.8-117.5) | 15.6 (3.3-81.2) | 0.040 | 22.8 (3.3-117.5) | 15.6 (3.3-85.3) | 0.151 |
| Pregnancy number (gravidity) | |  |  |  |  |  |  |  |  |  |
|  | First | 54.6% (571/1,045) | 59.1% (65/110) | 0.372 | NA | NA | 0.176 | 34.1% (571/1,674) | 33.2% (65/196) | 0.124 |
|  | Second | 45.4% (474/1,045) | 40.9% (45/110) |  | NA | NA |  | 28.3% (474/1,674) | 23.0% (45/196) |  |
|  | Third | NA | NA |  | 42.3% (266/629) | 50.0% (43/86) |  | 15.9% (266/1,674) | 21.9% (43/196) |  |
|  | Fourth or higher | NA | NA |  | 57.7% (363/629) | 50.0% (43/86) |  | 21.7% (363/1,674) | 21.9% (43/196) |  |
| Gestational age by ultrasound (days) | | 144.7 (21.0) | 143.4 (21.0) | 0.537 | 148.8 (22.2) | 145.6 (36.7) | 0.249 | 146.3 (21.5) | 144.4 (28.9) | 0.265 |
| Had a previous stillbirth/abortions | | 9.5% (46/486) | 15.2% (7/46) | 0.213 | 12.4% (78/631) | 15.1% (13/86) | 0.472 | 11.1% (124/1,117) | 15.2% (20/132) | 0.168 |
| Maternal weight (kg) | | 54.2 (6.8) | 55.3 (8.1) | 0.096 | 56.7 (8.1) | 56.4 (7.3) | 0.800 | 55.1 (7.4) | 55.8 (7.7) | 0.213 |
| Maternal height (cm) | | 153.8 (4.8) | 154.4 (5.7) | 0.187 | 154.4 (5.2) | 154.4 (5.0) | 0.900 | 154.0 (4.9) | 154.4 (5.4) | 0.249 |
| **Laboratory findings** | |  |  |  |  |  |  |  |  |  |
| Hemoglobin (g/dL) | | 10.7 (1.5) | 10.7 (1.5) | 0.723 | 11.5 (1.3) | 11.3 (1.3) | 0.146 | 11.0 (1.4) | 11.0 (1.4) | 0.864 |
| Plasmodium infection | |  |  |  |  |  |  |  |  |  |
|  | RDT (ISTp arm) | 44.7% (232/519) | 31.6% (18/57) | 0.058 | 19.2% (61/318) | 16.7% (7/42) | 0.695 | 35.0% (293/837) | 25.3% (25/99) | 0.053 |
|  | Microscopy | 19.2% (198/1,033) | 18.4% (20/109) | 0.836 | 10.7% (67/626) | 9.3% (8/86) | 0.860 | 16.0% (265/1,659) | 14.4% (28/195) | 0.559 |
|  | PCR | 52.1% (533/1,023) | 43.0% (46/107) | 0.073 | 31.1% (192/618) | 30.6% (26/85) | 0.929 | 44.2% (725/1,641) | 37.5% (72/192) | 0.077 |
|  | Microscopy or PCR | 54.2% (566/1,044) | 45.6% (50/110) | 0.080 | 33.9% (214/631) | 35.6% (31/87) | 0.751 | 46.6% (780/1,675) | 41.1% (81/197) | 0.147 |
| Parasite density^a^ (median, IQR) | | 1,653 (320-7,733) | 2,387 (640-9,867) | 0.389 | 400 (160-3,280) | 2,720 (640-12,533) | 0.106 | 1,040 (267-6,400) | 2,413 (640-9,867) | 0.112 |
| Data are % (n/N) or mean (SD), unless otherwise indicated.  SES=Socioeconomic Status, mm=millimeters rainfall (average per month), kg=kilograms, cm=centimeter, g/dL:=grams per deciliter, RDT=Rapid diagnostic test for malaria, PCR=Polymerase Chain Reaction, NA=Not Applicable  a. Parasite density per microliter assessed by microscopy assuming a count of 8000 white blood cells per microliter | | | | | | | | | | |
