# Supplementary material for: Scheduled Intermittent Screening with Rapid Diagnostic Tests and Treatment with Dihydroartemisinin-Piperaquine versus Intermittent Preventive Therapy with Sulfadoxine-Pyrimethamine for Malaria in Pregnancy in Malawi: An Open-Label Randomized Controlled Trial
Source: PLoS Med. 2016 Sep 13;13(9):e1002124. doi: 10.1371/journal.pmed.1002124 (PMC5021271; doi:10.1371/journal.pmed.1002124)
Supplement: S4 Table — (DOCX) [file pmed.1002124.s011.docx]

| **S4 Table: Mutant allele frequencies in P. falciparum parasites collected at study enrollment by study site and allocation arm** | | | | |
| --- | --- | --- | --- | --- |
| *Gene*, mutation | Madziabango | Mpemba | Chikwawa | Aggregate |
|  | (n=230) | (n=414) | (n=156) | (n=800) |
| *dhfr*, % (n/N) |  |  |  |  |
| N51**I** | 100.0% | 99.8% | 99.9% | 99.9% |
|  | (6755/6758) | (2616/2620) | (3147/3151) | (12518/12529) |
| C59**R** | 99.9% | 100.0% | 99.7% | 99.9% |
|  | (6780/6785) | (3504/3505) | (5110/5127) | (15394/15417) |
| S108**N** | 98.3% | 99.7% | 99.3% | 99.3% |
|  | (290/295) | (607/609) | (720/725) | (1617/1629) |
| *dhps,* % (n/N) |  |  |  |  |
| A437**G** | 98.7% | 99.9% | 99.9% | 99.4% |
|  | (7523/7620) | (5700/5707) | (3994/3998) | (17217/17325) |
| K540**E** | 99.2% | 99.8% | 99.7% | 99.5% |
|  | (7290/7346) | (5094/5104) | (3534/3546) | (15918/15996) |
| A581**G** | 3.0% | 3.4% | 1.0% | 2.7% |
|  | (209/6944) | (192/5570) | (38/3639) | (439/16153) |
| In the first column, n indicates the number of sequencing reads harbouring the substitution of interest and N the number of sequencing reads covering that locus. In the first row, n indicates the number of *P. falciparum* parasites input into the analysis. All *dhfr* I164**L** and *dhps* S436**A** and A613**T** frequencies were < 0.1%. | | | | |
|  |  |  |  |  |
