# Supplementary material for: Scheduled Intermittent Screening with Rapid Diagnostic Tests and Treatment with Dihydroartemisinin-Piperaquine versus Intermittent Preventive Therapy with Sulfadoxine-Pyrimethamine for Malaria in Pregnancy in Malawi: An Open-Label Randomized Controlled Trial
Source: PLoS Med. 2016 Sep 13;13(9):e1002124. doi: 10.1371/journal.pmed.1002124 (PMC5021271; doi:10.1371/journal.pmed.1002124)
Supplement: S5 Table — (DOCX) [file pmed.1002124.s012.docx]

| **S5 Table: Intention to treat analysis population, co-variate adjusted analysis of primary endpoint, with and without imputation for missing variables** | | | | | | | |
| --- | --- | --- | --- | --- | --- | --- | --- |
|  | | **no/No (%) of patients with events** | | **Co-variate adjusted analysis**  **No imputation for missing values** | | **Co-variate adjusted analysis**  **Missing values for co-variates imputed** | |
| **Outcome** | | **ISTp-DP** | **IPTp-SP** | **Risk Ratio**  **(95% CI), p-value** | **Risk Difference**  **(95% CI), p-value** | **Risk Ratio**  **(95% CI), p-value** | **Risk Difference**  **(95% CI), p-value** |
| **SGA/LBW/PT** | |  |  |  |  |  |  |
|  | Paucigravidae | 175/519 (33.7) | 161/526 (30.6) | 1.10 (0.93, 1.31), 0.2727 | 2.17 (-3.34, 7.69), 0.4399 | 1.10 (0.93, 1.31), 0.2552 | 2.27 (-3.23, 7.77), 0.4189 |
|  | Multigravidae | 79/330 (23.9) | 85/327 (26.0) | 0.89 (0.68, 1.16), 0.3778 | -3.96 (-10.55, 2.63), 0.2391 | 0.89 (0.68, 1.16), 0.3778 | -3.96 (-10.55, 2.63), 0.2391 |
|  | All gravidae | 254/849 (29.9) | 246/853 (28.8) | 1.03 (0.89, 1.19), 0.6814 | 0.04 (-4.23, 4.32), 0.9856 | 1.03 (0.89, 1.19), 0.6594 | 0.10 (-4.17, 4.37), 0.9638 |
| **Plasmodium infection at delivery** | | |  |  |  |  |  |
|  | Paucigravidae | 293/512 (57.2) | 251/510 (49.2) | 1.13 (1.01, 1.26), 0.0266 | 7.88 (1.97, 13.80), 0.0090 | 1.13 (1.02, 1.27), 0.0237 | 8.03 (2.13, 13.94), 0.0077 |
|  | Multigravidae | 111/318 (34.9) | 85/313 (27.2) | 1.27 (1.01, 1.60), 0.0419 | 8.17 (1.02, 15.32), 0.0252 | 1.27 (1.01, 1.60), 0.0419 | 8.17 (1.02, 15.32), 0.0252 |
|  | All gravidae | 404/830 (48.7) | 336/823 (40.8) | 1.15 (1.04, 1.28), 0.0060 | 7.21 (2.59, 11.84), 0.0022 | 1.15 (1.04, 1.28), 0.0055 | 7.27 (2.65, 11.89), 0.0021 |
|  | | | | | | | |
