# Supplementary material for: Scheduled Intermittent Screening with Rapid Diagnostic Tests and Treatment with Dihydroartemisinin-Piperaquine versus Intermittent Preventive Therapy with Sulfadoxine-Pyrimethamine for Malaria in Pregnancy in Malawi: An Open-Label Randomized Controlled Trial
Source: PLoS Med. 2016 Sep 13;13(9):e1002124. doi: 10.1371/journal.pmed.1002124 (PMC5021271; doi:10.1371/journal.pmed.1002124)
Supplement: S6 Table — (DOCX) [file pmed.1002124.s013.docx]

| **S6 Table: Per protocol analysis population, unadjusted and co-variate adjusted analysis of primary endpoint, with missing values for co-variates imputed** | | | | | | | |
| --- | --- | --- | --- | --- | --- | --- | --- |
|  | | **no/No (%) of patients with events** | | **Unadjusted analysis** | | **Co-variate adjusted analysis** | |
| **Outcome** | | **ISTp-DP** | **IPTp-SP** | **Risk Ratio**  **(95% CI), p-value** | **Risk Difference**  **(95% CI), p-value** | **Risk Ratio**  **(95% CI), p-value** | **Risk Difference**  **(95% CI), p-value** |
| **SGA/LBW/PT** | |  |  |  |  |  |  |
|  | Paucigravidae | 123/439 (28.0) | 109/444 (24.5) | 1.16 (0.93, 1.43), 0.1869 | 3.05 (-2.57, 8.67), 0.2874 | 1.16 (0.94, 1.44), 0.1738 | 3.16 (-2.43, 8.76), 0.2679 |
|  | Multigravidae | 55/276 (19.9) | 55/267 (20.6) | 0.94 (0.68, 1.32), 0.7326 | -2.60 (-9.51, 4.32), 0.4619 | 0.94 (0.68, 1.32), 0.7326 | -2.60 (-9.51, 4.32), 0.4619 |
|  | All gravidae | 178/715 (24.9) | 164/711 (23.1) | 1.08 (0.90, 1.29), 0.4253 | 1.18 (-3.14, 5.51), 0.5917 | 1.08 (0.90, 1.29), 0.4101 | 1.22 (-3.10, 5.55), 0.5789 |
| **Malaria infection at delivery** | |  |  |  |  |  |  |
|  | Paucigravidae | 241/429 (56.2) | 199/428 (46.5) | 1.21 (1.06, 1.38), 0.0048 | 9.83 (3.42, 16.25), 0.0027 | 1.19 (1.05, 1.35), 0.0059 | 10.00 (3.60, 16.40, 0.0022 |
|  | Multigravidae | 88/264 (33.3) | 64/259 (24.7) | 1.35 (1.03, 1.77), 0.0314 | 8.58 (0.76, 16.40), 0.0315 | 1.36 (1.03, 1.78), 0.0273 | 8.58 (0.76, 16.40), 0.0315 |
|  | All gravidae | 329/693 (47.5) | 263/687 (38.3) | 1.24 (1.10, 1.40), 0.0006 | 8.70 (3.69, 13.72), 0.0007 | 1.20 (1.07, 1.35), 0.0017 | 8.77 (3.76, 13.78), 0.0006 |
|  | | | | | | | |
