# Supplementary material for: Scheduled Intermittent Screening with Rapid Diagnostic Tests and Treatment with Dihydroartemisinin-Piperaquine versus Intermittent Preventive Therapy with Sulfadoxine-Pyrimethamine for Malaria in Pregnancy in Malawi: An Open-Label Randomized Controlled Trial
Source: PLoS Med. 2016 Sep 13;13(9):e1002124. doi: 10.1371/journal.pmed.1002124 (PMC5021271; doi:10.1371/journal.pmed.1002124)
Supplement: S7 Table — (DOCX) [file pmed.1002124.s014.docx]

| **S7 Table: Sensitivity analysis to determine the effect of the use of corrected versus uncorrected birthweight, using the intention to treat analysis population** | | | | | | | |
| --- | --- | --- | --- | --- | --- | --- | --- |
|  | | **Corrected birthweight within 7 days of birth** | | | **Un-corrected birthweight measured within 24 hours of delivery** | | |
|  | | **no/No (%) of women with events or**  **No, mean (SD)** | | **Risk Ratio or Mean Difference**  **(95% CI), p-value** | **no/No (%) of women with events or**  **No, mean (SD)** | | **Risk Ratio or Mean Difference**  **(95% CI), p-value** |
| **Outcome** | | **ISTp-DP** | **IPTp-SP** |  | **ISTp-DP** | **IPTp-SP** |  |
| **Birthweight** | |  |  |  |  |  |  |
|  | Paucigravidae | 504; 2,859 (412) | 508; 2,891 (454) | -32.32 (-85.72, 21.08), 0.2355 | 480; 2,853 (406) | 488; 2,887 (453) | -34.81 (-88.93, 19.30), 0.2073 |
|  | Multigravidae | 314; 3,020 (421) | 310; 3,041 (404) | -20.56 (-85.23, 44.11), 0.5333 | 293; 3,010 (418) | 298; 3,035 (403) | -25.03 (-91.13, 41.08), 0.4581 |
|  | All gravidae | 818; 2,921 (423) | 818; 2,948 (442) | -27.07 (-68.95, 14.80), 0.2051 | 773; 2,912 (417) | 786; 2,944 (440) | -31.12 (-73.68, 11.44), 0.1518 |
| **LBW** | |  |  |  |  |  |  |
|  | Paucigravidae | 77/504 (15.3) | 59/508 (11.6) | 1.32 (0.96, 1.80), 0.0890 | 74/480 (15.4) | 56/488 (11.5) | 1.34 (0.97, 1.86), 0.0736 |
|  | Multigravidae | 21/314 (6.7) | 17/310 (5.5) | 1.22 (0.66, 2.27), 0.5303 | 19/293 (6.5) | 16/298 (5.4) | 1.21 (0.63, 2.30), 0.5663 |
|  | All gravidae | 98/818 (12.0) | 76/818 (9.3) | 1.29 (0.97, 1.71), 0.0788 | 93/773 (12.0) | 72/786 (9.2) | 1.31 (0.98, 1.76), 0.0665 |
| **SGA/LBW/PT** | |  |  |  |  |  |  |
|  | Paucigravidae | 175/519 (33.7) | 161/526 (30.6) | 1.10 (0.92, 1.31), 0.2822 | 175/519 (33.7) | 160/526 (30.4) | 1.11 (0.93, 1.32), 0.2535 |
|  | Multigravidae | 79/330 (23.9) | 85/327 (26.0) | 0.92 (0.71, 1.20), 0.5431 | 79/330 (23.9) | 85/327 (26.0) | 0.92 (0.71, 1.20), 0.5431 |
|  | All gravidae | 254/849 (29.9) | 246/853 (28.8) | 1.04 (0.90, 1.20), 0.6254 | 254/849 (29.9) | 245/853 (28.7) | 1.04 (0.90, 1.21), 0.5881 |
| All birthweight in the primary analyses refer to corrected birthweights taken within 7 days (168 hours) after birth. Birthweights taken more than 24 hours after delivery were corrected for the physiological fall in birth weight in breastfed infants occurring in the first days following delivery [1,2]. Birth weights taken 24-48h hours, and 48-168 hours after delivery were corrected by a factor +2% and +4%, respectively to obtain the estimated weight at birth [3,4]. | | | | | | | |

**References**

1. Noel-Weiss J, Courant G, Woodend AK. Physiological weight loss in the breastfed neonate: a systematic review. Open medicine : a peer-reviewed, independent, open-access journal. 2008;2(4):e99-e110. PubMed PMID: 21602959; PubMed Central PMCID: PMC3091615.

2. Flaherman VJ, Kuzniewicz MW, Li S, Walsh E, McCulloch CE, Newman TB. First-day weight loss predicts eventual weight nadir for breastfeeding newborns. Archives of disease in childhood Fetal and neonatal edition. 2013;98(6):F488-92. doi: 10.1136/archdischild-2012-303076. PubMed PMID: 23864443.

3. Greenwood BM, Greenwood AM, Snow RW, Byass P, Bennett S, Hatib-N'Jie AB. The effects of malaria chemoprophylaxis given by traditional birth attendants on the course and outcome of pregnancy. Transactions of the Royal Society of Tropical Medicine and Hygiene. 1989;83(5):589-94. PubMed PMID: 2617619.

4. D'Alessandro U, Langerock P, Bennett S, Francis N, Cham K, Greenwood BM. The impact of a national impregnated bed net programme on the outcome of pregnancy in primigravidae in The Gambia. Transactions of the Royal Society of Tropical Medicine and Hygiene. 1996;90(5):487-92. PubMed PMID: 8944251.
