# Supplementary material for: Scheduled Intermittent Screening with Rapid Diagnostic Tests and Treatment with Dihydroartemisinin-Piperaquine versus Intermittent Preventive Therapy with Sulfadoxine-Pyrimethamine for Malaria in Pregnancy in Malawi: An Open-Label Randomized Controlled Trial
Source: PLoS Med. 2016 Sep 13;13(9):e1002124. doi: 10.1371/journal.pmed.1002124 (PMC5021271; doi:10.1371/journal.pmed.1002124)
Supplement: S8 Table — (DOCX) [file pmed.1002124.s015.docx]

| **S8 Table: Effect of ISTp-DP vs IPTp-SP on maternal mean haemoglobin, birthweight, gestational age and mean birthweight-for gestational age Z-score (crude analysis)** | | | | | |
| --- | --- | --- | --- | --- | --- |
|  | | **Number of women,**  **mean (SD)** | | **Mean difference**  **(95% CI)** | **P-value** |
| **Outcome** | | **ISTp-DP** | **IPTp-SP** |  |  |
| **Maternal haemoglobin (g/dL) last scheduled visit in 3^rd^ trimester** | | | | | |
|  | Paucigravidae | 408, 11.6 (1.3) | 415, 11.4 (1.2) | 0.12 (-0.05,0.29) | 0.165 |
|  | Multigravidae | 262, 11.9 (1.1) | 250, 11.8 (1.2) | 0.09 (-0.11,0.30) | 0.384 |
|  | All gravidae | 670, 11.7 (1.2) | 665, 11.6 (1.2) | 0.11 (-0.02,0.25) | 0.090 |
| **Maternal haemoglobin (g/dL) at delivery** | | | | | |
|  | Paucigravidae | 503, 12.0 (1.6) | 506, 11.7 (1.5) | 0.22 (0.03, 0.41) | 0.026 |
|  | Multigravidae | 314, 12.1 (1.5) | 312, 12.1 (1.5) | -0.09 (-0.33, 0.15) | 0.466 |
|  | All gravidae | 817, 12.0 (1.6) | 818, 11.9 (1.5) | 0.10 (-0.05, 0.25) | 0.179 |
| **Foetal haemoglobin (g/dL) (cord blood)** | | | | | |
|  | Paucigravidae | 472, 15.0 (2.4) | 470, 14.9 (2.1) | 0.10 (-0.18, 0.39) | 0.478 |
|  | Multigravidae | 297, 14.7 (2.3) | 291, 14.9 (2.3) | -0.14 (-0.51, 0.23) | 0.466 |
|  | All gravidae | 769, 14.9 (2.4) | 761, 14.9 (2.2) | 0.01 (-0.22, 0.24) | 0.937 |
| **Mean birthweight (grams)** | | | | | |
|  | Paucigravidae | 504, 2859 (412) | 508, 2891 (454) | -32.3 (-85.7, 21.1) | 0.236 |
|  | Multigravidae | 314, 3020 (421) | 310, 3041 (404) | -20.6 (-85.2, 44.1) | 0.533 |
|  | All gravidae | 818, 2921 (423) | 818, 2948 (442) | -27.1 (-69.0, 14.8) | 0.205 |
| **Gestational age (weeks) at birth** | | | | | |
|  | Paucigravidae | 533, 38.0 (2.5) | 532, 38.2 (2.4) | -0.15 (-0.44, 0.15) | 0.327 |
|  | Multigravidae | 338, 38.2 (2.2) | 332, 38.5 (2.0) | -0.28 (-0.59, 0.04) | 0.083 |
|  | All gravidae | 871, 38.1 (2.4) | 864, 38.3 (2.3) | -0.20 (-0.41, 0.02) | 0.076 |
| **Birthweight for gestational age Z-score** | | | | | |
|  | Paucigravidae | 501, 0.0 (0.9) | 505, 0.1 (0.9) | -0.06 (-0.18, 0.05) | 0.280 |
|  | Multigravidae | 314, 0.3 (1.0) | 309, 0.3 (0.9) | 0.02 (-0.13, 0.17) | 0.766 |
|  | All gravidae | 815, 0.1 (0.9) | 814, 0.2 (1.0) | -0.03 (-0.12, 0.06) | 0.540 |
