# Supplementary material for: Scheduled Intermittent Screening with Rapid Diagnostic Tests and Treatment with Dihydroartemisinin-Piperaquine versus Intermittent Preventive Therapy with Sulfadoxine-Pyrimethamine for Malaria in Pregnancy in Malawi: An Open-Label Randomized Controlled Trial
Source: PLoS Med. 2016 Sep 13;13(9):e1002124. doi: 10.1371/journal.pmed.1002124 (PMC5021271; doi:10.1371/journal.pmed.1002124)
Supplement: S9 Table — (DOCX) [file pmed.1002124.s016.docx]

| **S9 Table: Adherence and tolerance of study drugs and regimen by pregnant women** | | | |
| --- | --- | --- | --- |
| **Outcome** | | **ISTp-DP** | **IPTp-SP** |
| **Number of women who did not tolerate DP or SP at least once (%)^a^** | | | |
|  | Paucigravidae | 1/351 (0.3) | 0/569 (0.0) |
|  | Multigravidae | 1/122 (0.8) | 0/352 (0.0) |
|  | All gravidae | 2/473 (0.4) | 0/921 (0.0) |
| 1. Defined as vomiting of primary and repeat dose, requiring alternative treatment with AL or parenteral antimalarials. All doses in both arms were given under direct supervision by study staff, either in the clinic or at home. | | | |
