# Supplementary material for: Scheduled Intermittent Screening with Rapid Diagnostic Tests and Treatment with Dihydroartemisinin-Piperaquine versus Intermittent Preventive Therapy with Sulfadoxine-Pyrimethamine for Malaria in Pregnancy in Malawi: An Open-Label Randomized Controlled Trial
Source: PLoS Med. 2016 Sep 13;13(9):e1002124. doi: 10.1371/journal.pmed.1002124 (PMC5021271; doi:10.1371/journal.pmed.1002124)
Supplement: S10 Table — (DOCX) [file pmed.1002124.s017.docx]

**S10 Table: Foetal loss, perinatal and infant death among DP recipients and non-recipients in the ISTp-DP arm, compared with the IPTp-SP arm**

|  | | **# with (at least one) event / # followed (%), or**  **# events, # with (at least one) event/ # followed (%)** | | | | **Risk Ratio (95% CI), p-value** | | | |
| --- | --- | --- | --- | --- | --- | --- | --- | --- | --- |
| **Outcome** | | **ISTp-DP:**  **DP-non-recipients** | **ISTp-DP:**  **DP-recipients** | **ISTp-DP**  **total** | **IPTP-SP** | **ISTp-DP total**  **vs**  **IPTp-SP** | **ISTp-DP non-DP- recipients vs**  **DP-recipients** | **ISTp-DP non-DP-recipients vs**  **IPTp-SP arm** | **ISTp-DP DP-recipients vs**  **IPTp-SP arm** |
| **Maternal SAEs (cumulative risk [% at least 1 event])** | | | | | |  |  |  |  |
|  | Paucigravidae | 12/220 (5.5) | 15/351 (4.3) | 27/571 (4.7) | 29/569 (5.1) | 0.93 (0.56, 1.55), 0.7738 | 1.28 (0.61, 2.68), 0.5182 | 1.07 (0.56, 2.06), 0.8390 | 0.84 (0.46, 1.54), 0.5708 |
|  | Multigravidae | 12/230 (5.2) | 4/122 (3.3) | 16/352 (4.5) | 16/352 (4.5) | 1.00 (0.51, 1.97), 1.0000 | 1.59 (0.52, 4.83), 0.4121 | 1.15 (0.55, 2.38), 0.7112 | 0.72 (0.25, 2.12), 0.5519 |
|  | All gravidae | 24/450 (5.3) | 19/473 (4.0) | 43/923 (4.7) | 45/921 (4.9) | 0.95 (0.63, 1.43), 0.8190 | 1.33 (0.74, 2.39), 0.3446 | 1.09 (0.67, 1.77), 0.7219 | 0.82 (0.49, 1.39), 0.4644 |
| **Maternal deaths** | | |  |  |  |  |  |  |  |
|  | Paucigravidae | 0/220 (0.0) | 0/351 (0.0) | 0/571 (0.0) | 1/569 (0.2) | CBE^a^ | CBE^a^ | CBE^a^ | CBE^a^ |
|  | Multigravidae | 0/230 (0.0) | 0/122 (0.0) | 0/352 (0.0) | 1/352 (0.3) | CBE^a^ | CBE^a^ | CBE^a^ | CBE^a^ |
|  | All gravidae | 0/450 (0.0) | 0/473 (0.0) | 0/923 (0.0) | 2/921 (0.2) | CBE^a^ | CBE^a^ | CBE^a^ | CBE^a^ |
| **Congenital malformations** | | |  |  |  |  |  |  |  |
|  | Paucigravidae | 3/195 (1.5) | 3/333 (0.9) | 6/528 (1.1) | 7/529 (1.3) | 0.89 (0.29, 2.54), 0.7828 | 1.71 (0.35, 8.38), 0.5047 | 1.16 (0.30, 4.45), 0.8258 | 0.68 (0.18, 2.61), 0.5729 |
|  | Multigravidae | 3/217 (1.4) | 1/117 (0.9) | 4/334 (1.2) | 2/330 (0.6) | 1.98 (0.36, 10.72), 0.4206 | 1.62 (0.17, 15.38), 0.6723 | 2.28 (0.38, 13.54), 0.3506 | 1.41 (0.13, 15.41), 0.7772 |
|  | All gravidae | 6/412 (1.5) | 4/450 (0.9) | 10/862 (1.2) | 9/859 (1.0) | 1.11 (0.45, 2.71), 0.8235 | 1.64 (0.47, 5.76), 0.4371 | 1.39 (0.50, 3.88), 0.5278 | 0.85 (0.26, 2.74), 0.7831 |
| **Foetal Loss** | |  |  |  |  |  |  |  |  |
|  | Paucigravidae | 7/201 (3.5) | 8/338 (2.4) | 15/539 (2.8) | 6/533 (1.1) | 2.47 (0.97, 6.32), 0.0589 | 1.47 (0.54, 4.00) 0.4487 | 3.09 (1.05, 9.09), 0.0401 | 2.10 (0.74, 6.01), 0.1653 |
|  | Multigravidae | 6/220 (2.7) | 2/118 (1.7) | 8/338 (2.4) | 5/333 (1.5) | 1.58 (0.52, 4.77), 0.4204 | 1.61 (0.33, 7.85) 0.5563 | 1.82 (0.56, 5.88), 0.3193 | 1.13 (0.22, 5.74), 0.8839 |
|  | All gravidae | 13/421 (3.1) | 10/456 (2.2) | 23/877 (2.6) | 11/866 (1.3) | 2.06 (1.01, 4.21), 0.0461 | 1.41 (0.62, 3.18), 0.4098 | 2.43 (1.10, 5.38), 0.0284 | 1.73 (0.74, 4.03), 0.2073 |
| **Miscarriage** | |  |  |  |  |  |  |  |  |
|  | Paucigravidae | 3/201 (1.5) | 2/338 (0.6) | 5/539 (0.9) | 2/533 (0.4) | 2.47 (0.48, 12.69), 0.2681 | 2.52 (0.43, 14.97), 0.3085 | 3.98 (0.67, 23.63), 0.1288 | 1.58 (0.22, 11.14), 0.6480 |
|  | Multigravidae | 2/220 (0.9) | 0/118 (0) | 2/338 (0.6) | 1/333 (0.3) | 1.97 (0.18, 21.63), 0.5790 | CBE^a^ | 3.03 (0.28, 33.18), 0.3646 | CBE^a^ |
|  | All gravidae | 5/421 (1.2) | 2/456 (0.4) | 7/877 (0.8) | 3/866 (0.3) | 2.30 (0.60, 8.88), 0.2253 | 2.71 (0.53, 13.88), 0.2323 | 3.43 (0.82, 14.28), 0.0905 | 1.27 (0.21, 7.55), 0.7957 |
| **Stillbirth** | |  |  |  |  |  |  |  |  |
|  | Paucigravidae | 4/198 (2.0) | 6/336 (1.8) | 10/534 (1.9) | 4/531 (0.8) | 2.49 (0.78, 7.88), 0.1217 | 1.13 (0.32, 3.96), 0.8470 | 2.68 (0.68, 10.62), 0.1601 | 2.37 (0.67, 8.34), 0.1786 |
|  | Multigravidae | 4/218 (1.8) | 2/118 (1.7) | 6/336 (1.8) | 4/332 (1.2) | 1.48 (0.42, 5.20), 0.5392 | 1.08 (0.20, 5.82), 0.9264 | 1.52 (0.38, 6.03), 0.5489 | 1.41 (0.26, 7.58), 0.6913 |
|  | All gravidae | 8/416 (1.9) | 8/454 (1.8) | 16/870 (1.8) | 8/863 (0.9) | 1.98 (0.85, 4.61), 0.1114 | 1.09 (0.41, 2.88), 0.8599 | 2.07 (0.78, 5.49), 0.1416 | 1.90 (0.72, 5.03), 0.1959 |
| **Infant SAEs (cumulative risk [% at least 1 event])** | | | | | | |  |  |  |
|  | Paucigravidae | 8/220 (3.6) | 21/351 (6.0) | 29/571 (5.1) | 30/539 (5.6) | 0.96 (0.59, 1.58), 0.8827 | 0.61 (0.27, 1.35), 0.2139 | 0.69 (0.32, 1.48), 0.3358 | 1.13 (0.66, 1.95), 0.6473 |
|  | Multigravidae | 6/224 (2.7) | 4/122 (3.3) | 10/352 (2.8) | 8/352 (2.3) | 1.25 (0.50, 3.13), 0.6330 | 0.80 (0.23, 2.77), 0.7188 | 1.15 (0.40, 3.26), 0. 7959 | 1.44 (0.44, 4.71), 0.5422 |
|  | All gravidae | 14/450 (3.1) | 25/473 (5.3) | 39/923 (4.2) | 38/921 (4.1) | 1.02 (0.66, 1.59), 0.9150 | 0.59 (0.31, 1.12), 0.1007 | 0.75 (0.41, 1.38), 0.3556 | 1.28 (0.78, 2.10), 0.3238 |
| **Perinatal death** | |  |  |  |  |  |  |  |  |
|  | Paucigravidae | 7/196 (3.6) | 11/333 (3.3) | 18/529 (3.4) | 12/528 (2.3) | 1.50 (0.73, 3.08), 0.2722 | 1.08 (0.43, 2.74), 0.8695 | 1.57 (0.63, 3.93), 0.3343 | 1.45 (0.65, 3.26), 0.3635 |
|  | Multigravidae | 6/216 (2.8) | 2/118 (1.7) | 8/334 (2.4) | 4/330 (1.2) | 1.98 (0.60, 6.50), 0.2622 | 1.64 (0.34, 7.99), 0.5411 | 2.29 (0.65, 8.03), 0.1947 | 1.40 (0.26, 7.54), 0.6964 |
|  | All gravidae | 13/412 (3.2) | 13/451 (2.9) | 26/863 (3.0) | 16/858 (1.9) | 1.62 (0.87, 2.99), 0.1267 | 1.09 (0.51, 2.33), 0.8149 | 1.69 (0.82, 3.48), 0.1536 | 1.55 (0.75, 3.18), 0.2377 |
| **Neonatal death (by 4 weeks)** | | |  |  |  |  |  |  |  |
|  | Paucigravidae | 4/192 (2.1) | 6/327 (1.8) | 10/519 (1.9) | 8/524 (1.5) | 1.26 (0.50, 3.17), 0.6207 | 1.14 (0.32, 3.97), 0.8425 | 1.36 (0.42, 4.48), 0.6083 | 1.20 (0.42, 3.43), 0.7313 |
|  | Multigravidae | 2/212 (0.9) | 0/116 (0) | 2/328 (0.6) | 0/326 (0) | CBE^a^ | CBE^a^ | CBE^a^ | CBE ^a^ |
|  | All gravidae | 6/404 (1.5) | 6/443 (1.4) | 12/847 (1.4) | 8/850 (0.9) | 1.51 (0.62, 3.66), 0.3675 | 1.10 (0.36, 3.37), 0.8723 | 1.58 (0.55, 4.52), 0.3954 | 1.44 (0.50, 4.12), 0.4978 |
| **Infant death (by 6 to 8 weeks)** | | |  |  |  |  |  |  |  |
|  | Paucigravidae | 4/213 (1.9) | 8/343 (2.3) | 12/556 (2.2) | 9/563 (1.6) | 1.35 (0.57, 3.18), 0.4920 | 0.81 (0.25, 2.64), 0.7207 | 1.17 (0.37, 3.77), 0.7867 | 1.46 (0.57, 3.75), 0.4323 |
|  | Multigravidae | 2/224 (0.9) | 0/120 (0) | 2/344 (0.6) | 1/347 (0.3) | 2.02 (0.18, 22.15), 0.5659 | CBE^a^ | 3.10 (0.28, 33.97), 0.3547 | CBE, 0.5561 |
|  | All gravidae | 6/437 (1.4) | 8/463 (1.7) | 14/900 (1.6) | 10/910 (1.1) | 1.42 (0.63, 3.17), 0.3982 | 0.79 (0.28, 2.27), 0.6680 | 1.25 (0.46, 3.42), 0.6643 | 1.57 (0.62, 3.96), 0.3365 |
| **Foetal loss or infant death** | | |  |  |  |  |  |  |  |
|  | Paucigravidae | 11/220 (5.0) | 16/351 (4.6) | 27/571 (4.7) | 15/569 (2.6) | 1.79 (0.96, 3.34), 0.0649 | 1.10 (0.52, 2.32), 0.8088 | 1.90 (0.88, 4.06), 0.0998 | 1.73 (0.87, 3.45), 0.1207 |
|  | Multigravidae | 8/230 (3.5) | 2/122 (1.6) | 10/352 (2.8) | 6/352 (1.7) | 1.67 (0.61, 4.54), 0.3173 | 2.12 (0.46, 9.84), 0.3364 | 2.04 (0.72, 5.80), 0.1811 | 0.96 (0.20, 4.70), 0.9616 |
|  | All gravidae | 19/450 (4.2) | 18/473 (3.8) | 37/923 (4.0) | 21/921 (2.3) | 1.76 (1.04, 2.98), 0.0361 | 1.11 (0.59, 2.09), 0.7471 | 1.85 (1.01, 3.41), 0.0478 | 1.67 (0.90, 3.10), 0.1052 |
| CBE=RR and 95% CI cannot be estimated.  The comparisons between the ISTp-DP arm and the IPTp-SP arm were pre-specified. The comparisons between DP recipients and non-recipients in the ISTp-DP arm were post-hoc. | | | | | | | | | |
