# Supplementary material for: Scheduled Intermittent Screening with Rapid Diagnostic Tests and Treatment with Dihydroartemisinin-Piperaquine versus Intermittent Preventive Therapy with Sulfadoxine-Pyrimethamine for Malaria in Pregnancy in Malawi: An Open-Label Randomized Controlled Trial
Source: PLoS Med. 2016 Sep 13;13(9):e1002124. doi: 10.1371/journal.pmed.1002124 (PMC5021271; doi:10.1371/journal.pmed.1002124)
Supplement: S1 Text — (DOCX) [file pmed.1002124.s018.docx]

# Supplementary methods

## Sample size calculations

The study was designed to provide 90% power to detect a 25% reduction in the composite adverse birth outcome among paucigravidae from 40.3% in the IPTp-SP arm to 30.2% in the ISTp-DP arm. The target sample size was 1155 paucigravidae, (491 per arm after allowing for 15% loss to follow-up) (alpha=0.05). Multigravidae were recruited alongside until the target sample size in paucigravidae was reached. The original target sample size for enrolment of multigravidae was 500 and based on the detection of a 50% reduction in malaria at delivery from an estimated 20.5% to 10.25% with 90% power and allowing for 15% loss to follow-up. However, during the conduct of the study it become clear that this sample size would be reached several months before the target number of paucigravidae was achieved. To avoid seasonal bias in recruitment between the two gravidae strata and allow for pooling of gravidae strata in the analysis, an amendment was approved to allow continuation of recruitment of all eligible multigravidae until the target sample size in paucigravidae was reached. Thus the total number of multigravidae enrolled would depend on the recruitment rate among paucigravidae and was estimated to range between 577 and 770, providing between 86% and 94% power to detect a 50% difference in malaria at delivery. Recruitment was stopped on March 18, 2015, when the sample size in the paucigravidae was reached.

## Definitions of morbidity endpoints

### Birthweight data

The aim of the study was to measure birthweight within 24 hours after birth. Birth weights taken 24-48h hours (n=72, 4.4%), and 48-168 hours after delivery (n=5, 0.3%) were corrected for the physiological fall in birth weight in breastfed infants occurring in the first days following delivery [1, 2] by a factor +2% and +4%, respectively to obtain the estimated weight at birth [3, 4]. All analyses used corrected birthweight unless indicated otherwise. Low birth weight was defined as <2,500 gr.

### Gestational age and preterm

A dating ultrasound scan (Sonosite 180-plus portable ultrasound system, Sonosite, USA), was performed by the study clinician (MM) and a trained study nurse within a week of enrolment using biparietal diameter, abdominal circumference and femur length to estimate gestational age. If more than one gestational age measurement was available we used estimates in the following order of preference: ultrasound, neonatal clinical exam within 96 hours of delivery (modified Ballard score), last menstrual period (if known), and fundal height at enrolment. Preterm was defined as a gestational age of less than 37 completed weeks.

### Small for gestational age (SGA)

SGA was defined as birthweight below the tenth percentile of an external reference population for a given gestational age and sex. Because ultrasound-based fetal growth charts were not available for Malawian populations, an ultrasound-based fetal growth chart representative for a rural Tanzanian population was used, which also allowed the calculation of Z-scores [5]. The Tanzanian reference population was described in the statistical analysis plan (S2 Text) and replaced the original fetal growth charts based on an urban population from Kinshasa, Democratic Republic of Congo (DRC), described in the study protocol (S3 Text). This was done because the latter was smaller (N=144) and included women suffering from malaria, malnutrition and obstetrical complications [2].

### Congenital malaria

Any asexual malaria parasitaemia detected by microscopy, RDT or PCR in cord blood or in the peripheral blood within 7 days of birth.

## Laboratory methods

### Hemoglobin concentrations

Hemoglobin concentrations were measured at point of care using the Hb 301+ system from Hemocue. Anemia was defined as a hemoglobin level <11 g/dL, and moderate-to-severe anemia as <9 g/dL (S2 Text). The original definition for moderate-to-severe anemia defined in the protocol (S3 Text) and trial registration (S4 Text) was Hb<8 g/dL, but subsequent analysis of observational studies of malaria in pregnancy completed just prior to the trial showed that this outcome was very rare among pregnant women in this site (~1%) and the cut-off of 9 g/dL was used instead to provide more power to this endpoint.

### Malaria microscopy

Malaria smears were not used for point of care and read several weeks after they were taken in a central laboratory at the College of Medicine in Blantyre. All smears were read in duplicate, and discrepancies (positive vs negative) resolved by using a third reader, who was blinded to the initial results. Plasmodium infection was defined as the presence of asexual Plasmodium parasites (any species) in a thick blood smear. Parasite densities were counted against 300 white blood cells and expressed per 8,000 parasites per uL. Smears were declared negative if no parasites were detected after examining 200 high power fields.

### Molecular methods and real-time PCR

Dried blood spots (DBSs) for PCR assays were stored on Whatman’s #3 filter paper in individual plastic bags with desiccant. From these spots, genomic DNA (gDNA) was extracted using a Chelex-100 protocol on three 5mm punches from each DBS. gDNA specimens were tested in duplicate in a TaqMan real-time PCR assay targeting the *P. falciparum* lactate dehydrogenase gene (*pfldh*); the reaction mix was identical to prior studies, except the reaction volume was reduced to 12uL and 2uL of template were added to each. Samples were tested on 384-well reaction plates using a BioRad CFX384 Touch machine, threshold lines were set manually for each reaction plate, and quantitation cycle (Cq) values were computed using the baseline subtracted curve fit setting. Each reaction plate included a series of 10 standards in duplicate of *P. falciparum* strain 3D7 gDNA in concentrations from 0.1ng/uL to 5x10-6ng/uL. The Cq values of these controls were used to compute standard curves that were used to estimate parasite gDNA quantity in the clinical specimens. Each plate also included four negative controls with molecular-grade water in place of DNA, and all plates were prepared in a PCR hood using filtered pipet tips. The personnel that performed the molecular testing were masked to the patient's allocated study arm.

### Genotyping for parasite resistance

We pooled genomic DNA from samples collected at enrollment that were positive for *P. falciparum* in a real-time PCR assay. Parasite-positive DNA samples were pooled by study site and treatment allocation arm with equal volumes, and the numbers of parasitemias in each pool varied between 76 and 120. From these six pools, we amplified *P. falciparum* *dhfr* and *dhps* genes in nested PCR assays as previously described [6]. Amplicons were confirmed by gel electrophoresis, and prepared as barcoded sequencing libraries using the NEBNext Fast DNA Fragmentation and Library Prep Set for Ion Torrent (New England Biolabs, Ipswich, MA, USA) and the Ion Xpress Barcode Adaptors (Thermo Fisher Scientific, USA) using standard protocols. Barcoded libraries were mixed in equimolar amounts by gene target and sequenced on an Ion Torrent PGM platform using 318 chips. Reads were analyzed in Galaxy (usegalaxy.org) [7-9]: reads were first aligned to 3D7 reference sequences for either *dhfr* (GenBank XM_001351443) or *dhps* (Z30654) using Bowtie2 and then variants at each position were quantified using MPileup in order to generate vcf files; for quality filtering of allele frequencies, we allowed reads of any quality to be mapped to reference sequences, but analyzed within those reads only bases with quality scores >q33 for *dhfr* or >q29 for *dhps*, consistent with our prior Ion Torrent pooled sequencing projects [10], which enforces stringent quality while limiting false-discovery. At the loci of interest in *dhfr* and *dhps*, the mutant allele frequency was defined as the proportion of reads at that locus that harbored the nucleotide substitution. Overall and mutant read counts were summed between treatment arms to compute allele frequencies by study site, and summed to compute aggregate allele frequencies in the baseline *P. falciparum* infections.

## Nested sub studies

As indicated in the original trial protocol (S3 Text) and registration (S4 Text) [11], the trial included several secondary endpoints which were collected as part of nested studies that have or will be reported elsewhere. These included immunological studies of the concentration of antibodies known to be associated with protection against malaria in pregnancy, and specifically of those recognizing variant surface antigens on P. falciparum infected erythrocytes that block parasite adhesion to chondroitin sulphate A (S4 Text, outcome 25); economic sub-studies (S4 Text outcome 26 to 28); and studies of the acceptability, feasibility, implementability and scale up of ISTp (S4 Text, outcomes 29.1 to 29.5) [11]. The results of the latter were partly published elsewhere [12].

# References

1. Noel-Weiss J, Courant G, Woodend AK. Physiological weight loss in the breastfed neonate: a systematic review. Open medicine : a peer-reviewed, independent, open-access journal. 2008;2(4):e99-e110. PubMed PMID: 21602959; PubMed Central PMCID: PMC3091615.

2. Flaherman VJ, Kuzniewicz MW, Li S, Walsh E, McCulloch CE, Newman TB. First-day weight loss predicts eventual weight nadir for breastfeeding newborns. Archives of disease in childhood Fetal and neonatal edition. 2013;98(6):F488-92. doi: 10.1136/archdischild-2012-303076. PubMed PMID: 23864443.

3. Greenwood BM, Greenwood AM, Snow RW, Byass P, Bennett S, Hatib-N'Jie AB. The effects of malaria chemoprophylaxis given by traditional birth attendants on the course and outcome of pregnancy. Transactions of the Royal Society of Tropical Medicine and Hygiene. 1989;83(5):589-94. PubMed PMID: 2617619.

4. D'Alessandro U, Langerock P, Bennett S, Francis N, Cham K, Greenwood BM. The impact of a national impregnated bed net programme on the outcome of pregnancy in primigravidae in The Gambia. Transactions of the Royal Society of Tropical Medicine and Hygiene. 1996;90(5):487-92. PubMed PMID: 8944251.

5. Schmiegelow C, Scheike T, Oesterholt M, Minja D, Pehrson C, Magistrado P, et al. Development of a fetal weight chart using serial trans-abdominal ultrasound in an East African population: a longitudinal observational study. PloS one. 2012;7(9):e44773. doi: 10.1371/journal.pone.0044773. PubMed PMID: 23028617; PubMed Central PMCID: PMC3448622.

6. Taylor SM, Antonia A, Feng G, Mwapasa V, Chaluluka E, Molyneux M, et al. Adaptive evolution and fixation of drug-resistant Plasmodium falciparum genotypes in pregnancy-associated malaria: 9-year results from the QuEERPAM study. Infection, genetics and evolution : journal of molecular epidemiology and evolutionary genetics in infectious diseases. 2012;12(2):282-90. Epub 2011/11/29. doi: 10.1016/j.meegid.2011.11.006. PubMed PMID: 22119749; PubMed Central PMCID: PMC3293939.

7. Giardine B, Riemer C, Hardison RC, Burhans R, Elnitski L, Shah P, et al. Galaxy: a platform for interactive large-scale genome analysis. Genome Res. 2005;15(10):1451-5. doi: 10.1101/gr.4086505. PubMed PMID: 16169926; PubMed Central PMCID: PMCPMC1240089.

8. Blankenberg D, Von Kuster G, Coraor N, Ananda G, Lazarus R, Mangan M, et al. Galaxy: a web-based genome analysis tool for experimentalists. Curr Protoc Mol Biol. 2010;Chapter 19:Unit 19 0 1-21. doi: 10.1002/0471142727.mb1910s89. PubMed PMID: 20069535; PubMed Central PMCID: PMCPMC4264107.

9. Goecks J, Nekrutenko A, Taylor J, Galaxy T. Galaxy: a comprehensive approach for supporting accessible, reproducible, and transparent computational research in the life sciences. Genome Biol. 2010;11(8):R86. doi: 10.1186/gb-2010-11-8-r86. PubMed PMID: 20738864; PubMed Central PMCID: PMCPMC2945788.

10. Taylor SM, Parobek CM, DeConti DK, Kayentao K, Coulibaly SO, Greenwood BM, et al. Absence of putative artemisinin resistance mutations among Plasmodium falciparum in Sub-Saharan Africa: a molecular epidemiologic study. The Journal of infectious diseases. 2015;211(5):680-8. doi: 10.1093/infdis/jiu467. PubMed PMID: 25180240; PubMed Central PMCID: PMCPMC4402372.

11. ter Kuile FO. Trial registration ISRCTN69800930: Scheduled screening versus preventive treatment for the control of malaria in pregnancy in Malawi: a randomized controlled trial 2011. Available from: <http://www.isrctn.com/ISRCTN69800930>.

12. Pell C, Menaca A, Afrah NA, Manda-Taylor L, Chatio S, Were F, et al. Prevention and management of malaria during pregnancy: findings from a comparative qualitative study in Ghana, Kenya and Malawi. Malar J. 2013;12:427. doi: 10.1186/1475-2875-12-427. PubMed PMID: 24257105; PubMed Central PMCID: PMCPMC3874601.
