# Supplementary material for: Scheduled Intermittent Screening with Rapid Diagnostic Tests and Treatment with Dihydroartemisinin-Piperaquine versus Intermittent Preventive Therapy with Sulfadoxine-Pyrimethamine for Malaria in Pregnancy in Malawi: An Open-Label Randomized Controlled Trial
Source: PLoS Med. 2016 Sep 13;13(9):e1002124. doi: 10.1371/journal.pmed.1002124 (PMC5021271; doi:10.1371/journal.pmed.1002124)
Supplement: S2 Text — (PDF) [file pmed.1002124.s019.pdf]

STATISTICAL ANALYSIS PLAN

Trial Full title: Scheduled screening versus preventive treatment for the control of malaria in pregnancy in Malawi: a randomized controlled trial

Short Title: ISTp-Malawi

Study Identifiers:			
NHSRC<br>#916	LSTM REC, UK:<br>10.74	ISRCTN<br>69800930	Protocol Version<br>4.01: 01 July 2012

Chief Investigator: Prof Feiko ter Kuile (email: feiko.terkuile@lstmed.ac.uk)

Site Co-Principal Investigators: Associate Prof. Victor Mwapasa (vmwapasa69@gmail.com) and Associate Prof.Linda Kalilani-Phiri (email: lkalilani@medcol.mw

Co-Investigators: Mwayiwawo Madanitsa, Stephen Rogerson, Silke Lutzelschwab, Kara Hanson, Brian Faragher, Arthur Kang'ombe, Doreen Ali, Annemieke van Eijk, Steve Taylor, Steve Meshnick

Trial statisticians: Prof Duolao Wang and Prof Brian Faragher

SAP authors: Arthur Kang'ombe, Mwayiwawo Madanitsa , Feiko ter Kuile, Duolao Wang

Funder: European and Developing Countries Clinical Trials Partnership (EDCTP)

Sponsor: Liverpool School of Tropical Medicine (LSTM); Pembroke Place, Liverpool L3 5QA, UK
Phone: +44 0151 7053212; Email: S.Roberts@liverpool.ac.uk

SAP version History:

Version Date	SAP Version #	Details of Changes	Signature Chief Investigator
06 Oct, 2014	1.1		
09 Dec, 2014	1.2		
27 Feb 2015	1.3		
03 Mar 2015	1.4		
20 Mar 2015	1.5		
11 Apr 2015	1.6		
21 Apr 2015	1.7	Definition of per protocol	

Confidentiality Statement: This document contains confidential information that must not be disclosed to anyone other than the sponsor, the investigator team, host institution, relevant ethics committee and regulatory authorities

1. Signatures

	Signature	Date
Prof Duolao Wang (Trial Statistician)	Duolao Wang	01 May 2015
Prof Feiko ter Kuile (Chief Investigator)	[illegible]	21 Apr 2015
Paul Milligan (Statistician: DSMB)	P. Milligan	28 Apr 2015

....

Table of Contents

1. Signatures....2
2. List of abbreviations....5
3. Introduction....6
3.1. Overview design....6
3.2. Sample size....6
17.2 Randomization and allocation....6
4. Purpose of the analysis plan....7
5. Definitions....7
5.1. Malaria infection endpoint definitions....7
5.2. Morbidity endpoint definitions....9
5.3. Definitions for other Endpoints....12
5.4. Definitions for other variables....13
6. Study Endpoints....14
6.1. Primary outcome....14
6.1.1. First and second pregnancies....14
6.1.2. Multigravidae....14
6.2. Secondary efficacy outcomes....14
6.2.1. Antenatal....14
6.2.2. At delivery....14
6.2.3. After delivery....15
6.3. Secondary safety outcomes....16
6.4. Tolerability outcomes....16
7. Analytical population....16
7.1. Full analysis population for modified Intention to treat analysis (ITT)....16
7.2. Per protocol Population (PP);....16
7.3. Safety Population....17
8. General analytical approach....17
8.1. Reporting guidelines....17
8.2. Pooling of data from the 3 sites....17
8.3. Pre-scheduled stopping of study participants and use of data....17
8.4. Missing data....17
8.4.1. Endpoints....17
8.4.2. Covariates....17
8.5. Stratification by gravidity....18
8.6. Linear regression analysis for adjusted analysis of dichotomous outcomes....18
8.6.1. Risk ratios or odds ratios?....18
8.6.2. Log binomial regression and alternative strategies in case of non-convergence....18
8.7. Reporting conventions....19
8.7.1. Descriptive statistics....19
8.7.2. Measures of associations and P-value reporting....19
9. Participant disposition and Flow chart....19
10. Baseline data summaries....20
10.1. Demographic, clinical and laboratory measures....20
10.2. Measures of Social Economic Status (SES) Asset index.....20
11. Efficacy analyses....20
11.1. Measure of associations....20
11.1.1. Binary outcomes....20

11.1.2. Continuous outcomes 20
11.2. Forest plots of efficacy parameters 21
11.3. Primary efficacy endpoint analysis 21
11.3.1. Crude and adjusted effect estimates 21
11.3.2. Adjustment for baseline independent variables in the multiple regression models.... 21
11.3.3. Variable specification 22
11.4. Secondary efficacy analysis of primary outcomes 22
11.4.1. Overall pooled effect (all gravidae) 22
11.4.2. Subgroup analysis 22
11.4.3. Sensitivity analysis 23
11.5. Secondary efficacy outcomes 23
11.5.1. Outcomes at delivery 23
11.5.2. Count data outcomes 23
12. Safety analysis 24
13. Other analysis 24
13.1. Number of intervention visits 24
13.2. Compliance with SP and DHA-PQ. 24
13.3. Treatment response 24
14. References 25
15. Tables and Figures 26
15.1. List of tables 26
Patient disposition (Flow Chart) 28
15.2. Versioning manuscript 29
15.2.1. Word document 29
15.2.2. Endnote 29
16. Appendix 1: CONSORT checklist of information to include when reporting a trial* 30

2. List of abbreviations

AE	Adverse event
AL	Artemether-lumefantrine
AQ-AS	Artesunate-amodiaquine
CoM	University of Malawi College of Medicine
CRF	Case record form
DHA-PQ	Dihydroartemisinin-piperaquine
EPI	Expanded programme on immunisation
FGD	Focus group discussion
G1-G2	primigravidae and secundigravidae
G3+	Multigravidae
Hb	Haemoglobin
HIV	Human immunodeficiency virus
HRP-2	Histidine-rich protein two
IPTp	Intermittent preventive treatment in pregnancy
IPTp-SP	Intermittent preventive treatment in pregnancy using sulphadoxine-pyrimethamine
ISTp	Intermittent screening and treatment in pregnancy
ISTp-DP	Intermittent screening and treatment in pregnancy, screening and treating malaria cases with dihydroartemisinin-piperaquine
ITN	Insecticide treated (bed) net
LMP	Last menstrual period
LSTM	Liverpool School of Tropical Medicine
MiP	Malaria in Pregnancy (Consortium)
MQ-AS	Mefloquine-artesunate
PI	Principal Investigator
PCR	Polymerase chain reaction
P. falciparum	*Plasmodium falciparum*
PfEMP1	*P. falciparum* erythrocyte membrane protein 1
pLDH	Plasmodium lactate dehydrogenase
RDT	Rapid diagnostic test (for malaria)
SAE	Serious adverse event
SP	Sulphadoxine-pyrimethamine
SUSAR	Suspected unexpected adverse reaction
VSA	Variant surface antigens
WHO	World Health Organization

3. Introduction

3.1. Overview design

This is a two-arm randomised controlled superiority trial to compare the efficacy of scheduled intermittent screening with malaria rapid diagnostic tests (RDTs) and treatment of RDT-positive women with dihydroartemisinin-piperaquine (ISTp-DP) with intermittent preventive treatment with sulphadoxine-pyrimethamine (IPTp-SP) in the second and third trimesters on adverse birth outcome and malaria infection at term among HIV-negative women protected by insecticide–treated bed nets.

This is a stratified study (1st stratum: primigravidae and secundigravidae [G1-G2]. 2nd stratum: multigravidae [G3+]). In the 1st stratum (G1-G2), it is designed to detect a reduction in the composite adverse birth outcomes (Small for Gestation Age (SGA), low birth weight and pre-term birth). In the 2nd stratum (G3+), the trial is designed to detect a reduction in past or present placental malaria infection at delivery.

3.2. Sample size

In the first stratum, 491 participants in each arm or 982 in total was required to give 90% power to detect a 25% reduction in the composite adverse birth outcome from 40.3 to 30.2% at 5% significance level using a two sided test. To allow for a 15% loss to follow-up, 1155 women in their first and second pregnancies were to be recruited. In the 2nd stratum, 213 women per arm or 426 in total was required to give 80% power of detecting a 50% reduction in past or present placental malaria infection from 20.5% to 10.25% at 5% significance level using a two sided test. To allow for a 15% loss to follow-up at least 500 multigravidae were going to be recruited. Recruitment of eligible multigravidae occurred in parallel to primi and secundgravidae without restricting recruitment of multigravidae, and was stopped when the required sample size of 1155 primi,- and secundigravidae had been reached. Therefore the total number of multigravidae enrolled exceeded 500.

17.2 Randomization and allocation

Two randomization sequences were computer-generated by the study statistician at Liverpool School of Tropical Medicine, one for women in their first and second pregnancies and another for multigravidae. The method used for each was block randomization, stratified by study site to ensure an equal proportion of participants in each intervention group from each site. The length of each block varied to ensure that the allocation concealment was fully maintained and allocation well distributed over the seasons. The allocation ratio for the two study arms was be 1:1.

Recruitment was ‘competitive’ between study sites. A sufficient number of randomization codes was generated for each site to allow for this. The randomization sequences was sent to the study sites in Malawi and local investigators prepared opaque envelopes, numbered sequentially, with the allocated group code and details for that number inside. For each newly enrolled participant, an envelope from the correct sequence (according to gravidity group) was opened sequentially to identify the group that they were allocated.

4. Purpose of the analysis plan

The purpose of this document is to outline the statistical analysis plan for this study which was primarily meant to compare the efficacy of scheduled intermittent screening with malaria rapid diagnostic tests (RDTs) and treatment of RDT-positive women with dihydroartemisinin-piperaquine (ISTp-DP) with intermittent preventive treatment with sulphadoxine-pyrimethamine (IPTp-SP) in the second and third trimesters on adverse birth outcome and malaria infection at term among HIV-negative women protected by insecticide–treated bed nets.

The SAP is based on the last version of the amended protocol (v4.1 01Jul12) and has been finalised before the data cleaning was finalised, the dataset was locked and before any data analysis was started. One interim analysis was planned but not conducted following discussions with the DSMB.

5. Definitions

5.1. Malaria infection endpoint definitions

See also Table 1, page 9: *Definition malaria infection*.

For all the below definition of malaria infection reference is made to any species (Plasmodium falciparum, vivax, malariae, ovale etc)

1. Placental malaria infection, (yes/no): Plasmodium malaria infection detected in the maternal blood/maternal site of the placenta by either
    a. Placental impression smear microscopy (standard microscopy)
    b. RDT (pLDH or HRP2 or both)
    c. Placental malaria by histology (active or past)
    d. PCR
    - Considers maternal placental blood only (i.e. ignores information from the peripheral maternal blood)
2. Any placental malaria infection by placental histology, (yes/no)
    a. Past infection (pigment in fibrin detected in the absence of asexual parasites)
    b. Active infection (acute or chronic) (asexual parasite present)
    - Considers maternal placental tissue only and includes past (asexual stage cleared) as well as active infections (asexual stage present)
3. Active Placental malaria infection by placental Histology, (yes/no)
    a. Active infection (acute or chronic)
    - Note past infections or no infections will be considered negative
    - Considers maternal placental blood only and includes active infections only
4. Antenatal peripheral malaria infection mother, (yes/no): malaria infection detected in the peripheral blood of the mother by either
    a. Standard microscopy at all antenatal visits
    b. PCR at all antenatal visits
    c. RDT at only unscheduled visits or at a scheduled visit with fever/ history of fever
    - Excludes RDTs at scheduled visits with no fever as this was a routine part of the ISTp intervention but not for the control arm

5. Antenatal 3rd trimester peripheral malaria infection mother at last scheduled visit in the 3rd trimester , (yes/no)
    a. Antenatal peripheral malaria infection mother detected during the last scheduled antenatal visit in the 3rd trimester, before delivery).
    b. Otherwise the same diagnostic criteria are used as for antenatal peripheral malaria infection mother, described in 4 above
6. Delivery peripheral malaria infection mother, (yes/no), detected by
    a. RDT (pLDH or HRP2 or both)
    b. Standard microscopy
    c. PCR
    - includes RDTs (as opposite to the definition of peripheral malaria used while she was still pregnant [antenatal])
7. Congenital malaria, (yes/no): malaria infection, detected in foetal cord blood or peripheral blood of the new born at birth or within 7 days (168 hours) after birth, by either
    a. RDT (pLDH only)
    b. Standard microscopy
    c. PCR
8. Infant malaria, (yes/no): malaria detected between days 8 and end of infant follow-up detected by either.
    a. RDT (pLDH or HRP2 or both)
    b. Standard microscopy
    c. PCR
9. Sub-patent peripheral malaria infection, (yes/no)
    a. PCR positive
    b. Microscopy and RDT negative (or one of the two if the other is missing)
10. Patent peripheral malaria infection, (yes/no)
    a. PCR positive or missing
    b. Microscopy and RDT positive (or one of the two if the other is missing). We will use RDT results first, and microscopy only if RDT results are missing because microscopy is less reliable.
11. Sub-patent placental malaria infection, (yes/no)
    a. PCR positive or Placental histology positive and
    b. Microscopy and RDT negative (or one of the two if the other is missing)
    - Note: Cannot be defined if both PCR and histology are missing
12. Patent placental malaria infection, (yes/no)
    a. PCR positive or Placental histology positive, or both missing and
    b. Microscopy and RDT positive (or one of the two if the other is missing)
    - Note: Can be defined if both PCR and histology are missing
    - The focus here is on ‘patent’; i.e. they must be RDT positive (or smear positive if RDT is missing). If both RDT and smear results are missing this variable cannot be defined.
13. Clinical malaria, (yes/no)
    a. Documented fever (>=37.5 oC), or recent history of fever in the last 24 or 48 hours, or other symptoms of acute illness that resulted in a women seeking care or alerting the study team to request a home visit

b. Maternal malaria patent infection detectable by Microscopy or PCR
- Excludes immediate follow-up visits related to the primary episode; if not defined, used 14 days exclusion period for that endpoint

14. Asexual parasite density by microscopy
    a. parasite density using natural log transformed densities, expressed per mm3, using either white cell count from complete blood counts, or assuming a white blood cell count of 8000/mm3.

Table 1: Definition malaria infection

		Placental or Peripheral Malaria infection (PPM)			Peripheral malaria infection (PRM)			Placental malaria (PLM)		
		Any	Patent	Sub-patent	Any	Patent	Sub-patent	Any	Patent	Sub-patent
HRP2-based RDT (HRP2)		Pos	Pos	Neg	Pos*	Pos*	Neg	Pos	Pos	Neg
pLDH-based RDT (pLDH)		Pos	Pos	Neg	Pos*	Pos*	Neg	Pos	Pos	Neg
Microscopy	Asexual (ASEX)	Pos	Pos	Neg	Pos	Pos	Neg	Pos	Pos	Neg
	Gametocytes (GAM)	Pos	Pos	Neg	Pos	Pos	Neg	NA	NA	NA
Placental histology	Active (PHA)	Pos	Pos	Pos	NA	NA	NA	Pos	Pos	Pos
	Past (PHP)	Pos	NA	NA	NA	NA	NA	Pos	Pos	NA
PCR peripheral blood (PCR_{MP})		Pos	Pos	Pos	Pos	Pos	Pos	NA	NA	NA
PCR placental blood (PCR_{PL})		Pos	Pos	Pos	NA	NA	NA	Pos	Pos	Pos

*for antenatal visits RDTs results are included for unscheduled visits only to avoid assessment bias because ISTP is an intervention based on RDT during scheduled visits; at delivery all RDTs results are included.

NA = Not applicable, Pos=Positive, Neg=Negative

At delivery, standard microscopy will only be considered if no other test results are available. RDT will be considered if PCR or histology are not available.

PCR and histology will be given equal weight; i.e. a placenta will be considered positive if sexual or asexual parasites or past infections are detected by histology or the PCR is positive.

5.2. Morbidity endpoint definitions

1. Birthweight (grams) (continuous); weight taken within 24 hours birth. Birthweights taken more than 24 hours after delivery will not be considered because of the physiological fall in birth weight in breastfed infants occurring in the first days following delivery.[1,2]
2. Corrected Birthweight (grams) (continuous); weight taken within 7 days (168 hours) after birth. Birthweights taken more than 24 hours after delivery will be corrected for the physiological fall in birth weight in breastfed infants occurring in the first days following delivery.[1,2] All primary analysis involving birth weights will refer to the corrected birth weights, but a sensitivity analysis will be conducted to explore the effect of the method used to correct birthweight as described in more detail in section 11.4.3.2, page 23

   a. Birth weights taken 24-48h hours, and 48-168 hours after delivery will be corrected by a factor +2% and +4%, respectively to obtain the estimated weight at birth.[3,4]
   b. Birth weights within 24 hours will not need to be corrected.
3. Gestational age (days) (continuous): derived gestational age at booking in days based on gestational age assessment methods at booking assessed in order of priority as follows:
   a. By ultrasound scan if booking was done at gestational age up to 168 days (24 weeks)
   b. By gestational age from Ballard score estimated at delivery if booking was done at gestational age greater than 168 days (24 weeks)
   c. By Last Menstrual Period if known and any of the preceding measures are not available
   d. By fundal height measurement if no other measure of gestational age is available.
4. Gestational trimester
   a. 2nd trimester: Gestational age from 98 – 195 days (14-27 weeks) inclusive
   b. 3rd trimester: Gestational age from 196 days (28 weeks) onwards
5. Birthweight (corrected) for gestational age percentile (WgAP) or Z-scores (WgAZ) (continuous variable)
   a. Gender specific reference developed by Schmiegelow will be used to calculate percentile or a Z score for Birth weight-for-gestational age.[5]
6. Corrected Low-birth-weight (LBW), (yes/no): corrected birth weight under 2,500 grams
7. Uncorrected Low-birth-weight (LBW), (yes/no): uncorrected birth weight under 2,500 grams measured within 24h of delivery.
8. Preterm birth (PTB), (yes/no): spontaneous birth before 259 days (37 weeks) gestation
9. Small-for-Gestational Age (SGA), (yes/no): fetal weight <10th percentile of gestation age (see birth weight for gestational age for reference population)
10. Miscarriage, (yes/no): Loss of foetus before 196 days (28 weeks) gestation.
11. Induced abortions, (yes/no): Intentional loss of a foetus before 196 days (28 weeks) gestation.
12. Still birth, (yes/no): Loss of foetus >= 196 days (28 weeks) gestation or later showing no signs of life.
13. Foetal loss, (yes/no): Stillbirth or miscarriage
14. Adverse live-birth outcome, (yes/no): composite endpoint defined as having a birth that fulfils the criteria for either:
   a. LBW or
   b. Preterm birth or
   c. SGA
15. Adverse any-birth outcome, (yes/no): composite endpoint defined as having a birth that fulfils the criteria for either:
   a. LBW
   b. Preterm birth
   c. SGA
   d. Still birth
   e. (Spontaneous) miscarriage
16. Perinatal death, (yes/no): still birth **or** death within 7 days of birth

17. Neonatal death, (yes/no): death within 28 days of birth (defined as '1' month or earlier for pragmatic reasons)
18. Maternal death, (yes/no): The death of a woman while pregnant or within 42 days of termination of pregnancy
19. Non-malaria sick-clinic visits, (yes/no)
    a. Documented fever (>=37.5 oC), or recent history of fever in the last 24 or 48 hours, or other symptoms of acute illness that resulted in a women or newborn seeking care or alerting the study team to request a home visit
    b. No evidence of peripheral malaria infection by RDT or microscopy
    - Excludes immediate follow-up visits related to the primary episode; if not defined, use 14 days exclusion period for that endpoint
    - Note: these events are mutually exclusive of clinical malaria (i.e. all-cause sick-clinic visits minus sick clinic visits due to clinical malaria = non-malaria sick-clinic visits)
    - Delivery visits will be ignored in the clinical visits analysis hence ignoring the placental information
15. All-cause sick-clinic visits, (yes/no)
    a. The sum of Clinical malaria and non-malaria sick-clinic visits
    - Excludes immediate follow-up visits related to the primary episode; if not defined, use 14 days exclusion period for that endpoint
16. All-cause non-sick clinic visits, (yes/no)
    a. Events, such as obstetric complications that resulted in a women or newborn seeking care or alerting the study team to request a home visit
    b. Excludes delivery visit and study specific visits related to scheduled follow-up or other study specific procedure that would other not have resulted in a clinic visit under routine conditions (e.g. to provide 2nd or 3rd day study drugs under DOT).
    - Excludes immediate follow-up visits related to the primary episode; if not defined, use 14 days exclusion period for that endpoint
    - Note: Events can overlap with clinical malaria or non-malaria sick visits
    - The term 'non-sick' implies the absence of an acute illness, such as those with fever.
17. All-cause clinic visits, (yes/no)
    a. The sum of all-cause sick and non-sick clinic visits (i.e. any unscheduled visit made for clinical reasons)
    - Excludes delivery visit and study specific visits related to scheduled follow-up or other study specific procedure that would other not have resulted in a clinic visit under routine conditions (e.g. to provide 2nd or 3rd day study drugs under DOT).
    b. Excludes immediate follow-up visits related to the primary episode; if not defined, use 14 days exclusion period for that endpoint
18. Maternal anaemia, (yes/no): Hb<11,0 g/dL
19. Maternal Moderate to severe anaemia, (yes/no): Hb<9,0 g/dL
20. Maternal severe anaemia, (yes/no): Hb<7,0 g/dL
21. Foetal anaemia, (yes/no): Hb<12,5 g/dL in umbilical cord blood at birth, which is 2 standard deviations below the mean cord Hb in developed countries[6]

22. Congenital malformations, (yes/no): Physical abnormality of baby detected at delivery or newly noted abnormality during the infant visits.
23. Neonatal jaundice, (yes/no): Reported presence of jaundice in neonate within 24 hours and at day 7 follow-up visit.
24. Laboratory test results outside of normal range, (yes/no): As defined in the study SOP for biochemistry and haematological parameters

5.3. Definitions for other Endpoints

1. Treatment Compliance with IST-DP (yes/no):
    a. With each course: 3 out 3 daily doses of DHP
    b. With the overall regimen: >2/3 or 3/4 of scheduled visits (subject to gestational age at enrolment, and gestational age at delivery [exclude visits that could not have occurred because the woman delivered before that scheduled visit date)
2. Treatment compliance with IPTp-SP (yes/no):
    a. With each dose: took required dose as DOT at visit
    b. With the overall regimen: >2/3 or 3/4 of scheduled visits (subject to gestational age at enrolment, and gestational age at delivery [exclude visits that could not have occurred because the woman delivered before that scheduled visit date)
3. Treatment compliance (quintiles): Treatment compliance will be defined as a percentage (total number of tablets taken/total number of tablets expected)*100, and divided into 3 equal groups (terciless).
4. Treatment compliance (continuous): Treatment compliance will be defined as a percentage (total number of tablets taken/total number of tablets expected)*100, and treated as a continuous variable.
5. Regimen compliance will be defined as a percentage of the number of scheduled visits attended (total number of scheduled visits attended/total number of scheduled visits expected by gestational age at enrolment and delivery)*100, and then ranked into 5 equal groups (quintiles).
    a. Exclude visits that could not have occurred because the woman delivered before that scheduled visit date.
6. SP Day-1 Dose intolerance (%), IPTp-SP (yes/no): Vomited SP and did not tolerate a repeat dose (vomited again) or was not given a repeat dose.
7. DP Day-1 dose intolerance (%): Vomited DP on day-1 and did not tolerate a repeat dose (vomited again) or was not given a repeat dose (where day-1 is the first dose day).
8. DP Day-2 dose intolerance (%): Vomited DP on day-2 and did not tolerate a repeat dose (vomited again) or was not given a repeat dose.
9. DP Day-3 dose intolerance (%): Vomited DP on day-3 and did not tolerate a repeat dose (vomited again) or was not given a repeat dose.
10. DP course intolerance (%): DP Day-1, or Day-2 or Day-3 dose intolerance.
11. Day-1 regimen intolerance risk (%): SP Day-1 or DP Day-1 dose intolerance at least once.
12. DP regimen intolerance (%): DP course intolerance at least once

5.4. Definitions for other variables

1. Season (terciles): Each pregnancy will be defined to have occurred in the predominantly rainy vs dry season using rainfall data collected in the study area. This will be done by categorising the women into three equal groups based on the mean daily, weekly or monthly rainfall during the 6 months period prior to the date of delivery (i.e. during the 2nd and 3rd trimester of pregnancy). This can include rainfall data prior to her enrolment in the study.
2. Gravidity will be computed and triangulated from the various variables in the enrolment questionnaire and categorised into nominal (not ordinal) categorical variables. The nominal variable will be used because the relationship between gravidity and the primary outcomes is not linear. The following categories will be used:
    a. Gravidity-2 (G1-G2, G3+)
        i. Pauci-gravidae: first and second pregnancies (G1-G2)
        ii. Third or more pregnancy G3+
        - This will not be computed but will be based on the strata at enrolment (study codes following the first digit that were <600 are G1-G2, code > 600 G3+).
    b. Gravidity-4, (G1, G2, G3, G4+):
        i. First pregnancy (G1)
        ii. Second pregnancy (G2)
        iii. Third pregnancy (G3)
        iv. Fourth or more pregnancy G4+
        - Computed based on the combination of variables in the booking form (‘Primi yes/no, parity, previous livebirths, stillbirths and miscarriages).
        - The gravidity strata used at randomisation (G1-G2 vs G3+) supersedes any discrepant computed values. i.e. women who were enrolled in the G1-G2 strata but were later computed to be more likely to be gravidae 3 or greater based on their detailed obstetric history will be considered as gravidae 2 in the variable gravidity-4. Similarly women enrolled as G3+, but computed as G2 or G1, will be considered as G3 in the variable gravidity-4.
3. Educational status, (low, medium, high): In accordance with the completed years of schooling the pregnant women will be ranked and categorized into 3 equal groups as low, medium, or high education; if data is incomplete, this will be changed into primary school not completed, completed, secondary school attended/completed.
4. Socio-Economic Status (SES), (low, medium, high): Categories will be based on the rank position of the computed wealth index based on the computed index score for SES which takes type of house, ownership variables etc into account.
5. Study site, (Mpemba, Madziabango and Chikhwawa); will be based on site of enrolment, not site of delivery.
6. Place of residence (Urban, peri-urban, rural, not-known): will be based on information provided in the enrolment forms.

6. Study Endpoints

6.1. Primary outcome

6.1.1. *First and second pregnancies*

For women in their first or second pregnancy, the primary outcome is a composite of adverse live-birth outcomes and will be defined as

1. SGA, **or**
2. Preterm, **or**
3. LBW

Birthweight, corrected for time of assessment will be used.

6.1.2. *Multigravidae*

In multigravidae, the primary endpoint will be any active or recent malarial infection at delivery, any species, detected at the time of delivery in either

1. placental blood (placental malaria), **or**
2. placental tissue detected (placental malaria by placental histology), **or**
3. peripheral maternal blood (maternal malaria)

6.2. Secondary efficacy outcomes

6.2.1. *Antenatal*

1. Occurrence of maternal malaria infection during pregnancy starting 1 day after the first intervention event (SP or first screening event) (yes/no) by
   i. PCR alone,
   ii. RDT alone,
   iii. microscopy alone,
   iv. composite infection endpoint (i.e PCR or RDT or microscopy)
2. Occurrence of clinical malaria episodes starting 1 day after the first intervention event (SP or first screening event) (yes/no)
4. Occurrence of Non-malaria sick-clinic visits; All-cause sick-clinic visits, All-cause non-sick clinic visits and All-cause clinic visits (yes/no)
3. Maternal haemoglobin (g/L)and prevalence anaemia at term (yes/no)
   i. Maternal haemoglobin (g/L)
   ii. Any anaemia
   iii. Moderate anaemia (yes/no)
   iv. Severe anaemia (yes/no)
4. Prevalence of malarial infection at last scheduled antenatal visit in the 3rd trimester prior to delivery).

6.2.2. *At delivery*

1. Prevalence of placental malaria (any species), (yes/no).
2. Prevalence of placental malaria infection by placental histology, (yes/no)
   a. Past infection

   b. Active infection
      i. Overall
         1. Acute
         2. Chronic
3. Prevalence of maternal malarial infection by:
   i. PCR alone,
   ii. RDT alone,
   iii. microscopy alone,
   iv. composite infection endpoint (i.e PCR or RDT or microscopy)
4. Asexual and sexual stage parasite density. This will be peripheral blood for peripheral smears and placental impression smear for placental smears.
5. Birthweight (corrected)
   a. LBW (yes/no)
   b. Birthweight (gram)
6. Birthweight (uncorrected)
   a. LBW (yes/no)
   b. Birthweight (gram)
7. Gestational age
   a. Preterm birth (yes/no)
   b. Gestational age (week)
8. BirthWeight for gestational age z-score or percentile
   a. SGA
   b. WfAP or WfAZ
9. Maternal haemoglobin (g/L) and anaemia at delivery (yes/no)
   a. At delivery
      i. Maternal haemoglobin (g/L)
      ii. Any anaemia (yes/no)
      iii. Moderate-severe anaemia (yes/no)
      iv. Severe anaemia (yes/no))
10. Still birth (yes/no)
11. Spontaneous abortion (yes/no)
12. Induced abortion (yes/no)
13. Spontaneous Foetal loss (spontaneous abortions or still birth) (yes/no)
14. Adverse any-birth outcome (yes/no)

6.2.3. *After delivery*

1. Prevalence of congenital malaria (yes/no)
2. Incidence of Infant malaria (yes/no)
3. Incidence of infant non-malaria sick-clinic visits; All-cause sick-clinic visits, All-cause non-sick clinic visits and All-cause clinic visits
4. Foetal haemoglobin (g/L) and anaemia (yes/no)
5. Perinatal mortality (yes/no)
6. Neonatal mortality (yes/no)
7. Cumulative infant mortality by end of follow-up (about 6 to 8 weeks after birth)

6.3. Secondary safety outcomes

1. Incidence of any maternal SAEs reported during pregnancy,
    a. Overall
    b. by system organ class and preferred term
2. Incidence of any infant SAEs reported in the infant by end of follow-up period.
    a. Overall
    b. by organ class and preferred term
3. Severe cutaneous skin reaction in the mothers within 30 days of drug intake as reported as SAE during the trial.
4. Maternal deaths (mortality rate)
5. Congenital malformations.
6. Neonatal jaundice by day 7
7. Laboratory test results outside of normal range.

6.4. Tolerability outcomes

1. Incidence of non-serious adverse events
    a. in the mothers
    b. infant
2. Compliance with study each study course (see definitions)
3. Compliance with overall intervention regimen (see definitions)
4. Treatment tolerance
5. SP and DP Day-1 dose tolerance (for comparison between treatment arms)
6. Day-1 regimen intolerance risk (for comparison between treatment arms)
7. DP-course tolerance (IST arm only)
8. DP-regimen tolerance (IST arm only)

7. Analytical population

7.1. Full analysis population for modified Intention to treat analysis (ITT)

The primary analyses will be a modified ITT, including all randomised women for whom there is an outcome.

7.2. Per protocol Population (PP);

Per protocol population will be defined as:

1. All randomised women that either
    a. Received the study intervention (IPTp-SP or ISTp-DP) on every scheduled visit at least four weeks apart and took all the study doses on each occasion when measured; or
    b. Reached a study end-point before completion of the three-visit schedule but received the intervention at least once; or

   c. Received an approved alternative treatment for symptomatic malaria according to protocol that replaced the need for the scheduled intervention.
2. Women who contributed information to the specific endpoint investigated

Women will be excluded from the per protocol population if they
1. Did not adhere to study treatment (took less thanthe recommended dose over recommended time period on each occasion treatment is required' or
2. 'Used prohibited medication'
3. Were screening failures

7.3. Safety Population

All women who received the study intervention; i.e IPTp-Sp or ISTp (with DHP if malaria-positive), and have complete sufficient follow-up to provide information on potential adverse events, defined as attendance of the next scheduled study visit from the last dose of investigational product received.

8. General analytical approach

8.1. Reporting guidelines

We will follow the Consolidated Standards of Reporting Trials (CONSORT) guidelines for reporting of clinical trials (http://www.consort-statement.org/).

8.2. Pooling of data from the 3 sites

The data from all 3 investigational sites (Mpemba, Madziabango and Chikhwawa) will be pooled because the study is conducted under a common protocol with the intention of pooling the data for analysis.

8.3. Pre-scheduled stopping of study participants and use of data

In case the intervention was stopped before the pre-scheduled end, either by a decision of the study woman herself, or by the study team, and data was collected after stopping the intervention, the information will be included in the full analyses set. Missing data handling

8.4. Missing data

Missing data will be dealt with differently for the primary endpoint and the independent variables as follows.

8.4.1. *Endpoints*

Missing data on the primary and secondary endpoints will not be imputed.

8.4.2. *Covariates*

Missing values for 9 pre-selected covariates in Section 11.3.3, page 22 will be imputed for the covariate adjusted analysis of primary endpoint.

If the missing data for all 9 pre-selected covariates is less than 1% of observations, simple imputation will be employed: for a continuous variable, normal distribution from sample mean and standard deviation will be used to replace the missing value; for a binary variable, binomial distribution from sample distribution will be used to impute the missing value. Seed for the imputation will be set as 2802.

If the missing data for all 9 pre-selected covariates is large than 1% and less than 20% of observations, missing values for these covariates will be imputed by means of multiple imputations (10 multiple datasets will be created) using the SAS procedure MI. Seed for the imputation will be set as 2902. Missing data will be assumed missing at random (MAR), (probability that an observation is missing can depend on the observed values of the individual, but not on the missing variable values of the individual). Imputations will be done on continuous as well as categorical variables. If categorical variables are created from continuous variables the imputations will be conducted on the continuous variable. We will first investigate the MCAR assumption by modelling the probability of missing data on treatment assignment and other independent variables. If any of the independent variables are significant then missing data depends on covariates, a violation of MCAR. Then missing data will be assumed MAR. Results under MAR imputation and without multiple imputation will be compared in a sensitivity analyses. Models under Missing Not At Random assumption (selection and pattern mixture) will not be done. Focus will be on MAR assumption and how its violation can be investigated in a sensitivity analysis.

8.5. Stratification by gravidity

All analysis will present results stratified by gravidity group and overall (summary estimate for all gravidae obtained by stratified analysis). Women will be included in the gravidity strata that they were allocated to at enrolment ('Gravidity-2') and no switching will occur to other strata.

8.6. Linear regression analysis for adjusted analysis of dichotomous outcomes

8.6.1. *Risk ratios or odds ratios?*

Because the study outcome will be common in some strata the odds ratio is not likely to approximate the risk ratio and be further from 1 than the risk ratio (i.e. more extreme). Because risk ratios are easy to interpret and because odds ratios are sometimes misinterpreted as risk ratios, the study will use risk ratios as the measure of relative association for dichotomous outcomes to assist the public health interpretation of the findings. Furthermore risk ratios, but not odds ratios, have a mathematical property called collapsibility; which is an advantage in the interpretation of analyses that use pooled estimates obtained from stratified analysis[7] as proposed in this study.

8.6.2. *Log binomial regression and alternative strategies in case of non-convergence*

The primary linear regression analysis method to obtain risk ratios and corresponding 95% Confidence Intervals (CI) will be log binomial regression. A well know limitation of log-binomial

regression is problems with convergence. If a model does not converge with the default syntax for log-binomial regression, one of the options as proposed by Cummings, The Stata Journal (2009) **9**, Number 2, pp. 180-181 will be used,[8] which include using the 'difficult' option in Stata, or the binreg option (which uses iterated, reweighted least squares instead of maximum likelihood); and the weighted copy number method,[9].without or with the 'difficult' option.

If the log binomial methods described above still does not result in convergence, we will use generalized linear regression with a log link, but a normal distribution (instead of binomial distribution), and robust variance estimator (without or with the difficult option), and lastly use of generalized linear models with a log link and a Poisson distribution (Poisson regression), again with the robust variance estimator and without or with the difficult option. If the normal distribution or Poisson distribution needs to be used for some effect measures because of non-convergence of the log binomial model, this will be indicated by an asterisks or equivalent in the Tables and Figures. Because there is a debate regarding the merits of using Gaussian or Poisson regression to estimate prevalence risk ratios, we will also report the Odds ratio obtained from logistic regression models in the table or figure notes for these effect estimates.

8.7. Reporting conventions

8.7.1. *Descriptive statistics*

Variables will be checked for the presence of outliers, using tabulation and box plots. Continuous variables with an approximately normal distribution will be summarised by their mean, standard deviation and skewed continuous variables by their median and the interquartile range (25th percentile to 75th percentile). Parasite densities will be log-transformed and expressed as the geometric mean. Categorical variables will be summarised by their frequency and percentage.

Means, standard deviations and any other statistics other than quartiles will be reported to one decimal place greater than the original unit of measure. Quartiles, such as median, or minimum and maximum will use the same number of decimal places as the original data. Estimated parameters, not on the same scale as raw observations (e.g. regression coefficients) will be reported to three significant figures.

8.7.2. *Measures of associations and P-value reporting*

All analyses will be conducted at the 5% significance level. Estimates and their 95% confidence intervals (CI) will be produced using SAS 9.3 and STATA v 12 (versions may change). We will also report p-values. P-values ≥0.01 will be reported to three decimal places in the analysis and two decimals places in the manuscript; p-values less than 0.001 will be reported as '<0.001'.

9. Participant disposition and Flow chart

A flow chart (Figure 1: Trial Flowchart, page 28) will be drawn up showing the number of women screened, enrolled, and followed-up, and the number contributing to the full analysis and per-protocol. The number screened and not enrolled and the reasons for non-enrolment will be reported, and well as the number and reasons of women who were lost after randomisation, or for whom the intervention was stopped before its pre-scheduled end.

10. Baseline data summaries

10.1. Demographic, clinical and laboratory measures

All baseline characteristics will be summarised by intervention group and overall. No inference testing will be conducted on the baseline variables, but marked differences (e.g. >10% relative difference) will be noted and taken into account in the post-hoc multiple regression analyses.

10.2. Measures of Social Economic Status (SES) Asset index.

The educational, income and socio-economic status parameters will be summarised in table form. and consists of 23 variables. To develop a single measure of SES index Principal Component Analysis (PCA) will be used to generate scores for ranking. PCA is a multivariate data analysis technique and it will reduce the dimension of this pool of variables to a smaller set of principal components capturing as much information (variability) from the data as possible. The summary SES index will be added to the baseline table.

11. Efficacy analyses

11.1. Measure of associations

11.1.1. *Binary outcomes*

For binary endpoints, the following will be calculated:

11.1.1.1. **Unadjusted**

1. Crude (unadjusted) prevalence data (numerator, denominator, % per arm)
2. Crude risk ratio (RR) (95% CI)
3. P-value for crude difference in prevalence
4. Crude risk difference (RD) (95% CI)
5. Numbers Needed to Treat (for primary endpoints only, unadjusted data)

11.1.1.2. **Adjusted**

6. Adjusted risk ratio
7. Adjusted risk difference (95% CI)
8. P-value for adjusted analysis

We will report the RR values as relative risk reduction (RRR), which will be calculated as (RRR=100%x[1-RR]) and can be expressed as a percentage.

11.1.2. *Continuous outcomes*

Differences in continuous endpoints will be assessed by linear regression analysis. For continuous endpoints, the following will be calculated:

11.1.2.1. **Unadjusted**

1. Crude mean and SD per arm

2. Crude mean difference (95% CI)
3. P-value for crude mean difference

11.1.2.2. **Adjusted**

4. Adjusted mean difference (95% CI)
5. P-value for adjusted mean difference

11.2. Forest plots of efficacy parameters

Results will be presented using forest plots for dichotomous and continuous variables. The graphics component will represent primary measure of association, i.e. the crude and adjusted Relative Risk Reduction and the adjusted and crude mean difference. In addition columns with number of events and women per group, and Risk Difference (RD) (dichotomous variables) and the number of women, mean (SD) per group, and crude mean difference (95%) (Continuous variables) will be added.

11.3. Primary efficacy endpoint analysis

11.3.1. *Crude and adjusted effect estimates*

The primary analyses will be a modified ITT using the full analytical population (7.1, page 16). The primary measures of association are the

1. Absolute difference in proportions (95% CI) between the two treatment groups using the using the generalized linear regression model with binomial distribution and identity link function.
2. A risk ratio (RR) (95% CI) between the two groups obtained using the generalized linear regression models with binomial distribution and log link function. (see 8.6, page 18).

In women in their first and second pregnancies, the primary efficacy endpoint will be the composite adverse birth outcomes and for multigravidae (G3+) this will be any active or recent malarial infection at delivery (see 6.1.1, page 14).

Both the crude (unadjusted) risk ratio (RR) and the adjusted RR will be computed using the generalised linear regression model (see 8.6, page 18). In the first model the response variable is the primary endpoint variable (yes/no) and the independent variable is treatment group. No adjustment will be made for site.[10] In the second model, additional independent variables will be included to adjust for potential confounding, stratified by gravidity and site. The independent variables for adjustment are given in Section 11.3.3, page 22.

Similar considerations will be used for the assessment of differences in continuous endpoints by multiple linear regression models with independent variables as treatment group. .

11.3.2. *Adjustment for baseline independent variables in the multiple regression models*

The aim of the modelling is to obtain a valid estimate of an exposure-disease relationship i.e. a valid measure of the treatment effect of IST-DP relative to the control arm, adjusted for confounding. Because the design used stratification by site (Mpemba, Chikwawa and

Madziabango) and gravidity we will use stratified covariate adjusted analysis with site (3 strata) and 'gravidity-2' (2 strata: G1-2 and G3+) as variables for stratification. We will use the same independent co-variates in each multivariate model to allow for consistency across the models. The variables will be categorised into two groups (dichotomous) or into 3 equally sizes sub-groups (terciles) as indicated below (Section 11.3.3), such that the same variables will also be used for sub-group analysis (Section 11.4.2), except for gravidity that will be used as 'gravidity-4' (G1, G2, G3, G4+) when treated as co-variate (also in the gravidity-2 stratified co-variate models) and as 'gravidity-2' (G1-G2, G3+) in the sub-group analysis (See Section 5.4, Definitions for other variables, page 13).

11.3.3. *Variable specification*

Variables that will be included will include variables that are likely to be prognostic for the primary outcome but are not in the causal pathway, as predefined on the basis of the literature, and variables that are possibly prognostic for the primary outcome.

The following variables will be considered a priori:

Likely to be prognostic

1. gravidity (also within gravidity strata) (variable 'gravidity-4' (G1, G2 for G1-G2 strata, and G3, G4+ for multigravidae strata)
2. Participant enrolment site (Madziabango, Mpemba, and Chikwawa)
3. Malaria status at enrolment (pos, neg)
4. Season during pregnancy (rainy, post-rainy, dry)
5. Maternal height (terciles)

Possibly prognostic

1. Haemoglobin status at enrolment (terciles)
2. Social Economic Status (terciles)
3. Corrected gestational age at booking (<=median,>median)
4. Education status (terciles)

11.4. Secondary efficacy analysis of primary outcomes

11.4.1. *Overall pooled effect (all gravidae)*

Two secondary analyses emanating from the primary analyses will be conducted as follows;

1. The first analysis will repeat the analyses for the composite primary morbidity outcome (LBW, preterm birth, or SGA) on all women pooled, by stratified analysis.
2. The second analysis will repeat the analyses on the composite outcome of 'malaria infection at delivery' on all women pooled

11.4.2. *Subgroup analysis*

We will include an interaction between treatment group and the following factors below in separate models to assess to what extent the effect of the intervention on the primary endpoints is influenced by the variables defined in Section 11.3.3, page 22. Because the study was not designed to have sufficient power for tests for interaction terms in these subgroup analysis, we will interpret the results cautiously. Results will be presented as forest plots.

For the variable study site, women enrolled in one site, but followed in another site, will be analysed using the site of enrolment.

Subgroup analysis will be based on the completed cases without imputing missing values for covariate variables.

The subgroup analysis of primary endpoint will be summarised in the forest plot.

11.4.3. *Sensitivity analysis*

11.4.3.1. Imputations

The results of the statistical models after multiple imputation for missing values for the independent covariates will be compared with the initial results before imputation in a sensitivity analyses. The final results are those without multiple imputation, so in an event that there are differences between these results and those with multiple imputations those without multiple imputations will still be taken as the final results. Nonetheless the differences will be explicitly explained.

11.4.3.2. Corrected birth weights

The results of the statistical models using uncorrected birthweight will be compared with the initial results using corrected birthweights in a sensitivity analyses. Three different sensitivity analysis will be conducted: 1 using birthweight collected with 24 hours of birth (these are all uncorrected by definition), and one using all birthweight collected within 1 week, but without correction, and one using uncorrected birthweights collected within 1 week, but using timing of measurement as co-variate. In an event that there are differences between these results (e.g. >10% relative difference in effect estimate [e.g. RR 1.4 vs RR 1.6]) the results without correction will be taken as the final results. If the difference is <10% the corrected birthweight will be used (as this results in a bigger sample and minimizes the potential for overestimation of the frequency of small for gestational age). Any differences will be explicitly explained.

11.5. Secondary efficacy outcomes

11.5.1. *Outcomes at delivery*

The secondary efficacy outcomes outlined in Section 6.2.2, page 14 will be analysed using similar crude and adjusted analysis. For the modelling approaches, the same independent variables as identified in the models for the two primary endpoints will be used for adjustment. Results will be expressed identical to the methods described above for the primary outcomes.

11.5.2. *Count data outcomes*

For the secondary efficacy outcomes that are count of episodes during follow up, these outcomes will be analysed using Poisson regression with the time of follow up as an offset. The incidence rate ratio for the treatment group effect will be estimated and its 95% CI presented.

In an event of over dispersion, then the Negative binomial regression model will be fitted to the data instead of the Poisson regression model. In an event where the number of episodes is very small and there are lots of zero episodes then a log binomial model will be fitted to the data where

the dependent variable will be defined as (0=no episodes, 1=one or more episodes). A zero-inflated Poisson regression model will also be fitted to the data in an event of a lot of zero adverse events. All these models will be compared using the Akaike Information Criteria (AIC). A model with a smaller AIC will be considered as the final model under these conditions.

Both crude and adjusted analyses will be conducted similar to the primary endpoints. Variables considered for the full models will be the same as those for the primary endpoints.

12. Safety analysis

For each safety outcome, the number of events and incidence of SAEs will be tabulated by system organ class, preferred term and by severity and causal relationship with the study drugs. Further Adverse Events analysis.

All adverse events will be categorised as serious or non-serious. The total number of adverse events will be a count outcome and will be analysed using similar methods for count data as above.

13. Other analysis

13.1. Number of intervention visits

Because the study was designed to allow variation between the number of scheduled visits as a function of the gestational age at enrolled (4+ scheduled follow-up visits for women who were enrolled early in pregnancy and 3 for women enrolled later in pregnancy), we will explore the difference in treatment effect on the primary endpoint between 3 and 4+ scheduled visits among the per protocol population. This will be done by including interactions between the number of scheduled visits (3 vs 4+) and treatment group so that an estimate of the comparison between the two treatment groups is estimated for each of these two strata.

13.2. Compliance with SP and DHA-PQ.

For definitions of treatment and regimen compliance measures see Section 5.3, Definitions for other variables, page 13. Further exploratory analysis will be conducted of the distribution and impact of regimen compliance on the primary endpoints. This will be done by including interactions between regimen compliance and treatment group so that an estimate of the comparison between the two treatment groups is estimated at each level of compliance. This will be done using quintiles of the regimen compliance variable to explore the shape of the relationship, as well as a continuous variables (0 to 100%). This analysis will exclude women who delivered prior to their last scheduled pre-natal visit. We will look at determinants of compliance, including whether dose intolerance is a predictor of subsequent compliance.

13.3. Treatment response

The % of women who were parasitaemic at each visit and are still parasitaemic at the next visits (defined as within 63 days inclusive [i.e. 9 weeks, the time typically used in extended in-vivo tests] will be compared between the women who received SP and DSHA-PQ using survival analysis and

the hazard ratio (95% CI CI) reported for 28 (+/- 3 days) (about 1 visit later), 42 days (+/- 3 days) and 63 days (=/- 3 days; i.e. about 2 months). Only fully treatment adherent women will be included in this analysis (for definition, see Section 5.3, Definitions for other variables, page 13).

14. References

1. Noel-Weiss J, Courant G, Woodend AK, 2008. Physiological weight loss in the breastfed neonate: a systematic review. Open Med 2: e99-e110.
2. Flaherman VJ, Kuzniewicz MW, Li S, Walsh E, McCulloch CE, Newman TB, 2013. First-day weight loss predicts eventual weight nadir for breastfeeding newborns. Arch Dis Child Fetal Neonatal Ed 98: F488-92.
3. Greenwood BM, Greenwood AM, Snow RW, Byass P, Bennett S, Hatib-N'Jie AB, 1989. The effects of malaria chemoprophylaxis given by traditional birth attendants on the course and outcome of pregnancy. Trans R Soc Trop Med Hyg 83: 589-94.
4. D'Alessandro U, Langerock P, Bennett S, Francis N, Cham K, Greenwood BM, 1996. The impact of a national impregnated bed net programme on the outcome of pregnancy in primigravidae in The Gambia. Trans R Soc Trop Med Hyg 90: 487-92.
5. Schmiegelow C, Scheike T, Oesterholt M, Minja D, Pehrson C, Magistrado P, Lemnge M, Rasch V, Lusingu J, Theander TG, Nielsen BB, 2012. Development of a fetal weight chart using serial trans-abdominal ultrasound in an East African population: a longitudinal observational study. PLoS One 7: e44773.
6. Brabin B, 1992. Fetal anaemia in malarious areas: its causes and significance. Ann Trop Paediatr 12: 303-10.
7. Cummings P, 2009. The relative merits of risk ratios and odds ratios. Arch Pediatr Adolesc Med 163: 438-45.
8. Cummings P, 2009. Methods for estimating adjusted risk ratios. The Stata Journal 9: 175-196.
9. Lumley T, R. Kronmal R, Ma S, 2006. Relative risk regression in medical research: Models, contrasts, estimators, and algorithms. Working Paper 293, . UW Biostatistics Working Paper Series. http://www.bepress.com/uwbiostat/paper293.
10. Kahan BC, Morris TP, 2013. Adjusting for multiple prognostic factors in the analysis of randomised trials. BMC Med Res Methodol 13: 99.

15. Tables and Figures

15.1. List of tables

**Section 1**	**Patient disposition**
Table 1.1	Patient disposition by treatment
Table 1.2	Patient disposition by gravidity group and treatment
**Section 2**	**Baseline information**
Table 2.1	Demographic characteristics of patients
Table 2.2	Bednet use and IRS
Table 2.3	Obstetric History
Table 2.4	Information copied from ANC card
Table 2.5	Ultrasound
Table 2.6	Medical history/ Prior and concomitant medication
Table 2.7	Physical examination
Table 2.8	Laboratory results
Table 2.9	Social economic status
**Section 3**	**Medication**
Table 3.1	Prior medication
Table 3.2	Concomitant medication
**Section 4**	**Follow-up information**
Table 4.1	Antenatal visit
Table 4.2	Maternal postnatal visit
Table 4.3	Infant visit
Table 4.4	Histopathology evaluation results
Table 4.5	Infant Health Facility Use
Table 4.6	Maternal malaria smear results
Table 4.7	Placental impression smear results
**Section 5**	**Delivery information**
Table 5.1	Delivery visit
Table 5.2	Delivery ballard score
Table 5.3	Delivery malaria smear test
Table 5.4	Delivery surface examination
**Section 6**	**Adverse events**
Table 6.1.1	Summary of adverse event: Mothers
Table 6.1.2	Summary of adverse event by system organ class and preferred term: Mothers
Table 6.1.3	Summary of serious adverse event by system organ class and preferred term: Mothers
Table 6.1.4	Summary of adverse event by severity, system organ class and preferred term: Mothers
Table 6.1.5	Summary of adverse event by causality, system organ class and preferred term: Mothers
Table 6.2.1	Summary of adverse event: Children
Table 6.2.2	Summary of adverse event by system organ class and preferred term: Children
Table 6.2.3	Summary of serious adverse event by system organ class and preferred term: Children

Table 6.2.4	Summary of adverse event by severity, system organ class and preferred term: Children
Table 6.2.5	Summary of adverse event by causality, system organ class and preferred term: Children
**Section 7**	**Efficacy**
Table 7.1	Primary outcome analysis
Table 7.2	Secondary efficacy outcomes
Table 7.3	Secondary safety outcomes
Table 7.4	Malaria infection endpoint definitions
Table 7.5	Morbidity endpoint definitions
**Section 8**	**Covariate adjusted analysis**
Table 8.1	Primary outcome analysis
**Section 9**	**Subgroup analysis**
Table 9.1	Subgroup analysis of primary outcome

Patient disposition (Flow Chart)

Figure 1: Trial Flowchart (Patient disposition)

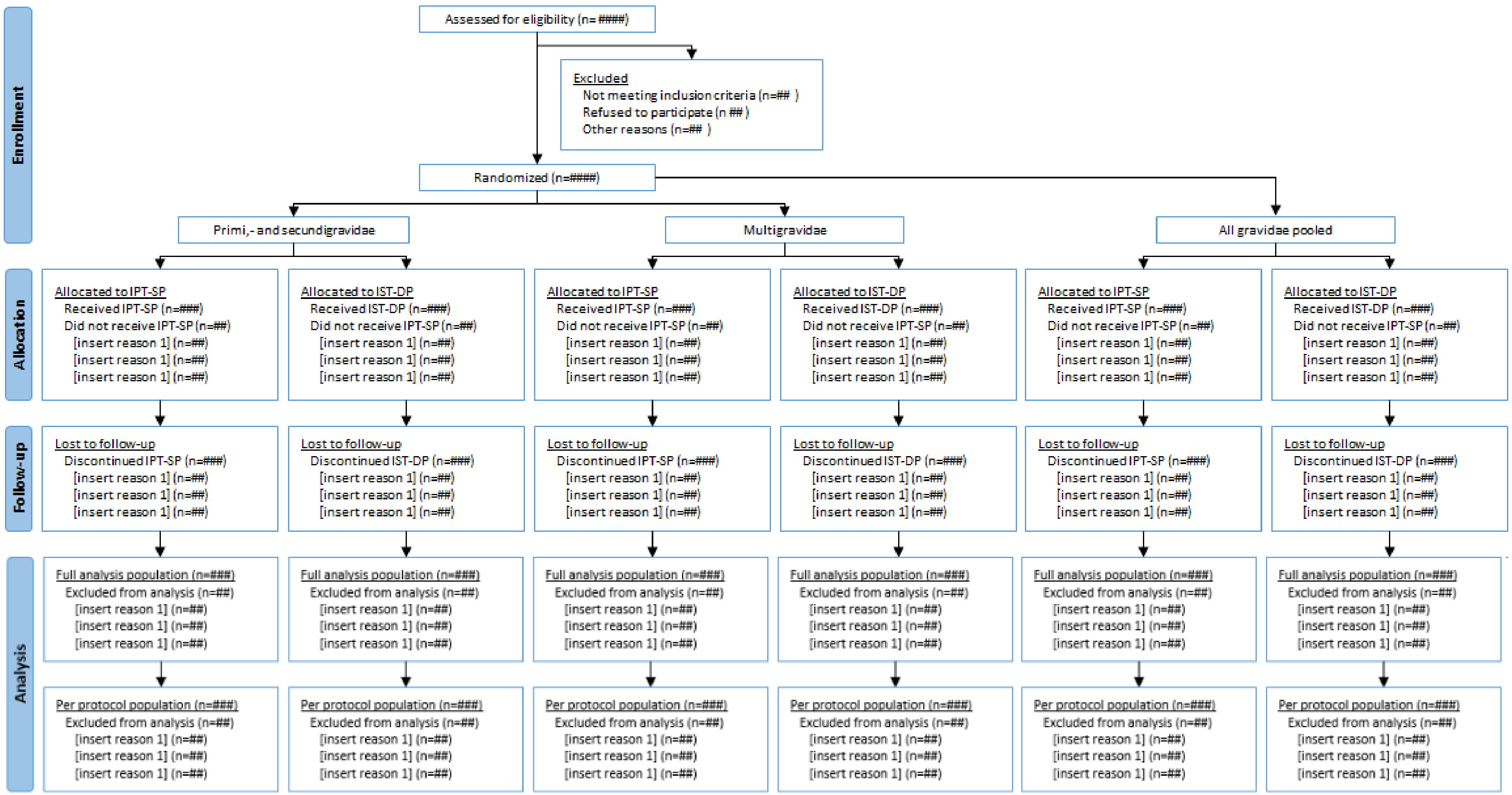

[insert flowchart notes here] (another 2 columns will be added for 'all gravidae')

15.2. Versioning manuscript

15.2.1. Word document

The manuscript drafts will be called ISTp-Malawi manuscript [ddmmmyy].Docx. Versioning is done by use of date in the file name (e.g. 'ISTp-Malawi-03Aug14.docx'). If colleagues comment on a draft, they add their initials at the end of the version they comment on; e.g. original is created by Mwayi called 'ISTp-Malawi-03Aug14.docx'. Feiko ter Kuile comments on this version and sends the comments in a file named 'ISTp-Malawi-03Aug14-FtK.docx' using tracked changes, where possible.

15.2.2. Endnote

The endnote file will be called 'ISTp-Manuscript.enl', and will have no versioning because endnote embeds the library in the word file.

16. Appendix 1: CONSORT checklist of information to include when reporting a trial*

Section/Topic	Item No	Checklist item	Reported on page No
**Title and abstract**			
	1a	Identification as a randomised trial in the title	
	1b	Structured summary of trial design, methods, results, and conclusions (for specific guidance see CONSORT for abstracts)	
**Introduction**			
Background and objectives	2a	Scientific background and explanation of rationale	
	2b	Specific objectives or hypotheses	
**Methods**			
Trial design	3a	Description of trial design (such as parallel, factorial) including allocation ratio	
	3b	Important changes to methods after trial commencement (such as eligibility criteria), with reasons	
Participants	4a	Eligibility criteria for participants	
	4b	Settings and locations where the data were collected	
Interventions	5	The interventions for each group with sufficient details to allow replication, including how and when they were actually administered	
Outcomes	6a	Completely defined pre-specified primary and secondary outcome measures, including how and when they were assessed	
	6b	Any changes to trial outcomes after the trial commenced, with reasons	

Sample size	7a	How sample size was determined	
	7b	When applicable, explanation of any interim analyses and stopping guidelines	
Randomisation:			
Sequence generation	8a	Method used to generate the random allocation sequence	
	8b	Type of randomisation; details of any restriction (such as blocking and block size)	
Allocation concealment mechanism	9	Mechanism used to implement the random allocation sequence (such as sequentially numbered containers), describing any steps taken to conceal the sequence until interventions were assigned	
Implementation	10	Who generated the random allocation sequence, who enrolled participants, and who assigned participants to interventions	
Blinding	11a	If done, who was blinded after assignment to interventions (for example, participants, care providers, those assessing outcomes) and how	
	11b	If relevant, description of the similarity of interventions	
Statistical methods	12a	Statistical methods used to compare groups for primary and secondary outcomes	
	12b	Methods for additional analyses, such as subgroup analyses and adjusted analyses	
**Results**			
Participant flow (a diagram is strongly recommended)	13a	For each group, the numbers of participants who were randomly assigned, received intended treatment, and were analysed for the primary outcome	
	13b	For each group, losses and exclusions after randomisation, together with reasons	
Recruitment	14a	Dates defining the periods of recruitment and follow-up	

	14b	Why the trial ended or was stopped	
Baseline data	15	A table showing baseline demographic and clinical characteristics for each group	
Numbers analysed	16	For each group, number of participants (denominator) included in each analysis and whether the analysis was by original assigned groups	
Outcomes and estimation	17a	For each primary and secondary outcome, results for each group, and the estimated effect size and its precision (such as 95% confidence interval)	
	17b	For binary outcomes, presentation of both absolute and relative effect sizes is recommended	
Ancillary analyses	18	Results of any other analyses performed, including subgroup analyses and adjusted analyses, distinguishing pre-specified from exploratory	
Harms	19	All important harms or unintended effects in each group (for specific guidance see CONSORT for harms)	
**Discussion**			
Limitations	20	Trial limitations, addressing sources of potential bias, imprecision, and, if relevant, multiplicity of analyses	
Generalisability	21	Generalisability (external validity, applicability) of the trial findings	
Interpretation	22	Interpretation consistent with results, balancing benefits and harms, and considering other relevant evidence	
**Other information**			
Registration	23	Registration number and name of trial registry	
Protocol	24	Where the full trial protocol can be accessed, if available	
Funding	25	Sources of funding and other support (such as supply of drugs), role of funders	

*We strongly recommend reading this statement in conjunction with the CONSORT 2010 Explanation and Elaboration for important clarifications on all the items. If relevant, we also recommend reading CONSORT extensions for cluster randomised trials, non-inferiority and equivalence trials, non-pharmacological treatments, herbal interventions, and pragmatic trials. Additional extensions are forthcoming: for those and for up to date references relevant to this checklist, see www.consort-statement.org.
