# Supplementary material for: Scheduled Intermittent Screening with Rapid Diagnostic Tests and Treatment with Dihydroartemisinin-Piperaquine versus Intermittent Preventive Therapy with Sulfadoxine-Pyrimethamine for Malaria in Pregnancy in Malawi: An Open-Label Randomized Controlled Trial
Source: PLoS Med. 2016 Sep 13;13(9):e1002124. doi: 10.1371/journal.pmed.1002124 (PMC5021271; doi:10.1371/journal.pmed.1002124)
Supplement: S3 Text — (PDF) [file pmed.1002124.s020.pdf]

Scheduled screening versus preventive treatment for the control of malaria in pregnancy in Malawi: a randomized controlled trial

Scientific Title: Scheduled intermittent screening and treatment in pregnancy (ISTp) versus intermittent preventive treatment with sulphadoxine-pyrimethamine (IPTp-SP) in women protected by insecticide treated nets (ITNs) for the control of malaria in pregnancy in Malawi: a randomized controlled trial

Study Acronym: ISTp-Malawi

Protocol Reference Number: COMREC: P. 07/10/955; LSTM REC: 10.74

Trial Registration:

Coordinator and Chief Investigator: Prof Feiko ter Kuile (email: terkuile@liverpool.ac.uk)

Principal Investigator: Dr Linda Kalilani-Phiri (email: lkalilani@medcol.mw)

Investigators: Mwayiwawo Madanitsa, Stephen Rogerson, Silke Lutzelschwab, Kara Hanson, Brian Faragher, Doreen Ali

Sponsor: Liverpool School of Tropical Medicine
Pembroke Place; Liverpool
L3 5QA; UK
serobert@liverpool.ac.uk
Tel: +44(0)151 705 3100; Fax: +44(0)151 705 3370

Funder: European and Developing Countries Clinical Trials Partnership (EDCTP)

Signatures:

Feiko ter Kuile

Sian Roberts (on behalf of sponsor)

Confidentiality statement: This document contains confidential information that must not be disclosed to anyone other than the sponsor, the investigational team, LSTM research office, Malawi CoM research support office, regulatory authorities, and members of the Research Ethics Committees and data monitoring committees.

1 Amendment History

AMENDMENT NO.	PROTOCOL VERSION NO.	DATE ISSUED	AUTHOR(S) OF CHANGES	DETAILS OF CHANGES MADE
1	2.01 v23mar11	23Mar11	Feiko ter Kuile	1. Primary endpoint to include SGA<br>2. Wording for use of source documentation amended<br>3. Amended details Interim analysis
2	3.01 v15Jun11	15Jun11	Feiko ter Kuile	1. Request for long term 10-year storage for future assays removed (as requested by NHRSC, Malawi).
3	4.01<br>v20Jul12	05Jul12	Feiko ter Kuile	1. Amended details economic study<br>2. Included Consent Form for exit survey as part of economic study (Appendix 3)

Contents

1 Amendment History 2
Contents 3
2 List of abbreviations 7
3 Synopsis 8
3.1 Diagram of main study visits 10
3.2 Summary of scheduled study visits 11
4 Background 12
4.1 Malaria in pregnancy 12
4.2 Intermittent preventive treatment in pregnancy with sulphadoxine-pyrimethamine (IPTp-SP) 12
4.3 Effectiveness of IPTp-SP in the context of increasing SP resistance 13
4.4 SP Resistance and declining effectiveness of IPTp-SP in Malawi 13
4.5 Economics of malaria in pregnancy 14
5 Rationale: Alternative approaches to IPTp-SP in the context of declining effectiveness ... 14
5.1 Alternative drugs for IPTp 15
5.2 Intermittent screening and treatment in pregnancy (ISTp) 15
5.2.1 RDTs for screening in ISTp 16
5.2.2 Dihydroartemisinin-piperaquine (DHA-PQ) for ISTp 16
6 Objectives 17
6.1 Primary objective 17
6.2 Secondary objectives 17
6.2.1 Efficacy 17
6.2.2 Tolerability and safety 18
6.2.3 Modifiers of efficacy 18
6.2.4 Effect on the development of pregnancy-specific immunity 18
6.2.5 Cost-effectiveness and economic burden 18
6.2.6 Acceptability, feasibility, implementability and scale up 18
7 Trial design 19
7.1 Overview of design 19
7.3 Rationale for design 20
7.2.1 Design as a superiority trial 20
7.2.2 Study of efficacy versus effectiveness 20
7.2.3 Open label design 21
7.2.4 Exclusion of HIV-positive women 21

7.2.5 Intervention schedules 21
7.2.6 Inclusion of women treated for malaria and anaemia 22
7.2.7 Additional malaria testing not part of the point of care testing and treatment 22
7.2.9 Stratified sampling of women with different gravidities 22
7.2.10 Use of a composite birth outcome as the primary trial endpoint 23
7.2.11 Assessment of adverse effects of treatment 23
8 Study populations 24
8.1 Inclusion criteria 24
8.2 Exclusion criteria 24
9 Outcome measures 25
9.1 Primary outcome 25
9.2 Secondary outcomes 25
9.2.1 Secondary efficacy outcomes 25
9.2.2 Safety outcomes 26
9.2.3 Tolerability outcomes 27
9.2.4 Immunology outcomes 27
9.2.4 Economic outcomes (sub-study) 27
10 Screening and eligibility assessment 28
11 Informed consent 29
12 Data management 30
13 Withdrawal or discontinuation of study participants 31
14 Definition of end of trial 31
15 Treatment of trial participants 31
15.1 The study intervention 31
15.1.1 Administration of the study drugs 32
15.2 Other treatment and concomitant medication 32
15.2.2 Unscheduled visits 33
15.2.3 Prohibited medication 33
16 Schedule of visits 33
16.1 Antenatal booking visit 33
16.1.1 Enrolment 34
16.1.2 Baseline assessment 34
16.1.3 Study intervention 34
16.1.4 Routine antenatal care and treatment of illness 35
16.1.5 Recording of morbidity and medication taken 35
16.2 Interim visit 35

16.2.1 Study intervention 35
16.2.2 Routine antenatal care and treatment of illness 35
16.2.3 Recording of morbidity and medication 35
16.3 Additional interim visit 35
16.4 Final antenatal visit 35
16.4.1 Study intervention 36
16.4.2 Routine antenatal care and treatment of illness 36
16.4.3 Recording of morbidity and medication 36
16.4.4 Additional study-specific testing 36
16.5 Home visits and telephone reminders (RDT-positive women treated with DHA-PQ only) 36
16.6 Two week post-treatment visit (RDT-positive women treated with DHA-PQ only) 37
16.7 Unscheduled visits during pregnancy 37
16.8 Delivery visit 37
16.8.1 Peripheral blood sampling 37
16.8.2 Placental sampling 37
16.8.3 Umbilical cord sampling 38
16.8.4 Examination of the baby 38
16.9 Seven-day postnatal visit 38
16.10 Six-week postnatal visit 38
16.11 Unscheduled postnatal visits 39
16.12 Follow-up of non-attenders 39
16.13 Data collection methods for economics and implementability sub-studies 39
17 Statistical methods 40
17.1 Sample size 40
17.2 Randomization 41
17.3 Analysis 41
17.3.1 Inclusion of participants in the analysis 41
17.3.2 Baseline comparability 42
17.3.3 Efficacy 42
17.3.4 Safety 43
17.3.5 Immunology 43
17.3.7 Handling of missing data 43
17.4 Criteria for early termination of the trial 43
17.5 CONSORT statement 43
17.6 Economics and implementability sub-study data analysis and management 44

18 Safety reporting.. 44
18.1 Definitions of adverse events.. 44
18.2 Reporting procedures for all adverse events.. 44
18.3 Reporting procedures for serious adverse events.. 45
19 Quality assurance procedures.. 46
20 Procedures for handling and accounting for study drugs.. 46
20.1 Labelling of study drugs... 46
20.2 Storage of study drugs and RDTs.. 46
20.3 Accountability for the study drugs... 47
21 Record keeping and archiving.. 47
22 Ethics.. 47
22.1 ICH Guidelines for Good Clinical Practice.. 47
22.2 Approvals.. 48
22.3 Informed consent... 48
22.4 Participant confidentiality.. 48
22.5 Other ethical considerations... 48
22.5.1 Safety of the study drugs in pregnancy... 48
22.5.2 Blood sampling... 48
22.5.3 Shipping and Storage of blood samples.. 49
22.5.4 Benefits to study participants.. 49
22.5.5 Inclusion of young people under the age of 18.. 49
22.5.6 Reimbursement of costs... 50
23 Financing and insurance... 50
24 Publication policy.. 50
25 References.. 51
Appendix 1: Definitions of adverse events.. 54
Adverse Event.. 54
Adverse Reaction... 54
Severe Adverse Event... 54
Serious Adverse Event or Serious Adverse Reaction (SAE/SAR).. 54
Suspected Expected Serious Adverse Reactions (SSAR).. 54
Suspected Unexpected Serious Adverse Reactions (SUSAR)... 54
Appendix 2: Participant information and consent forms... 55
Appendix 3: Participant information sheet economic study (exit survey among pregnant women at the antenatal clinic) and consent form.. 69

2 List of abbreviations

AE	Adverse event
AL	Artemether-lumefantrine
AQ-AS	Artesunate-amodiaquine
CoM	University of Malawi College of Medicine
CRF	Case record form
DHA-PQ	Dihydroartemisinin-piperaquine
EPI	Expanded programme on immunisation
FGD	Focus group discussion
Hb	Haemoglobin
HIV	Human immunodeficiency virus
HRP-2	Histidine-rich protein two
IPTp	Intermittent preventive treatment in pregnancy
IPTp-SP	Intermittent preventive treatment in pregnancy using sulphadoxine-pyrimethamine
ISTp	Intermittent screening and treatment in pregnancy
ISTp-DP	Intermittent screening and treatment in pregnancy, screening and treating malaria cases with dihydroartemisinin-piperaquine
ITN	Insecticide treated (bed) net
LMP	Last menstrual period
LSTM	Liverpool School of Tropical Medicine
MiP	Malaria in Pregnancy (Consortium)
MQ-AS	Mefloquine-artesunate
PI	Principal Investigator
PCR	Polymerase chain reaction
P. falciparum	*Plasmodium falciparum*
PfEMP1	*P. falciparum* erythrocyte membrane protein 1
pLDH	Plasmodium lactate dehydrogenase
RDT	Rapid diagnostic test (for malaria)
SAE	Serious adverse event
SP	Sulphadoxine-pyrimethamine
SUSAR	Suspected unexpected adverse reaction
VSA	Variant surface antigens
WHO	World Health Organization

3 Synopsis

**Study Title**	Scheduled intermittent screening and treatment in pregnancy (ISTp) versus intermittent preventive treatment with sulphadoxine-pyrimethamine (IPTp-SP) in women protected by insecticide treated nets (ITNs) for the control of malaria in pregnancy in Malawi: a randomized controlled trial<br><br>**Short title:**
**Public Title**	Scheduled screening versus preventive treatment for the control of malaria in pregnancy in Malawi: a randomized controlled trial
**Acronym**	ISTp-Malawi
**Protocol ref. no.**	COMREC: P. 07/10/955; LSTM REC: 10.74
**Clinical Phase**	IIIb (regulatory submission ongoing)
**Trial Design**	Two arm multi-centre randomized controlled superiority trial
**Trial Participants**	HIV-negative women in their second and third trimesters of pregnancy and attending for routine antenatal care at two sites in southern Malawi
**Planned Sample Size**	1,665
**Follow-up duration**	Up to six months during pregnancy, and six weeks after birth
**Planned Trial Period**	40 months
**Primary Objective and endpoint**	To compare the efficacy of scheduled intermittent screening with malaria rapid diagnostic tests (RDTs) and treatment of RDT-positive women with dihydroartemisinin-piperaquine (ISTp-DP) with intermittent preventive treatment with sulphadoxine-pyrimethamine (IPTp-SP) in the second and third trimesters on adverse birth outcome and malaria infection at term among HIV-negative women protected by insecticide–treated bed nets.<br><br>Endpoints: first and second pregnancies: A 25% or greater reduction in adverse birth outcome (composite of Small-for-gestational age (SGA), low birth weight or preterm birth); Multigravidae (G3+): Active or recent infection assessed at delivery by placental histopathology or RDT.
**Secondary Objectives and endpoints**	To determine if ISTp-DP has greater efficacy than IPTp-SP in terms of placental malaria (in G1 and G2), maternal malaria infection at delivery, mean birth weight, low birth weight (<2,500 grams), gestational age, mean gestational age at birth, pre-term birth (<37 weeks), small for gestational age, mean maternal haemoglobin at birth; anaemia (Hb ≤ 11 g/dL) at birth, moderate to severe anaemia (Hb ≤ 8g/dL); miscarriage, stillbirths; neonatal deaths; clinical malaria episodes during the second and third trimesters of pregnancy; third trimester mean maternal haemoglobin, anaemia (Hb ≤ 11 g/dL) and moderate to severe anaemia (Hb ≤ 8g/dL) and a compositve endpoint of the primary endpoint plus fetal loss; severe cutaneous skin reaction in the mothers; other serious

	adverse events in the mothers; minor adverse events in the mothers by day three after study drugs given; congenital malformation at birth and by day 28; neonatal jaundice at day one or day seven; incidence of anaemia, and clinical malaria in babies up to the age of eight weeks.<br><br>Immunological: To determine whether pregnancy-specific malaria immunity develops differently in women receiving IPTp compared to ISTp, and whether immunity levels at enrolment predict which women will respond best to IPTp or to ISTp, and have the best pregnancy outcomes.
**Sub-study Objectives**	Economic: To determine the cost-effectiveness of ISTp-DP versus IPTp-SP from a societal perspective and to use the cost data to populate a model of the economic burden of malaria in pregnancy. To model the economic cost of scale-up and affordability.<br><br>Acceptability and implementability: To explore the implementability, acceptability, feasibility and potential for scale-up of ISTp in Malawi.
**Investigational Products**	Dihydroartemisinin-piperaquine (DHA-PQ)
**Form**	Co-formulated tablets: 40 mg dihydroartemisinin and 320 mg piperaquine
**Dose**	2 mg dihydroartemisinin and 16 mg piperaquine per kg body weight per day for three days
**Route**	Oral, with water

3.1 Diagram of main study visits

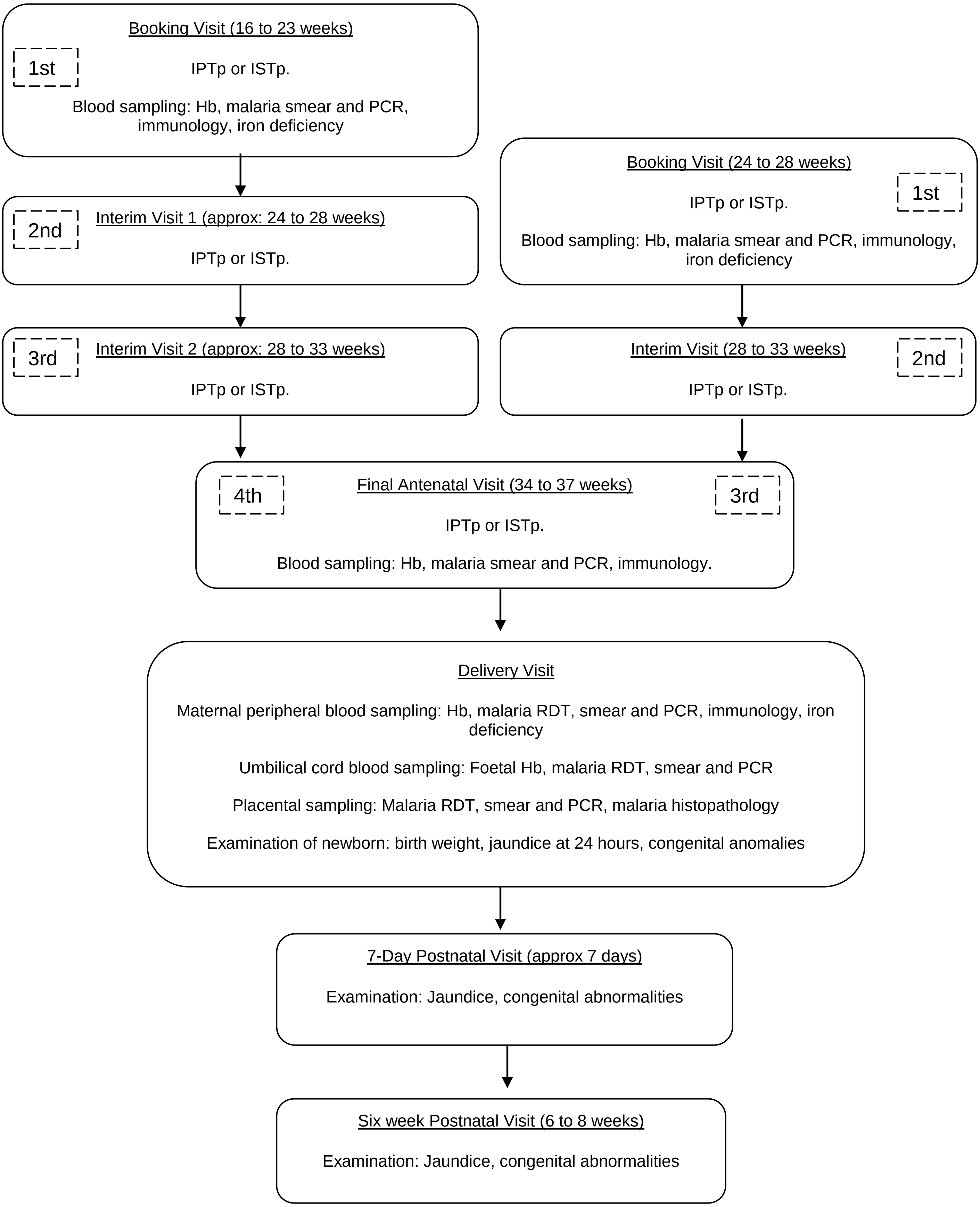

3.2 Summary of scheduled study visits

	**Booking visit** (16 to 28 weeks)	**Interim visit** (approx 24 -33 weeks)	**Additional Interim visit** (women enrolled before 24 weeks only)	**Final antenatal visit** (34 to 36 weeks)	**Telephone call for RDT+ women ISTp+ 2 days**	**Home visit for RDT+ women ISTp+ 2 days**	**Clinic visit for RDT+ women ISTp+ 14 days**	**Delivery**	**7-day postnatal visit** (7 days)	**6-week postnatal visit** (6 -8 weeks)
**Actions**										
Eligibility confirmed	X									
Consent	X									
Randomization	X									
IPTp or ISTp	X	X	X	X						
**Measures**										
Adherence monitoring					*Sub-sample	*Sub-sample				
Tolerability/ adverse events		X	X	X	*Sub-sample	*Sub-sample				
Blood biochemistry							*Sub-sample			
Full blood count							*Sub-sample			
Blood drug levels							*Sub-sample			
Immunology	X			X				X		
Peripheral malaria microscopy and PCR	X			X				X		
Peripheral malaria RDT								X		
Hb and anaemia**	X**			X**						
Placental microscopy, RDT and PCR								X		
Placental histopathology								X		
Umbilical cord blood RDT, microscopy and PCR								X		
Birth weight								X		
Gestational age								X		
Congenital anomalies								X	X	X
Neonatal jaundice								X	X	
Blood sample for storage	X			X				X		
HIV**				X**						
Blood markers of malaria immunity and iron deficiency	X							X		

*Women in the ISTp-DP group who were RDT-positive and treated with DHA-PQ

**Routine care

An ultrasound scan will be done at screening, enrolment, or within a month of enrolment.

4 Background

4.1 Malaria in pregnancy

Pregnancy increases a woman's susceptibility to malaria infection, with those living in rural areas, in their first or second pregnancy, infected with HIV, and adolescents at particularly high risk. In endemic countries with stable malaria transmission, such as Malawi and much of sub-Saharan Africa, most malaria infections in pregnant women remain asymptomatic or have only mild symptoms, and are therefore undetected and untreated. Symptomatic or asymptomatic falciparum malaria infection affects the placenta and is associated with unfavourable outcomes for both mother and baby, including maternal anaemia, low birth weight (due to intra-uterine growth retardation or pre-term birth) and an increased risk of neonatal death.[1] There is also increasing evidence that babies born to mothers with placental malaria infection are at increased risk of malaria infection in their first and second years of life, [2, 3, 4, 5], and anaemia[6, 7, 8]. Placental malaria has also been associated with reduced mother to child transfer of protective antibodies to infectious diseases such as measles.[9] The control of malaria in pregnancy is therefore important for the health of mothers and babies and an important element of antenatal care in endemic areas. The World Health Organization (WHO) currently recommends three strategies to control malaria in pregnancy; case-management of symptomatic malaria; providing insecticide-treated bed nets (ITNs) to reduce exposure to infective mosquito bites; and intermittent preventive treatment of malaria in pregnancy (IPTp).

4.2 Intermittent preventive treatment in pregnancy with sulphadoxine-pyrimethamine (IPTp-SP)

Intermittent preventive treatment in pregnancy (IPTp) is currently recommended for HIV-negative women in all areas with stable moderate to high transmission of malaria.[10] The strategy consists of administration of treatment doses of an efficacious antimalarial drug at predefined intervals at least a month apart during the second and third trimesters of pregnancy, regardless of the presence of malaria parasitaemia. The most common policy is to provide two doses as part of routine antenatal care, but some countries provide three doses[10]. The strategy is thought to work by providing intermittent clearance or suppression of parasites in the placenta, and preventing new infections from occurring.

Sulphadoxine-pyrimethamine (SP) is the only drug currently used for IPTp. It has a profile that makes it highly suitable for this use. SP is very well tolerated and safe in the second and third trimester of pregnancy for mother and foetus[11]. SP is also widely available, cheap, and can be given as a single dose.

IPTp-SP is not recommended for HIV-positive women. WHO recommends that HIV-positive women receive co-trimoxazole prophylaxis for HIV-related infections.[12] Because co-trimoxazole and SP are both sulpha-containing drugs, SP is contra-indicated in HIV-positive women receiving co-trimoxazole. However, co-trimoxazole itself has some antimalarial properties and therefore serves to protect pregnant women with HIV infection from malaria infection.[12]

4.3 Effectiveness of IPTp-SP in the context of increasing SP resistance

Increasing SP resistance is now a major challenge to IPTp-SP efficacy and effectiveness. Because of widespread and increasing resistance to SP, almost all countries in eastern and southern Africa have now switched their first-line treatment for symptomatic malaria to an artemisinin based combination therapy (ACT), as recommended by WHO.[13] A previous meta-analysis showed that IPTp with SP remains effective even in areas where SP fails in approximately one third of treatments of symptomatic children with malaria by day 28.[11] SP therefore continues to be used for IPTp in many countries where it is no longer used for treatment of symptomatic malaria, because it has maintained efficacy for this purpose.

However, there is now evidence from a recent study in northern Tanzania that IPTp-SP may no longer be effective at higher rates of resistance, and may even be harmful in the context of very high resistance.[14] In this study IPTp use was associated with increased (rather than decreased) placental inflammation and levels of parasitaemia in malaria-infected pregnant women carrying parasites with the SP resistance mutation *dhps* 581. This supports a hypothesis that the most highly resistant parasites out-compete by having a relative fitness advantage and can overgrow less resistant parasites under drug pressure. The use of SP for IPTp in settings of widespread high SP resistance may therefore actually exacerbate malaria infections and further increase SP resistance.

4.4 SP Resistance and declining effectiveness of IPTp-SP in Malawi

Malawi was the first country to introduce IPTp-SP in 1993, and the strategy initially appeared effective. Monitoring by the Queen Elizabeth Central Hospital in Blantyre showed that between 1997 and 2006 the prevalence of placental malaria detectable by microscopy decreased five-fold from 21.2% to 4.2% in first pregnancies and ten-fold from 18.9% to 1.9% in second and subsequent pregnancies.[15] At the same time there was a steady increase in ITN use from 18% to 60%; while IPTp-SP use increased from around 20% in 1997 to 78% in 2002, but dropped again to less than 50% by 2006, suggesting that the increased use of ITNs was responsible for the continued fall of placental malaria rates.[15]

The same study observed that the effectiveness of IPTp-SP in Malawi appeared to reduce dramatically between 2002 and 2006, to the extent that it may no longer be protective. After 2002, women who received two or more doses of IPTp-SP were equally likely to have placental malaria, anaemia, or low birth weight babies compared to women who had not received any IPTp. During the same period, ITNs were strongly associated with higher birth weights and decreased infection rates.

These finding are consistent with an increasing impact of SP resistance on IPTp effectiveness. The degree of SP resistance in 2001-2004 was high; over 90% of parasites had the 'quintuple mutant' (*dhfr* mutations N511, C59R and S108N, and *dhps* mutations A437G and K540E) associated with SP resistance.[16] However, previous trials have also shown that IPTp-SP provides less benefit in women who are also protected by ITNs[17]; therefore the reduced efficacy of SP probably reflects the combination of high concurrent ITN use and high levels of SP resistance.

More recent observational studies of the impact of SP resistance on IPTp-effectiveness have been cause of further concern about the longevity of SP, with further evidence that at the current levels of resistance (year 2010), SP appears to provide no benefit to pregnant women protected by ITNs delivering at the Queen Elizabeth Central Hospital and two rural clinics in Blantyre district (Kalilani and ter Kuile, personal communication). Preliminary analysis of the in-vivo follow-up module showed that by June 2010, 43 of 87 (49.4%) asymptomatic parasitaemic women receiving SP in their first or second pregnancy were parasitaemic again between days 7 and 42, compared to only 6 of 72 (8.3%) women in their third or subsequent pregnancies. Rates of placental malaria also seem to have increased since 2006; Between Jan-Jun 2010 (n=390) 25% of primi and secundi gravid women had positive placental RDTs or histology smears and this was 33% when recent infections where included (active or past infection assessed by placental histopathology) despite having received the full course of IPTp-SP, and this was 20.5% among multigravidae. At delivery pregnant women were equally to be parasitaemia when they had received the full course of SP or no SP at all (Relative risk 0.99) (Kalilani and ter Kuile, personal communication).

4.5 Economics of malaria in pregnancy

To date only limited data are available on the economic impact of malaria in pregnancy. To capture adequately the economic burden of malaria in pregnancy and conduct a high quality cost-effectiveness analysis, good epidemiological data, including the effects on the mother and offspring, the costs associated with prevention and treatment, and a better understanding of the long-term health and economic consequences are needed.[18, 19]

To date there are three published cost-effectiveness analyses on malaria in pregnancy interventions in Malawi,[20, 21, 22] with two comparing presumptive treatment with chloroquine versus SP and one comparing different health education messages to improve compliance to chemoprophylaxis. None of them took any costs incurred by households into consideration.

A review conducted by Worrall et al found IPTp-SP to be relatively cost-effective or highly cost-effective in all studies conducted in sub-Saharan Africa,[19] but this is likely to be affected by the falling transmission, by the coverage of ITNs and by the increasing resistance of *P. falciparum* to SP. In the context of scarce resources it is important to assess the cost-effectiveness, affordability, feasibility and implementability of the proposed intervention in addition to its efficacy and effectiveness.

5 Rationale: Alternative approaches to IPTp-SP in the context of declining effectiveness

The problem of declining IPTp effectiveness now urgently needs to be investigated and studies of alternative approaches to IPTp-SP considered. The two possible options for consideration are replacing SP with other drugs for IPTp, and alternative strategies to replace IPTp.

5.1 Alternative drugs for IPTp

A series of studies is looking into alternative drugs that could replace SP for ITPp. A study in Benin showed mefloquine to be more efficacious than SP for preventing placental malaria, clinical malaria, and maternal anaemia at delivery.[23] A study in Ghana showed IPTp with amodiaquine or a combination of amodiaquine and SP to have comparable effects to SP.[24] Further studies comparing IPTp-SP and IPTp using mefloquine (MiP Consortium) and with azythromycin-chloroquine[25] are ongoing in Africa and the results will be available in the next three to four years. None of these new IPTp candidates, however, have the same favourable profile as SP; they are more complicated to give (azithromycin-chloroquine and amodiaquine-based regimens need to be taken once daily for three days, compared with a single dose for SP) and are not as well tolerated, which are important considerations in a strategy that targets otherwise healthy asymptomatic women.

Furthermore, malaria transmission among pregnant women has reduced considerably in southern Malawi[15] and is likely to decline further following increased control measures including indoor residual spraying (IRS), widespread ITNs and use of ACTs in the community (ACTs kill gametocytes and can therefore help reduce transmission). This reduces the number of women at risk of malaria and the potential impact and cost-effectiveness of presumptive approaches such as IPTp. The marked reduction in exposure risk and the need for change to an alternative drug regimen has changed the risk-benefit profile for IPTp. Under these circumstances, intermittent screening and treatment in pregnancy (ISTp) provides a potential alternative strategy that is increasingly being considered.

5.2 Intermittent screening and treatment in pregnancy (ISTp)

The concept of ISTp is to provide scheduled screening for malaria using a rapid diagnostic test (RDT) and treating RDT-positive women with a long acting ACT, with the aim of clearing existing infections and providing additional post-treatment prophylaxis for three to six weeks. Screening ensures that only women who test positive for malaria parasites receive treatment, and women without evidence of malaria are not unnecessarily exposed to antimalarial drugs. ISTp is delivered as part of 'focused antenatal care'.[26] Women are thus screened and potentially treated at least three times during the second and third trimesters of pregnancy.

There is now some evidence that ISTp may be an effective strategy for some parts of Africa[27]. In a study of 3,333 pregnancies in Ghana, three scheduled screening visits and treatment of RDT-positive women with amodiaquine-artesunate was found to be equally as effective in reducing clinical malaria attacks, parasitaemia at 36 to 40 weeks pregnancy, maternal anaemia, and low birth weight as routine IPTp-SP in areas with good SP sensitivity. Importantly, ISTp was also well received by pregnant women and was manageable within a busy antenatal clinic.[28]

There is now increasing interest in ISTp as a potential future strategy to replace IPTp-SP in areas where either SP is failing as IPTp or in areas with marked reduction in malaria transmission. The MiP Consortium is funding two further multi-site trials looking at ISTp. One trial will be conducted in West Africa in four countries with low SP resistance and declining

transmission and will compare IPTp-SP with ISTp using artemether-lumefantrine; with the primary endpoints being low birth weight, third trimester anaemia and placental malaria.29 A similar trial, co-ordinated by the MiP Consortium, will be undertaken in Jharkand state, India and will compare ISTp with SP-artesunate with passive case detection of malaria (Chandramohan, personal communication).

5.2.1 RDTs for screening in ISTp

RDTs are used in preference to microscopy for screening in ISTp because they are simple to use; potentially more sensitive at detecting placental malaria than expert microscopy; and allow for a much faster point of care diagnosis than microscopy, especially in busy clinics. The increased sensitivity of the HRP-2 based RDTs, in comparison to microscopy is due to their ability to detect antigens released from parasites in the placenta into the blood stream, even if peripheral parasitaemia is not present.[30, 31] In a study of women giving birth in Ghana, the sensitivity of microscopy of peripheral blood smear to detect placental malaria was 42%, compared with 80% for an HRP-2-based RDT, compared with PCR.[30] The corresponding specificities were 97% for microscopy and 90% for RDT. The lower specificity for RDTs compared with microscopy may have been partly due to the persistence of HRP-2 antigens in the blood, which may remain for up to 14 days after clearance of parasites following treatment.

5.2.2 Dihydroartemisinin-piperaquine (DHA-PQ) for ISTp

We will use DHA-PQ as the drug of choice for ISTp. DHA-PQ is an artemisinin combination therapy (ACT) drug combination, each co-formulated tablet consisting of 40 mg dihydroartemisinin and 320 mg piperaquine. WHO now recommends ACTs for the treatment of symptomatic malaria in the second and third trimesters of pregnancy, and many countries are in the process of changing their first line drug in the second and third trimesters of pregnancy from quinine to ACTs, as quinine needs to be given three times daily for seven days and is poorly tolerated.

Dr Kalilani et al are currently conducting a multicentre trial in Malawi, Burkina Faso, Ghana and Zambia, assessing the efficacy of different ACTs for the treatment of malaria in women who have a positive malaria smear in the second and third trimesters of pregnancy; ‘Safe and efficacious artemisinin-based combination treatments for African pregnant women with malaria’ (COMREC reference number P03/09/737). The four ACTs being assessed in this trial, approved by the Malawi Pharmacy and Poison Board in May 2010, are dihydroartemisinin-piperaquine (DHA-PQ), artemether-lumefantrine (AL), artesunate-amodiaquine (AQ-AS) and mefloquine-artesunate (MQ-AS). The study has a total sample size of 3,480 participants, including 870 in the DHA-PQ arm (260 of whom are scheduled to receive DHA-PQ in Malawi). Of these regimens, DHA-PQ is the most promising candidate for use in ISTp, despite the possibly longer period post-treatment prophylaxis offered by MQ-AS[32], because previous trials have shown that it is better tolerated than AQ-AS or MQ-AS, can be given once daily (unlike AL), and provides longer post-treatment prophylaxis than AL or AQ-AS by one to two weeks after each dose[33]. Because ISTp will be given up to three times, this could result in a cumulative difference of three to six weeks post-treatment prophylaxis.

Treatment trials in non-pregnant adults and children have shown DHA-PQ to be highly effective and well tolerated.[34] Minor adverse events associated with DHA-PQ included nausea, vomiting, loss of appetite, diarrhoea, abdominal pain, headache, dizziness, and sleep disturbance.[34] A case series study in Thailand using DHA-PQ to treat pregnant women after treatment failure with quinine suggested that DHA-PQ was effective in pregnancy at the standard adult dose,[35] and larger-scale trials in the same area are now ongoing (Nosten, personal communications). There is also significant experience with DHA-PQ in the second and third trimester from southern Papua, Indonesia, where the drug is used as first line treatment in the second and third trimesters of pregnancy and has been shown to be very effective and well tolerated in pregnancy (Price, personal communications).

Animal studies have shown artemisinins to be embryotoxic and potentially teratogenic in very early pregnancy in all animal species tested, but studies in humans, including over 1,000 documented pregnancies, have shown no adverse effects on the mother or foetus when taken in the second or third trimester of pregnancy.[13] Reproductive toxicity studies in Australia (Davis, unpublished observations) and more recently by Sigma Tau (Medicine for Malaria Venture, personal communication) have not raised safety concerns with piperaquine. A lengthening in the duration of labour in rat models has been observed (Medicine for Malaria Venture, personal communication), but this has not been confirmed in humans in Thailand (Nosten, personal communications).

The Good Manufacturing Practice (GMP) compliant product of DHA-PQ, manufactured as Eurartesim by Sigma-Tau (Italy), is in the phase-III stage of development and under consideration by the European Medicine Agency (EMEA). Approval is anticipated in 2011, after which it is expected that several countries in sub-Saharan Africa may consider switching their first line treatment of malaria in the second and third trimesters of pregnancy to DHA-PQ.

6 Objectives

6.1 Primary objective

To compare the efficacy of scheduled intermittent screening with malaria rapid diagnostic tests (RDTs) and treatment of RDT-positive women with dihydroartemisinin-piperaquine (ISTp-DP) with intermittent preventive treatment with sulphadoxine-pyrimethamine (IPTp-SP) in the second and third trimesters on adverse birth outcome and malaria infection at term among HIV-negative women protected by insecticide–treated bed nets.

6.2 Secondary objectives

6.2.1 Efficacy

To determine if ISTp-DP compared with IPTp-SP in the second and third trimesters of pregnancy is associated with:

- A decrease in the prevalence of placental malaria at delivery
- An increase in the mean duration of pregnancy or a reduction in the prevalence of pre-term birth

- An increase in mean birth weight or a reduction in the prevalence of low birth weight
- A decrease in stillbirths, neonatal deaths or perinatal death
- A decrease in pregnancy loss in the second and third trimesters
- An increase in mean haemoglobin levels or decrease in any anaemia, moderate anaemia and severe anaemia at delivery
- A decrease in the prevalence of peripheral parasitaemia at delivery
- A decrease in the incidence of clinical malaria episodes in pregnancy
- A decrease in the incidence of peripheral parasitaemia in the third trimester of pregnancy
- An increase in mean haemoglobin levels or decrease in any anaemia, moderate anaemia and severe anaemia during the third trimester of pregnancy
- A decrease in the prevalence and incidence of clinical malaria or symptomatic anaemia in infants by seven days and six to eight weeks

6.2.2 Tolerability and safety

To evaluate the tolerability and safety of ISTp-DP in the second and third trimesters of pregnancy.

6.2.3 Modifiers of efficacy

To explore the possibility of differences in treatment effect by gravidity (first or second pregnancy versus third or subsequent pregnancies) or other groupings relating to the risks of malaria in pregnancy, such as malaria transmission season, location, age, number of intervention visits.

6.2.4 Effect on the development of pregnancy-specific immunity

To explore the relative effect of the two different study interventions on markers for pregnancy-specific immunity to *P. falciparum* malaria.

6.2.5 Cost-effectiveness and economic burden

- To estimate the cost-effectiveness of ISTp-DP versus IPTp-SP from a societal perspective measuring both provider and user costs
- To use the cost data to populate a model of the economic burden of malaria in pregnancy
- To model the economic cost of scale up of ISTp-DP in Malawi and explore the affordability of the scale up

6.2.6 Acceptability, feasibility, implementability and scale up

To explore the acceptability, feasibility, implementability and potential for scale up of ISTp-DP in a busy, routine antenatal clinic setting, using a qualitative assessment.

7 Trial design

7.1 Overview of design

This will be a two-arm randomized controlled trial conducted at three or more sites in southern Malawi with high levels of SP resistance and high ITN coverage. Enrolment will be stratified by gravidity group (stratified design); one for primi and secundigravidae (G1+2) and one for multigravidae (G3+). The study requires 1665 women overall, including 1155 primi and secundigravidae and 500 multigravidae. It is designed to detect at least a 25% reduction in the primary endpoint consisting of a composite of small-for-gestational age (SGA), low birth weight or preterm birth in women in their first or second pregnancies, and at least a 50% decrease in placental malaria in women in multigravidae. The study will be open label as it will not be possible to blind the participants to their allocation, although where possible laboratory staff undertaking trial-related diagnostic tests will be blinded.

Antenatal clinic sites at Mpemba, Madziabango and Zingwangwa and Chileka will be involved. If less than half the required number of participants is recruited within the first half of the recruitment period, antenatal sites in neighbouring clinics in Blantyre, Zomba or Chikawa districts may be added.

Participants will be HIV-negative pregnant women. They will be screened for eligibility and given information about the study at their first contact with antenatal services before 28 weeks gestation and will be enrolled at 16 to 28 weeks gestation. The study is expected to start in the fourth quarter of 2010 or first quarter of 2011 and recruit for a period of 18 to 30 months, with a further six months follow-up.

Participants will be randomly allocated (see section 17.2 for description of the randomization process) to receive either at least three doses of IPTp with SP or at least three scheduled screenings with an RDT and treatment with DHA-PQ if they are RDT-positive. All participants will be given an insecticide-treated bed net if they do not already have one.

Women enrolled in the trial will make at least three scheduled visits to the clinic spread over the second and third trimesters at least four weeks apart to receive the study intervention approximately mirroring the appointment schedule for ‘focussed antenatal care’ in Malawi which consists of four scheduled visits. Dating ultrasound scans will be performed to assess gestational age. Women attending early in the second trimester will be asked to make four scheduled visits four to eight weeks apart.

Participants will receive all usual antenatal care according to local policy, including standard clinical examinations, tests and necessary treatment. Women will also be encouraged to attend the study clinic for assessment if they feel unwell during the study period, and to deliver in the maternity wards of the study sites to facilitate collection of delivery outcome data. Participants who deliver at home will receive a home visit within 72 hours (maximum time a week), if possible, to collect the same data as collected for facility births. Participants receiving DHA-PQ will also receive a home visit on day two after the first dose of treatment to encourage and assess their adherence to the study medication, and assess tolerability. Newborns will be seen at approximately seven days and six weeks after delivery, to assess the health of the infant.

In addition to the delivery and birth outcomes, and passive and routine surveillance for illness during pregnancy, blood samples will be taken from all women during the third trimester of pregnancy for later malaria testing by microscopy and PCR. Haemoglobin levels will be tested again in women in the third trimester, and those found to be anaemic will be treated with haematinics according to Government guidelines.

Effects of the different interventions on the development of pregnancy-specific immunity will be evaluated to understand the potential impact of ISTp on development of antibody immunity to malaria, and how this might affect the natural development of gravidity dependent protection from malaria and its complications.

Alongside the main trial will be a study of the feasibility and economics if ISTp. Health facility and exit surveys will be carried out to estimate the costs of the intervention to the health services and households. To capture the costs incurred during the six weeks after delivery, questions about the use of health services will be integrated into the clinical health assessment at six weeks.

Participants will be reimbursed for travel costs to and from the clinic and receive a food allowance. If possible, a study driver will take them home on the day of enrolment to ascertain and record the exact location of their home and address. Women will also be asked for their mobile or home telephone number, if they have one. Attempts will be made to contact women who do not attend scheduled appointments, and to encourage them to attend or to ascertain their reasons for non-attendance.

7.3 Rationale for design

7.2.1 Design as a superiority trial

ISTp will be more complex to implement and potentially more expensive than IPTp and, unlike with IPTp, sub-patent infections will not be prevented or suppressed, thus concomitant use of ITNs is important. Countries with moderate transmission would only consider switching to a new strategy if it is clearly superior to the current policy of IPTp-SP, either because SP is failing or because SP is potentially harmful.

7.2.2 Study of efficacy versus effectiveness

This trial is designed primarily to assess the efficacy of ISTp when implemented as intended. This proof of concept is needed as a logical step before further assessments of effectiveness in practice are undertaken, as it has not yet been established in this context. In addition, as any effect will usually tend to be greater under conditions of optimal implementation, the sample size required is smaller for an efficacy trial than for an effectiveness trial. Therefore efforts will be made to maximise participant adherence to the study protocol, including telephone reminders to women to take their DHA-PQ as prescribed, and active follow-up of participants who miss scheduled appointments.

However, some attempts will be made to assess the potential effectiveness of ISTp in practice. Data will be collected to assess the tolerability of the intervention and levels of adherence under the trial conditions.

7.2.3 Open label design

Participants will be informed of the intervention they are assigned to, since the processes for IPTp and ISTp are different, and blinding is not therefore possible. Blinding will be undertaken only for diagnostic tests performed by laboratory personnel.

7.2.4 Exclusion of HIV-positive women

Women who are HIV-positive or refuse an HIV test will not be included in this trial because all HIV-positive women in Malawi are eligible to receive co-trimoxazole prophylaxis of HIV-related infections, as recommended by WHO. Co-trimoxazole itself provides some protection from malaria infection, and SP is contra-indicated in women receiving co-trimoxazole. It would not be possible to include HIV-positive women in the current trial, as they cannot be included in the IPTp-SP arm. The prevalence of HIV in pregnancy women in Malawi is around 12%. It is therefore important to evaluate the potential effectiveness of ISTp in HIV-positive women, but this would require a different trial answering a different question, comparing co-trimoxazole prophylaxis alone with co-trimoxazole prophylaxis plus ISTp. At the clinics involved in this trial, HIV testing is offered routinely, and less than 1% of women refuse the test.

7.2.5 Intervention schedules

The study intervention will be delivered at intervals of four to eight weeks and as far as possible corresponding to the usual schedule of 'focused' antenatal care visits during the second and third trimesters of pregnancy which are scheduled at approximately 24-28 weeks, 30-32 weeks, and 34-36 weeks. Women attending between 16 and 23 weeks may receive an additional RDT screening or SP dose, as with IPTp this is what tends to happen in practice in Malawi. The last intervention in both the ISTp group and IPTp group will be at approximately 34 to 37 weeks of gestation, to ensure clearance of the placenta before delivery. As per usual standard of care, women who have not delivered by 38 weeks will be seen weekly until the birth, but these will not be classed as study visits, and no study intervention will be given.

The regimen for IPTp in Malawi used to be at least two doses of SP during the second and third trimesters of pregnancy. However, more recently Malawi has switched to a more pragmatic approach providing SP at each scheduled visit as part of focussed antenatal care, and many women now receive three or four doses of SP. Similar regimens have been adopted by Kenya and Zambia. Furthermore, there is recent compelling evidence that three doses of SP iare more effective than two doses at reducing the incidence of low birth weight in trials conducted in Kenya[36], Malawi[37] (Luntamo, personal communication) and Mali (Maiga, personal communication). The summary relative risk of low birth weight in four trials comparing SP given twice with SP three times or monthly was 0.67 (95% CI 0.49 to 0.91, p=0.01) in favour of the three-dose group (Kayentao and ter Kuile, personal communication) suggesting that the three-dose IPTp-SP resulted in a 33% additional reduction in low birth weight compared with two-dose IPTp-SP. Two of these four trials were conducted in Malawi. The most likely explanation for this large added benefit is the high rates of re-infection occurring in the second half of the third trimester, a period of rapid foetal weight gain.

As this trial is designed to determine the superiority of a new, more expensive and complex intervention (ISTp) to current practice (IPTp), it is important that the current intervention is implemented in its most effective form possible. Therefore we plan to give at least three doses of SP to women in the IPTp group, consistent with current practice in Malawi. This will have the additional benefit within the trial of ensuring that participants in the IPTp and ISTp arms receive the same number of scheduled antenatal visits in the second and third trimesters of pregnancy, thus ensuring that they receive the same care in pregnancy, with the exception of the study intervention.

7.2.6 Inclusion of women treated for malaria and anaemia

Women with symptoms of malaria at enrolment will not be excluded, but will be randomized to the study groups, and treated with the case-management drug, artemether-lumefantrine (CoArtem) if they fall in the IPTp-SP group. They will not receive SP that month, because lumefantrine is anticipated to provide post-treatment prophylaxis comparable with SP (3 to 4 weeks), based on recently completed treatment studies in pregnant women in Uganda. SP will then be provided at the next scheduled visit. Women randomized to the ISTp arm will already receive DHA-PQ as the treatment drug. DHA-PQ and CoArtem have comparable efficacy in treating malaria.

Women participating in the trial will receive all usual routine antenatal care, and will also be encouraged to attend the clinic if they feel unwell between appointments. As part of this package of care, women attending with symptoms consistent with malaria or anaemia will be tested and treated as appropriate, and continue in the study. No exclusions will be made relating to testing or treatment for these conditions, as the same care will be available equally to women in both study arms, and the same care would be provided if either intervention were introduced into usual practice.

7.2.7 Additional malaria testing not part of the point of care testing and treatment

All participants who give consent will have a blood sample taken at enrolment and during the third trimester to test for peripheral malaria using standard microscopy of blood smears and PCR. The results of these tests will not become available until the end of the trial and will not be used to inform the care of the women. In this way, a point prevalence of malaria at baseline and in the third trimester can be estimated for both intervention arms, without contaminating the intervention in either arm. Testing of asymptomatic pregnant women is not usual practice in Malawi; the treatment of asymptomatic malaria in pregnancy has not yet been shown to be advantageous in women receiving IPTp; and women in the ISTp group will receive malaria testing and treatment if positive in any case; therefore no existing intervention will be withheld. This blood sample will be combined with a routine sample for haemoglobin in the third trimester, and with a second HIV screening test following the new WHO guidelines for repeat HIV testing in the third trimester.[38]

7.2.9 Stratified sampling of women with different gravidities

We anticipate most or all of the impact on birth outcomes and morbidity to be in women in their first and second pregnancies, based on systematic review of previous trials with IPTp-SP and ITNs.11, 39 However, the impact on placental malaria infection is anticipated to

occur across all pregnant women, including those in their third and subsequent pregnancies. Placental malaria and malaria at term are now recognised to be important endpoints in themselves as they have been found to be risk factors for malaria, anaemia and other infections in infants up to the age of two years, independent of the effect of malaria on gestational age low birth weight. The effect appears to be particularly evidence in multigravidae. Furthermore, IPTp is currently recommended for and provided to women of all gravidities, and it is anticipated that any alternative strategies to IPTp may also be provided to all pregnant women, if any potential benefit is identified. This trial will therefore focus on morbidity endpoints in women in their first and second pregnancies, and on malaria infection at term and delivery in women in their third to fifth pregnancies (active or past infection). They will be enrolled using a stratified sampling strategy.

7.2.10 Use of a composite birth outcome as the primary trial endpoint

In women in the first and second pregnancies, this trial aims primarily to assess the effect of the intervention on birth outcomes. A composite primary outcome of SGA, low birth weight or pre-term birth will be used because there will be more births falling into one of these categories than into a single category and malaria during pregnancy is associated with increased risk of all these three measures, including an increase in the risk of SGA in children born at term and with birth weigth > 2500 g; Therefore a smaller sample size will be needed to detect a significant difference between study intervention arms than if a single outcome were used. The use of a composite outcome has the disadvantage that all events are given equal weight; however, SGA, low birth weight and pre-term births will also be reported separately as secondary analyses.

Early neonatal deaths are not included within the composite endpoint measure because the aetiological fraction of deaths due to malaria is anticipated to be small; most early postnatal deaths result from events occurring during delivery (e.g. prolonged labour).

7.2.11 Assessment of adverse effects of treatment

The safety profile of SP in pregnant women is already well documented, and this trial has very limited potential to add significantly to that body of knowledge. There has been less research into the safety of DHA-PQ in pregnancy. In this trial, too few women will receive DHA-PQ to undertake a meaningful analysis of sub-clinical effect, as DHA-PQ will only be provided to women enrolled in the ISTp arm who have a positive RDT result. However, blood samples will be taken at 14 days after treatment and the following tests undertaken to add to the body of knowledge: full blood count; blood haemoglobin levels; total bilirubin, alanine aminotransferase and creatine; and blood DHA-PQ levels. These tests will not be point of care.

Interim placental histopathology results from another trial (IPT-mon) suggest that around 28% (95% CI 19% to 42%) of pregnant women in southern Malawi may experience placental malaria infection. Around 776 participants in this trial will receive ISTp; and since only 20% to 30% of those are likely to be treated with DHA-PQ (as the rest will be RDT-negative throughout) only around 155 to 233 will receive the drug. A larger trial multi-centre study is currently ongoing to assess the safety, efficacy and tolerability of different ACT regimens for the treatment of smear-positive malaria in pregnant women in Malawi and three other African countries; 'Safe and efficacious artemisinin-based combination treatments for African

pregnant women with malaria'. This trial will include 860 women scheduled to receive DHA-PQ and laboratory testing for sub-clinical effects will be undertaken. Data from this trial will add to that knowledge base.

Although minor events will be monitored in the ISTp arm, because of difficulties in identifying appropriate comparisons, there will be no systematic comparison of minor adverse event rates between trial arms. At each study intervention visit, participants will fall into three different categories: women with or without malaria infection taking SP; RDT-positive women taking DHA-PQ; and RDT-negative women taking no drug. The three groups will be different not only in the drug taken, but in their malaria status, knowledge of their malaria status, and their knowledge of the drug taken. Unbiased comparisons between groups are therefore not possible. Follow-up for symptomatic adverse events will therefore focus on serious adverse events, adverse events serious enough to be reported to the study clinics through passive surveillance or requiring treatment, and the tolerability of DHA-PQ assessed by women's self-reports on day three after the first dose.

8 Study populations

8.1 Inclusion criteria

1. Viable singleton pregnancy
2. Gestational age 16 to 28 weeks (inclusive) by LMP (if available) or fundal height
3. No history of IPTp use during this pregnancy
4. Willing to participate and complete the study schedule
5. Has provided written informed consent
6. Resident of study area and intending to stay in the area for the duration of the follow-up
7. Willing to deliver in the labour ward of the study clinic or hospital

8.2 Exclusion criteria

1. HIV positive or unknown HIV status
2. Multiple gestations
3. High risk pregnancy resulting in referral to tertiary delivery facilities according to local guidelines, these include:
    a. Age under 16 or over 40
    b. Five or more previous pregnancies
    c. Pre-existing illness likely to cause complication of pregnancy (hypertension, diabetes, asthma, epilepsy, renal disease, fistula repair, leg or spine deformity)
    d. Two or more previous miscarriages or induced abortions
    e. Previous caesarian section delivery, vacuum extraction or symphysiotomy
    f. History of pregnancy or birth complications (pre-eclampsia or post-partum haemorrhage)
4. Severe anaemia requiring blood transfusion (Hb ≤ 7.0 g/dL) at enrolment
5. Known allergy or previous adverse reaction to any of the study drugs
6. Unable to give informed consent (for example due to mental disability)
7. Previous inclusion in the same study

9 Outcome measures

9.1 Primary outcome

In women in their first or second pregnancy: composite endpoint of adverse birth outcomes, defined as any of:

- Small for gestational age defined as a binary outcome of <10th percentile of fetal weight for attained gestational age using the new Landis fetal weight nomogram from the Democratic republic of Congo.[40]
- Preterm birth (spontaneous birth before 37 weeks gestation)
- Low-birth-weight (birth weight under 2,500 grams)

In women in their third to fifth pregnancies: Malaria infection at term and delivery will be the primary endpoint, defined as evidence of current or recent infection assessed at delivery by placental histopathology ('active' or 'past' infection) or RDT (pLDH or HRP2 positive, any species) or PCR positive (any species).

9.2 Secondary outcomes

9.2.1 Secondary efficacy outcomes

1. Placental malaria (any species):
    a. Past infection detected by histopathology
    b. Active infection detected by:
        i. Histopathology
        ii. Microscopy
        iii. Rapid diagnostic test
        iv. Polymerase chain reaction (PCR)
2. Maternal malaria infection (peripheral blood) at delivery, detected by:
    a. Microscopy
    b. RDT
    c. PCR
3. Peripheral malaria infection during pregnancy detected by:
    a. Microscopy
    b. PCR
4. Birth weight
    a. Mean birth weight (grams)
    b. Low birth weight (<2,500 grams)
5. Gestational age
    a. Mean gestational age at birth (grams)
    b. Pre-term birth (<37 weeks)
6. Small for gestational age
7. Maternal haemoglobin and anaemia:
    a. At delivery
        i. Mean maternal haemoglobin (g/dL)
        ii. Anaemia (Hb ≤ 11 g/dL)
        iii. Moderate to severe anaemia (Hb ≤ 8g/dL)

b. During third trimester
    i. Mean maternal haemoglobin (g/dL)
    ii. Anaemia (Hb ≤ 11 g/dL)
    iii. Moderate to severe anaemia (Hb ≤ 8g/dL)
8. Miscarriage (loss of foetus before 28 weeks gestation)
9. Stillbirth (birth at 28 weeks or later showing no signs of life)
10. Composite endpoint of the primary endpoint plus fetal loss (miscarriage or stillbirths)
11. Infant death
    a. Perinatal death (stillbirth or death within 7 days of birth)
    b. Neonatal death (death within 28 days of birth)
12. Malaria infection of the newborn, detected by analysis of umbilical cord blood with:
    a. RDT
    b. Microscopy
    c. PCR
13. Foetal haemoglobin and anaemia by sampling of umbilical cord blood at birth:
    a. Mean foetal haemoglobin (g/dL)
    b. Foetal anaemia (Hb ≤ 12.5 g/dL)
    c. Moderate to severe foetal anaemia
14. Incidence of documented clinical malaria episodes during the second and third trimesters of pregnancy (history of fever in last 24 hours and documented malaria microscopy or RDT positive)
15. Presence of any evidence of malaria infection at term (last antenatal visit), identified through microscopy or PCR, or at delivery, identified through peripheral and placental RDT, microscopy or PCR, or placental histopathology (active or past infection).
16. Incidence of other illness episodes apparent at scheduled antenatal clinic visits or resulting in unscheduled clinic visits
17. Incidence and prevalence of clinical malaria in infants by seven days and six to eight weeks determined by:
    a. RDT
    b. Microscopy
    c. PCR
18. Prevalence of symptomatic infant anaemia at seven days and six to eight weeks
    a. Anaemia
    b. Moderate to severe anaemia
19. Incidence of other illness episodes in the infants, apparent at scheduled postnatal clinic visits or resulting in unscheduled postnatal clinic visits

9.2.2 Safety outcomes

1. Severe cutaneous skin reaction in the mothers within 30 days of drug intake
2. Other serious adverse events in the mothers
3. Congenital malformations identified by six weeks after birth
4. Neonatal jaundice within 24 hours and at seven days
5. Laboratory test results outside of normal range

9.2.3 Tolerability outcomes

1. Non-serious adverse events in the mothers
2. Adherence to study medication

9.2.4 Immunology outcomes

Concentration of antibodies known to be associated with protection against malaria in pregnancy and in general, including antibodies recognizing variant surface antigens on *P. falciparum* infected erythrocytes that block parasite adhesion to chondroitin sulphate A.

9.2.4 Economic outcomes (sub-study)

The economics sub study will be conducted alongside the main clinical trial. Health facility and exit surveys will be carried out to estimate the costs of the intervention to the health services and households respectively. To capture costs incurred during the first six to eight weeks after delivery, including the costs of caring for low birth weight babies, questions about use of health services will be integrated into the clinical health assessment at six weeks.

The specific outcomes of the economics sub-study are:

1. Costs of the two intervention arms to the health facility and household up to six to eight weeks after delivery. Household data will be collected by questionnaire at the six-week clinic visit; health facility data directly from the health facility.
2. Cost-effectiveness of ISTp-DP versus IPTp-SP measured in terms of cost per each of the following endpoints averted, which are measured in the main trial:

- Adverse birth outcome (still birth, preterm birth or low birth weight)
- Active and past malaria infection of the placenta (detected by histopathology, microscopy or RDT)
- Maternal anaemia
- Peripheral malaria at delivery
- Neonatal deaths

Cost per disability adjusted life year (DALY) averted will be estimated using the cost data collected and effectiveness data generated by the trial, with necessary adjustments made to the DALY to accommodate outcomes in pregnant women and their newborns.

The household and facility data from the cost and cost-effectiveness analysis will be used (alongside data from other studies) to populate a model of the economic burden of malaria in pregnancy. In addition, they will be used to:

- Model the long-term costs and consequences of malaria in pregnancy to the household and health facility in both trial arms, and to
- Model the costs of scaling up the intervention at regional/national level and investigate affordability in Malawi.

9.2.5 Acceptability, feasibility, implementability and scale up: outcomes

The overall aim of this sub study is to explore the acceptability, feasibility, implementability and potential for scale-up of ISTp-DP. The main outcomes are more specifically:

- Social, cultural and economic determinants of demand, access and use for malaria in pregnancy interventions.
- Acceptability of ISTp for provider and user.
- Preferences for malaria in pregnancy interventions at the user and provider level.
- Factors at facility and district levels which influence the delivery of malaria in pregnancy interventions and in particular the feasibility and implementability of ISTp in the context of other reproductive health interventions (e.g. prevention of mother to child transmission).
- Major barriers to the scale-up and use of interventions to control malaria in pregnancy, specifically of ISTp.

10 Screening and eligibility assessment

Potential participants will be screened for eligibility when attending their first routine antenatal appointment. If they meet the inclusion criteria they will be offered enrolment following informed consent. Women who do not meet the inclusion criteria, or who do not wish to participate in the trial, will receive usual antenatal care, including the standard IPTp-SP schedule.

Eligibility screening will be conducted by clinic nurses and midwives and supported by study staff. Screening data will be recorded in a log that will be kept in the investigators site file. One screening log will be kept per site. This record will be used to report how many women attended for antenatal appointments, how many were potentially eligible, how many were enrolled into the study, and reasons for non-eligibility of those not enrolled. It will be used to ensure that women are selected for the study without bias. Each screened woman will be assigned a screening registration number in sequential order by the study clinic. Screened attendees who do not meet the eligibility criteria for the trial will be considered screening failures, in which case the registration number will not be re-used; however the woman can re-enter the study at a later date if she meets the eligibility criteria at that visit.

Gestational age will initially be estimated first using a combination of fundal height as measured by the midwife and last menstrual period (LMP) dates provided by the woman, where available. Women attending antenatal care for the first time after 28 weeks gestation will be excluded from the trial. Women attending before 16 weeks gestation will be screened for inclusion, given information about the trial and enrolled when they next attend between 16 and 28 weeks gestation. Women who are eligible and express an interest in the trial may receive an ultrasound scan at this point, using portable equipment to accurately assess the gestational age of the pregnancy and identify multiple gestations. Alternatively the scan may be done at another appointment shortly after enrolment. The ultrasound scan will be done by a trained clinician who is part of the study team. Any women identified as having multiple gestations will be excluded from the study. Ultrasound scans are not part of routine antenatal care in Malawi; the equipment will be specially purchased and study staff trained to use it.

Women already known to be HIV-positive will be automatically excluded from the study. All women will be offered HIV counselling and testing using rapid diagnostic tests, and if they refuse or are found to be positive will be excluded from the trial and offered co-trimoxazole

prophylaxis. Refusal to be tested is very rare at these clinics (<1%). For women presenting before 16 weeks gestation, HIV testing will be performed as part of usually care before the woman is invited to join the study, using a finger-prick blood sample. For women presenting between 16 and 28 weeks, HIV testing will be undertaken using a venous blood sample that will be used for both routine and study-specific tests. As venous sampling would not normally be included as part of care, consent for screening would be obtained before this blood draw.

All potentially eligible women will be tested for anaemia. If they are found to be severely anaemic (Hb ≤ 7 g/dL) they will be excluded from the trial and referred to the nearest hospital for blood transfusion. If a woman with severe anaemia is successfully treated and attends the clinic again before 28 weeks gestation, she will become eligible for the trial provided that she meets all other inclusion criteria. Women with less severe anaemia will be included in the study and their anaemia treated by treating any cause identified and with ferrous sulphate 200-400mg twice daily for 2-3 months.

Women will be asked whether they plan to travel or migrate outside of the study area (to be defined for each site before the start of the trial) during the follow-up period. Women who plan to travel outside of the study area will be excluded.

Women who are found to be ineligible after they have signed their informed consent will be excluded from the trial but will continue to receive all study-specific benefits, if she wishes to (e.g. missed twin pregnancies).

11 Informed consent

The consent information will be delivered by the study nurse at the participating antenatal clinics, in a consulting room. Participants will have as long as they like to consider the information, although if they first present at 28 weeks gestation they will need to decide whether or not to participate during that week, as the study is integrated into the usual schedule of antenatal visits. Women who decide to join the study may withdraw at any time.

There will be three elements to consent for involvement; consent for eligibility screening; consent for inclusion in the trial; and consent for storage of blood samples.

Consent for screening will be sought from all women who appear potentially eligible at the pre-screening stage, and will be specific to procedures that are not normally undertaken as part of routine care. This includes a 5ml venous blood draw taken before enrolment, which will be used to assess the woman for anaemia, and, if the test has not already been undertaken, HIV and syphilis, as well as for study-specific tests if the women is found eligible. It may also include an ultrasound scan, if available at this stage.

Informed consent for inclusion in the trial will be sought immediately before enrolment after 16 weeks gestation. Women who make first contact before 16 weeks and who are potentially eligible will receive all relevant information at this time, and will be asked for consent to screening, but will not be asked for their formal written consent for participation in the trial until their next attendance after 16 weeks.

Each potential participant will be given full and adequate oral and written information about the study, including all the known risks and any potential benefits, in their own local language. It will be clearly stated that the participant is free to withdraw from the study at any

time for any reason without prejudice to future care, and with no obligation to give the reason for withdrawal. Participants will be given the opportunity to ask questions and allowed time to consider the information provided.

Informed consent will be documented on a written consent form signed by the participant and the person who conducted the informed consent discussion. If the participant is unable to write her signature then a thumbprint may be used. If the subject is unable to read the information herself, full and comprehensive verbal information will be communicated in the presence of a witness; who will be independent of the conduct of the trial. The witness will sign the consent form to attest that information in the consent form was accurately explained and apparently understood by the participants, and that informed consent was freely given.

Participants will be asked for additional consent for the transport of blood sample outside of the country and the storage of blood samples for future research, and this consent will be recorded separately. Women who do not consent to the storage and transport of blood, or who withdraw their consent, can still participate in the trial.

Pregnant women aged 16 years or older are considered emancipated in Malawi and are able to sign the consent form for themselves; no parental consent is required for emancipated minors according to Malawian law.

Either carbon copies or otherwise two identical consent forms will be used per women; one signed or thumb-printed consent form will be kept on file by the study team and one given to the participant.

12 Data management

Source documents will be the participants' antenatal cards, delivery and birth records, laboratory results, and clinic records. All study-relevant data will be transferred from the source documents to the case record forms (CRFs). In addition, some study specific data will be recorded directly onto the CRF. Each participant will have their own document file containing some of their original documents (e.g. print-out of automated laboratory results) and the CRF. The Principal Investigator will ensure that the CRFs are accurate, complete and legible.

The data recorded on the CRF will be input into an electronic database, managed by the data manager under the local PI in Malawi. Data validation and verification will be done to ensure that the data in the database corresponds with the CRFs and source documents. Data query sheets will be raised and distributed by the data manager to the study team for resolution in a timely manner. An anonymized, encrypted copy of the database will be kept at LSTM.

At the end of the trial and when the validation process is concluded and once all decisions on the evaluability of the data from each individual participant have been made and documented, the database will be locked.

All documents will be stored safely in confidential conditions. On all study-specific documents, other than the signed consent forms, the participant will be referred to by their unique study participant number and initials, not by name.

13 Withdrawal or discontinuation of study participants

Participants will only be withdrawn from the study for the following reasons:

- Withdrawal of consent by the participant. Participants may choose to withdraw at any stage.
- Screening failure. The participant did not meet the required inclusion or exclusion criteria, and was included in the study in error
- Loss to follow-up. The participant moved out of the study area, or was lost to follow-up for some other reason.
- Death of the study participant.
- Safety reasons as judged by the safety monitor, investigator or sponsor.

A participant who chooses to withdraw from the study will always be asked for her reasons and about the occurrence of any adverse events and the reasons for her withdrawal will be recorded on the CRF.

Participants who discontinue the study drugs will not be considered withdrawn from the trial. With the participant's consent, every effort will be made to follow up those who discontinue the study drug due to adverse events in order to determine the final outcomes.

Non-adherence to the study schedule will not lead to the withdrawal of the participant unless it indicates a significant risk to her safety.

Participants who discontinue the study prematurely will not be replaced.

14 Definition of end of trial

The end of trial will be defined as the final assessment of the last infant born within the trial, unless additional time is needed for follow-up of a trial-associated adverse event.

15 Treatment of trial participants

15.1 The study intervention

Participants will receive one of the following two interventions during the second and third trimesters of pregnancy; provided at each of three scheduled visits between four and six weeks apart.

IPTp-SP Group: Treatment with a three tablets of sulphadoxine-pyrimethamine, each containing sulphadoxine (500 mg) and pyrimethamine (25 mg). This is the standard and only

drug of choice for IPTp in Africa. SP will be purchased from Durbin PLC in the UK and will be manufactured according the GMP: Durbin PLC is a UK registered company - Company Registration Number: 3626868; Head Office: 180 Northolt Road, South Harrow, Middlesex, HA2 0LT, UK T: +44 (0)20 8869 6500, F: +44 (0)20 8869 6565, info@durbin.co.uk

ISTp-DP Group: Screening for malaria using a combined HRP-2/ pLDH (*P. falciparum/* pan-malaria) rapid diagnostic test (First Response® Malaria pLDH/HRP2 Combo Test, target antigen pLDH (pan); HRP2; Premier Medical Corporation Ltd, USA), and treatment if RDT-positive with dihydroartemisinin-piperaquine (Sigma Tau). Each tablet will contain 40 mg dihydroartemisinin and 320 mg piperaquine. Treatment will be given for three days, with the daily number of tablets depending on the weight of the woman to the nearest half tablet; dosage being 2 mg/kg/day of dihydroartemisinin and 16 mg/kg/day piperaquine.

15.1.1 Administration of the study drugs

Women in either group who attend for a scheduled study appointment with fever and malaria confirmed by RDT or microscopy will be treated for malaria. In the case of women in the IPTp-SP group, case-management treatment will be given in place of the scheduled dose of SP, and this will be recorded on the CRF.

RDTs will be performed according to the manufacturer's instructions by the study staff at the clinic. Women in the ISTp group testing positive for either *P. falciparum* or other malaria species will be treated with DHA-PQ.

Participants receiving single-dose SP or the first dose of DHA-PQ will be given some clean water for swallowing the tablets. They will be observed swallowing the tablets and for thirty minutes afterwards. If vomiting occurs during this time the full dose will be repeated. If the participant vomits again after the repeated dose she will be discontinued from the trial and provided with second line therapy for malaria (CoArtem).

Participants receiving treatment with DHA-PQ will be given the remaining doses to take home, with instructions to take half the tablets the following day and half tablets the day after. Women with mobile or home telephones will be telephoned the next day to remind them to take their next dose. Women without mobile phone will be visited at home. All women regardless of mobile phone ownership will be visited by a fieldworker on the second day to check whether they have taken the tablets and whether they have had any problems.

15.2 Other treatment and concomitant medication

15.2.1. Routine care

All participants will be offered routine antenatal care according to local policy and the principles of 'focussed antenatal care'. This includes the provision of insecticide-treated bed nets (ITNs) free of charge.

Routine antenatal care in Malawi includes blood screening at the first appointment for syphilis, anaemia and HIV, and the provision of appropriate treatment for these conditions. Women diagnosed with anaemia or syphilis will be treated as appropriate. Women diagnosed before or during the trial as HIV-positive will be excluded or withdrawn from the

trial and offered co-trimoxazole prophylaxis. Blood screening for anaemia and HIV will also be repeated in the third trimester, as per usual care in Malawi (anaemia) and per new WHO guidelines (HIV).

As part of their antenatal care participants will be given iron and folic acid supplements (folic acid dose 0.4 mg to 0.6 mg / day) and tetanus vaccination if applicable. Helminth infections will be treated with praziquantel 40 mg / kg single dose for schistosomiasis infection, albendazole 400 mg / kg single dose for hookworm, trichuriasis and ascariasis, and albendazole 400 mg / kg per day for three days for strongyloidasis.

15.2.2 Unscheduled visits

Participants will be encouraged to attend the study clinics if they feel unwell, and will also be asked about their use of self-medications at each of their clinic appointments. All concomitant medications prescribed to participants during the study, or reported as used by the participants, will be recorded in the appropriate sections of the CRF with the indication, dose, and dates of administration.

Women diagnosed with symptomatic uncomplicated malaria during the course of the study will be treated with artemether-lumefantrine (CoArtem) as first line and with oral quinine as second line if they become symptomatic and parasitaemic again within 4 weeks. The exception is women in the ISTp group who are attending their first or other routine appointments, who will receive DHA-PQ if RDT-positive, with CoArtem reserved as second line therapy. Women with severe malaria will be admitted to hospital for treatment with intravenous quinine.

Study infants who become unwell at birth or during the follow-up period will also receive all appropriate healthcare as usually provided according to local policy and guidelines. Infants with severe jaundice will be treated with phototherapy according to national guidelines. Where umbilical cord blood is found to be positive for malaria by RDT at birth, microscopy smear of umbilical cord blood will be prioritised for the next 24 hours and, if positive, the baby will be treated for malaria according to national guidelines.

15.2.3 Prohibited medication

Prohibited medication includes antimalarial drugs not prescribed within the trial protocol, and other drugs with antimalarial properties including co-trimoxazole. Participants who take prohibited medications will remain in the trial and will be included in the primary, intention to treat analysis, but excluded from the per-protocol analysis.

16 Schedule of visits

16.1 Antenatal booking visit

If a woman makes first contact with the study team between 16 and 28 weeks gestation, the first study visit will immediately follow eligibility screening. If a woman makes first contact before 16 weeks gestation it will be conducted at their next appointment between 16 and 28

weeks gestation. Informed consent will be obtained at this visit after completion of eligibility screening and before enrolment.

16.1.1 Enrolment

After informed consent has been signed, participants will be randomly allocated (see section 17.2) to receive either IPTp or ISTp (see section 15.1) and issued with a trial identity card with their unique study identification number. Participants will be informed of study intervention they have been allocated to.

At this stage study staff will ask the participant for a home or mobile telephone number if they have one, and home address or a description of the location of their home, and for their verbal consent to be visited at home two days after receiving the study drugs (if they receive DHA-PQ) and whenever they do not attend scheduled appointments.

16.1.2 Baseline assessment

A baseline assessment will be conducted for each participant comprising demographic information, socioeconomic information, insecticide treated net (ITN) and indoor residual spraying use, medical and obstetric history, and clinical assessment. Any relevant information already collected during the screening process or at previous antenatal appointments for the same pregnancy will be copied from the antenatal cards onto the CRF, so that questions, tests and examinations are not unnecessarily duplicated.

Study-relevant information recorded at the first visit will include age; area of residence; prior use of ITNs; number of previous known pregnancies and number of previous births. Clinical assessment will include height and weight, fundal height, reported date of last menstrual period and estimated gestation.

A 5 ml venous blood sample will be taken and used for both routine and study-specific testing. This will be obtained after consent for eligibility screening has been obtained but before consent for enrolment in the trial; women who have not been tested for HIV in their current pregnancy will be tested for HIV, and all women will be tested for anaemia and haemoglobin levels. Women not previously tested for syphilis in this pregnancy will be tested and treated if positive. The same blood sample will be prepared for later study-specific testing for malaria (blood smear microscopy and PCR), for markers of immunity to *P. falciparum* malaria, and for markers of iron deficiency. Aliquots of separated blood plasma will be stored at -20 or -80C for immunity studies. A filter-paper sample will be prepared and stored for future research if the woman has given her consent for this.

If not already done, an ultrasound scan will be done at this visit or at a separate visit within a month of enrolment.

16.1.3 Study intervention

The first dose of IPTp-SP or RDT screening for ISTp-DP will be administered to asymptomatic women at this visit as described in section 15.1. If women have symptomatic malaria they will be treated as described in section 15.2.

16.1.4 Routine antenatal care and treatment of illness

Routine antenatal care and treatment of any illness identified at this visit will be provided as described in section 15.2. This will include the provision of a suitable ITN if the woman does not already have one, and advice on sleeping under the net for the duration of the pregnancy.

16.1.5 Recording of morbidity and medication taken

All participants will be asked about any symptoms or illnesses they have had in the last month, and any medication taken.

16.2 Interim visit

A second clinic visit will be scheduled between four and eight weeks after the first visit.

16.2.1 Study intervention

The second dose of IPTp-SP or RDT screening for ISTp-DP will be administered to asymptomatic women at this visit as described in section 15.1. If women have symptomatic malaria they will be treated as described in section 15.2.

16.2.2 Routine antenatal care and treatment of illness

Routine antenatal care and treatment of any illness identified at this visit will be provided as described in section 15.2.

16.2.3 Recording of morbidity and medication

Participants will be asked about any symptoms or illnesses they have had since the first antenatal visit of the study, and any medication taken.

16.3 Additional interim visit

This is an additional visit only for participants who were enrolled in the trial at between 16 and 23 weeks gestation and for whom four doses of the study intervention can be provided at intervals of four to eight weeks apart, with the final visit at 34 to 37 weeks. This visit will be conducted in the same way as the other interim visit.

16.4 Final antenatal visit

A clinic visit will be scheduled between 34 and 37 weeks gestation, at least four weeks after the previous visit.

16.4.1 Study intervention

The third or fourth dose of IPTp-SP or RDT screening for ISTp-DP will be administered at this last scheduled visit as described in section 15.1. If women have symptomatic malaria they will be treated as described in section 15.2

16.4.2 Routine antenatal care and treatment of illness

Routine antenatal care and treatment of any illness identified at this visit will be provided as described in section 15.2.

As part of routine care, all women will have their haemoglobin levels tested at point of care. Women found to be anaemic (Hb ≤ 11 µg/ L) will be treated as appropriate. All women will also be re-tested for HIV, as recommended in the latest WHO guidelines.[38] This will be obtained for a single venous blood draw with additional study-specific testing as described in section 16.4.4.

16.4.3 Recording of morbidity and medication

Participants will be asked about any symptoms or illnesses they have had since the first antenatal visit of the study, and any medication taken.

16.4.4 Additional study-specific testing

All consenting participants will have venous blood samples taken, prepared and stored for later malaria testing using standard microscopy of blood smears and PCR of dried blood spots. Testing will be done blindly and results will not be made available to study staff until the end of the trial.

16.5 Home visits and telephone reminders (RDT-positive women treated with DHA-PQ only)

Participants who receive DHA-PQ treatment as part of ISTp and who have a mobile or home telephone number will be telephoned on days two and three after the first dose to remind them to take the second and third doses, and will otherwise visited at home to ensure compliance.

All participants who receive DHA-PQ treatment as part of ISTp will be visited at home day three after receiving their first dose of DHA-PQ at the clinic.

At this visit, participants will be asked whether they have taken the tablets assigned for the previous day and the current day. If they have not taken the tablets, they will be asked for their reasons, and if willing, will be encouraged to take the remaining tablets during the visit. They will also be asked about any symptoms they have experienced on the day of the visit and two previous days. If the symptoms are serious adverse events, a 2 ml venous blood sample will be taken to measure blood drug levels.

Any cases of non-adherence to the study medication and the reasons given for will be recorded in the CRF.

16.6 Two week post-treatment visit (RDT-positive women treated with DHA-PQ only)

Women treated with DHA-PQ will be asked to return to the clinic 14 days after the first dose is given. A venous blood sample will be taken for testing of blood DHA-PQ levels, full blood count (red blood cell count, white blood cell count, differential blood cell count); blood haemoglobin levels; and biochemistry (bilirubin, alanine aminotransferase and creatine) doe later analysis.

16.7 Unscheduled visits during pregnancy

Participants will be encouraged to visit the clinic if they feel unwell between scheduled appointments.

Participants who present between appointments will be examined by study staff. Presenting symptoms, axillary temperature and blood pressure will be recorded. A finger-prick blood sample will be taken for Hb measurement and malaria RDT and thick and thin microscopy smears. Women presenting with serious adverse events possibly associated with DHA-PQ will have a 2 ml venous blood sample taken to measure blood drug levels. Any illness will be treated as appropriate and according to standard local care. The date of attendance, diagnosis and treatment will be recorded on the CRF, and any adverse events reported according to standard procedures.

16.8 Delivery visit

Women will be encouraged to deliver on the maternity wards of the participating clinics, in which case they will be assisted to delivery by regular clinic midwives, and a specially trained member of the study team will visit them on the ward and perform relevant examinations shortly after delivery. Women who deliver at home will be identified using a network of community nurses and traditional birth attendants, and will be visited by a member of the study staff within 48 hours, to collect, as far as possible, the same information.

16.8.1 Peripheral blood sampling

A 5 ml venous blood sample will be taken from the mother and tested for malaria using RDT, standard microscopy and PCR; and tested for Hb concentration using hemocue. Women found to have malaria infection by RDT or microscopy, or anaemia (Hb ≤ 11 g/L) will be treated as appropriate. This sample will also be used to test for markers for general and pregnancy-specific immunity to malaria, and for markers of SP resistance and iron deficiency.

16.8.2 Placental sampling

A blood sample will be collected from the delivered placenta by making an incision on the maternal side and collecting the pooled blood. This sample will be tested for malaria using RDT, standard microscopy of blood smear and PCR.

A 2cm x 2cm x 1cm specimen of placental tissue will be taken from the maternal side for histopathology testing for current and past malarial infection.

16.8.3 Umbilical cord sampling

An umbilical cord blood sample will be taken and tested for Hb and malaria using RDT, standard microscopy of blood smear and PCR. If the RDT is positive for malaria, microscopy will be prioritised for within the next 24 hours and if positive by microscopy the baby will be referred to the hospital for treatment of malaria.

16.8.4 Examination of the baby

The baby will be weighed using accurate digital scales, and will be examined for vital status, presence of any significant congenital anomalies, and gestational age at birth using the Ballard's score. After 24 hours, the baby will be re-examined for the presence of jaundice. Examinations will be done by specially trained study nurses or by the study physician.

16.9 Seven-day postnatal visit

Participants and their babies will be seen at the clinic by a study physician around seven days after birth, if possible to correspond with the baby's first vaccination visit. The baby will be examined for the presence of jaundice and for any congenital anomalies that may have been missed at delivery. Babies with symptoms of malaria will have blood samples taken for testing by RDT, microscopy and PCR; treatment will be provided based on the RDT results. Standard postnatal advice and healthcare will be provided as needed for mother and baby, and any treatment and clinical findings for the baby recorded on the CRF. Routine postnatal care will be provided for the women. Women who do not attend for this appointment will be visited at home if possible. Any infant deaths occurring before the visit will be recorded; the date and probable cause of death will be ascertained by verbal autopsy.

16.10 Six-week postnatal visit

Participants and their babies will be seen again at the clinic by a study physician around six to eight weeks after birth, at their baby's second vaccination visit. The baby will be examined for the presence of congenital anomalies that may have been missed at delivery or the first visit. Babies with symptoms of malaria will have heel-prick blood samples taken for testing by RDT, microscopy and PCR; treatment will be provided based on the RDT results. Standard postnatal advice and healthcare will be provided as needed for mother and baby, and any treatment and clinical findings for the baby recorded on the CRF. Women who do not attend the scheduled appointment will be visited at home if possible. Any infant deaths occurring before the visit will be recorded; the date and probable cause of death will be ascertained by verbal autopsy. Participants will also be asked about their use of healthcare for the baby since birth.

16.11 Unscheduled postnatal visits

Women will be encouraged to attend the clinic if their baby becomes unwell during the follow-up period. Babies will be examined by study staff. Presenting symptoms, axillary temperature and blood pressure will be recorded. A heel-prick blood sample will be taken for Hb measurement and malaria RDT and thick and thin microscopy smears. Any illness will be treated as appropriate and according to standard local care. The date of attendance, diagnosis and treatment will be recorded on the CRF, and any adverse events reported according to standard procedures. Clinical care will also be provided for women who attend because of their own illness, but this will not be recorded as part of the study.

16.12 Follow-up of non-attenders

Participants who do not attend scheduled appointments will be contacted by a member of study staff by telephone, if the participant has provided a contact telephone number. If participants cannot be contacted by telephone, or if they still do not attend when reminded, they will be visited at home within two weeks of the missed appointment by community-based Health Surveillance Assistants and invited to re-attend, or if they no longer wish to participate in the trial their reasons recorded.

16.13 Data collection methods for economics and implementability sub-studies

Health facility costs and intervention costs will be collected through observational studies, key informant interviews and sight of relevant financial documents. The following cost data will be collected:

- Materials and drugs: SP, DHA-PQ, diagnostic tests, treatment of pregnant women with symptomatic malaria, treatment of maternal anaemia, treatment of infant anaemia and treatment for low birth weight infants. It may be necessary to collect treatment costs outside of the trial facility, so that the costs of ‘actual practice’ rather than ‘trial practice’ can be estimated. Cost data will be collected through review of accounting documents and interviews with key informants drawn from facility management (e.g. accountant, health facility in-charge, department managers).
- Staff time: The interventions will be provided within the antenatal care programme of the Ministry of Health of Malawi. The incremental staff time of the two interventions will be estimated through a combination of interviews with health workers and observation.

Out of pocket costs associated with pregnancy care and with the prevention and treatment of malaria during pregnancy will be estimated using exit surveys at antenatal care clinics (ANCs). Women will be asked about how many times they have attended the ANC during their current pregnancy and about health tests, care and medical attention they have received. They will be asked about malaria prevention tools they may have been given at the ANC or that they may have bought or received somewhere else. They will be asked about health related costs incurred during their current pregnancy. In order to be able to estimate their social status, women will be

asked about their family, occupation, sources of income, their houses and assets owned.

In order to allow comparison, we propose to include both women who experienced at least one episode of malaria and women who did not experience any episode of malaria during their current pregnancy in the study. Only those women who will sign informed content forms will be administered the questionnaire and included in the study. The participant information sheet and consent form are presented in Appendix 3. 385 pregnant women who experienced malaria and 385 pregnant women who did not experience malaria will be interviewed.

Costs of caring for low birth weight infants will be collected in health assessments six to eight weeks after delivery.

Contextual information will be collected using focus group discussions (FGDs), community in-depth interviews and stakeholder interviews.

Acceptability will be studied using FGDs, semi-structured interviews, in-depth interviews, observations and a discrete choice experiment.

Feasibility and implementability will be explored using survey techniques, FGDs, semi-structured interviews and in depth-interviews.

17 Statistical methods

17.1 Sample size

The trial will use a stratified design with one strata for primigravidae and secundigravidae and one strata for multigravidae. It is designed to detect a 25% reduction (RR 0.75) in composite adverse birth outcomes (SGA, low birth weight and pre-term birth) in women in their first and second pregnancies with an 90% power and a 2-sided significance level of 0.05, if the rate of placental malaria in the IPT-SP group is similar to that found ongoing observational studies of women receiving routine care in the same area of Malawi. The proposed difference is based on four previous trials of IPTp which jointly reduced low birth weight by 29%.[11] Thus the efficacy of ISTp is expected to be similar to the efficacy of IPTp before the emergence of widespread SP resistance. In the ongoing IPT-mon study in Malawi, the incidence of SGA, low birth weight or preterm birth was 40.3% (N=494) (Kalilani, personal communication). Therefore the study is designed to detect a 25% difference between 40.3% in the control (IPTp) group and 30.2% in the intervention (ISTp) group.

This would require 491 participants in each arm or 982 in total. To allow for a 15% loss to follow-up, 1155 women in their first and second pregnancies will be recruited. This sample size would also have an 81% power to detect a 25% or greater reduction in placental malaria (active or past infection), with a significance level of 0.05. If a power of 80% is used, the study can detect a difference of 21.8% or greater in the primary endpoint. If the incidence is 33.3% instead of 40.3% the study still has 80% power (instead of 90%) to detect a 25% reduction.

The trial is also designed to detect a 50% reduction in past or present placental malaria infection at delivery in multigravidae, based on previous studies of IPTp,[11] assessed by placental histopathology and RDT. The current rate in women in Malawi who are taking IPTp-SP is estimated from IPT-mon at 20.5% (Kalilani, personal communication). A reduction by 50% to 10.25% will require 213 women per arm or 426 in total. To allow for a 15% loss to follow-up we will recruit 500 multigravidae.

The total sample size will therefore be 1155 + 500 = 1655.

17.2 Randomization

Two randomization sequences will be computer-generated by the study statistician at Liverpool School of Tropical Medicine, one for women in their first and second pregnancies and another for multigravidae. The method used for each will be block randomization, stratified by study site to ensure an equal proportion of participants in each intervention group from each site. The length of each block will vary and will ensure that allocation concealment is fully maintained and allocation well distributed over the seasons. The allocation ratio for the two study arms will be 1:1.

Recruitment will be 'competitive' between study sites, meaning that the number of participants recruited at each site may differ depending on the respective rates of enrolment. A sufficient number of randomization codes will be generated for each site to allow for this.

The randomization sequences will be sent to the study sites in Malawi and local investigators will prepare opaque envelopes, numbered sequentially, with the allocated group code and details for that number inside. For each newly enrolled participant, an envelope from the correct sequence (according to gravidity group) will be opened sequentially to identify the group that they are allocated to, thus concealing the upcoming allocation from the participants, clinic staff and study staff. The number on the envelope represents the study number allocated to that participant and will become their unique identifier in the trial. This number will be recorded with their screening registration number on their CRF, and the participants provided with a trial identity card confirming their study number. Once a study number has been assigned to a participant it will not be used again, for example if a participant discontinues. If a study number is allocated incorrectly, it will not be reassigned and randomization will continue with the next sequential number.

17.3 Analysis

17.3.1 Inclusion of participants in the analysis

Participants will be included in the modified intention-to-treat analysis if they satisfied all the entry criteria and received the study intervention (screening by RDT or dose of SP) on at least one occasion, with no other exclusions. Women who give consent to enter the study but withdraw the same day and did not receive any study intervention (RDT or SP) will be classed as screening failures and not included in the analysis (e.g. in ongoing trials a few women gave consent, but then changed their mind during the same visit prior to, or during the study enrolment procedures).

Participants will be included in the per-protocol analysis if they satisfied all the entry criteria and either received the study intervention (IPTp-SP or ISTp-DP) on at least three separate occasions at least four weeks apart; or reached a study end-point before completion of the three-visit schedule but received the intervention at least once; or received an approved alternative treatment for symptomatic malaria according to protocol that replaced the need for the scheduled intervention. Participants who received the study medication but were reported not to have adhered to treatment (defined as taking at least 2/3 of the recommended dose over the recommended time period, on each occasion that treatment is required) or who used prohibited medication will be excluded from the per-protocol analysis. Women who were HIV negative at enrolment but HIV positive by the third trimester will be excluded from the per-protocol analysis.

17.3.2 Baseline comparability

Participants in each intervention group will be described separately with respect to baseline characteristics such as maternal age, gestational age, number of previous pregnancies, area of residence and anaemia on recruitment. The clinical importance of any imbalance will be noted and taken into account in further analyses.

17.3.3 Efficacy

The primary efficacy analysis will be the assessment of the superiority of ISTp-DP over IPTp-SP in women in their first and second pregnancies for the prevention of any of the following: SGA, pre-term birth or low birth weight. Similar analyses will be undertaken for the additional primary outcome of evidence of active or recent infection assessed at delivery by placental histopathology and RDT in women in their third to fifth pregnancies, and for the secondary efficacy outcomes. Impact will be determined by gravidity strata as the primary analysis. As a secondary analysis we will also estimate the overall estimated effect, extrapolated to all gravidity groups using a weighted analysis providing extra weight to the observations in multigravidae so that the final estimate represents the distribution of gravidity groups in the population attending antenatal clinics (based on antenatal clinic records).

Generalised linear models will be used where appropriate to assess the effect of the intervention according to different characteristics of the participants. Treatment effects will be expressed in terms of relative risk and risk difference for dichotomous variables and mean difference for continuous variables, and the corresponding 95% confidence intervals. Superiority will be defined as a positive treatment effect that is greater in the ISTp group than the IPTp group, where the 95% confidence interval does not include the point of no difference (RR value of 1).

Both modified intention-to-treat and per-protocol analyses will be undertaken for the efficacy analyses. The modified intention to treat analysis will be the primary analysis to assess superiority. In the absence of evidence for superiority, the prospectively defined non-inferiority margin will be 15% for adverse outcome or placental malaria in the intention to treat and per protocol populations (relative difference between endpoints of 15% (i.e. RR 0.85); e.g. 24.5% versus 28.8% for adverse birth outcome).[41]

17.3.4 Safety

Adverse events detected through passive surveillance and those requiring treatment will be grouped according to a pre-specified coding system and tabulated for each treatment arm on an intention-to-treat basis. Adverse events reported by women on DHA-PQ by active surveillance on day two after the first dose will be grouped according to a pre-specified coding system and tabulated for descriptive analysis only. Treatment emergent adverse events will be classified as those occurring within 24 hours after the first dose of a study drug. Adverse events that have missing onset dates will be considered as treatment emergent. The number and percentage of participants experiencing any adverse event, any serious adverse event, or any treatment emergent serious adverse event will be compared between intervention groups using Fishers Exact Test and Generalised linear models.

17.3.5 Immunology

Antibody levels in these samples will be analysed in relation to (i) the relative effect of the two different interventions on the development of general and pregnancy-specific immunity to *P. falciparum* malaria, and (ii) in multivariate analyses, in relation to pregnancy outcomes, to ask whether antibody level at enrolment predicts which women will respond best to IPTp or to ISTp. We will specifically ask whether immunity correlates with decreased risk of recurrent infection (recrudescence or reinfections), and with better pregnancy outcomes (birth weight, haemoglobin). In some models antibody response will be entered as the dependent variable, with treatment groups as exposure variable and other variables such as gravidity and age as covariates. In other models baseline antibody levels will be independent variables to determine if they are predictive of treatment outcome (e.g. birth outcome/ placental infection etc) and if pre-existing malarial immunity is a potential effect modifier of treatment effect.

17.3.7 Handling of missing data

Every effort will be made to minimize the amount of missing or incorrect data in the trial.

17.4 Criteria for early termination of the trial

We will use an adaptive sequential design using one interim analysis half-way for assessing whether to stop the trial early due to safety, efficacy or futility. The Lan-DeMets alpha spending function with O'Brien-Fleming type boundaries will be used to preserve the overall type I error rate for efficacy at the 0.05 level. Interim analyses will be conducted by the independent Data Monitoring Committee (DMC) supported by the trial statistician. The DMC will provide the trial steering committee only with a recommendation to continue, terminate or modify the trial.

17.5 CONSORT statement

Reporting of the statistical analysis will comply with CONSORT Guidelines.

17.6 Economics and implementability sub-study data analysis and management

Facility costs data will be collected. Economic datasets will be analysed using STATA, Microsoft Excel and TreeAge Pro.

Quantitative data from surveys and structured observations will be double entered into a database and validated. Qualitative data from FGDs and semi-structured interviews will be recorded electronically, transcribed and entered into N-Vivo or Atlas for extraction of themes.

18 Safety reporting

18.1 Definitions of adverse events

Definitions of different types of adverse events are presented in the Appendix 1.

18.2 Reporting procedures for all adverse events

An independent physician will be appointed by the sponsor (LSTM) to act as a study safety monitor. He or she will be based in Malawi.

All adverse events occurring during the study, observed by the investigator or reported by the participant, whether or not attributed to study medication, will be recorded using Adverse Event Forms. The following information will be recorded: description, date of onset and end date, severity, seriousness and action taken. Follow-up information will be provided as necessary.

The intensity of events will be assessed on the following scale: 1 = mild, 2 = moderate, 3 = severe, 4 = life-threatening, 5 = death

The relationship of adverse events to the study medication will be assessed by a medically qualified investigator and the independent safety monitor. If the safety monitor disagrees with the investigator' assessment, both opinions will be included in the report.

The safety monitor will evaluate the expectedness of each suspected adverse event according to the Summary of Product Characteristics for SP and the Investigators Brochure for DHA-PQ.

Any adverse reactions to the study drugs will be followed-up until resolution or stabilisation to ascertain the final outcome.

The investigator will judge whether or not an adverse event is of sufficient severity to require discontinuation of the study treatment. If this occurs, or if the participant herself wishes to withdraw due to what she considers to be an intolerable adverse reaction, she will be offered an end of study assessment and be given appropriate medical care until symptoms cease or the condition becomes stable. If she agrees she will be continued to be followed up according to schedule.

18.3 Reporting procedures for serious adverse events

The investigators will report all serious adverse events (SAEs) that occur in the course of the study to the safety monitor within 24 hours of the investigational site becoming aware of it. All SAEs will be reported, whether or not they are thought to be causally related to the study drugs. All SAEs will be reported using a standard SAE reporting form.

These immediate reports will be followed within the next 24 hours by detailed, written reports. The immediate and follow-up reports will identify participants by their study ID number.

In case of doubt about whether an event fulfils the criteria of serious, the case will be reported to the safety monitor who will assess whether the event should be reported as an SAE.

All SAEs will be reported by the safety monitor or PI to the sponsor (LSTM Research Office) within 48 hrs of discovery or notification of the event. The sponsor will be responsible for notifying the relevant Ethics Committees and the trial Data Monitoring Committee. Additional information received for a case (follow-up or corrections to the original case) will be detailed on a full report SAE form and emailed to the sponsor within 15 days including a report from the safety monitor. This information will be reviewed by the Data Monitoring Committee to consider any action that may be needed in response to reported adverse events.

18.4 Reporting of SAEs and AEs to the Malaria in Pregnancy Consortium

This study is conducted under the auspices of the Malaria in Pregnancy (MiP) Consortium which conducts treatment and prevention trials in 17 other malaria endemic countries (14 in Africa). All anonymized SAE reports and aggregated reports of adverse events will be reported to the secretariat of the safety working group of the Malaria in Pregnancy Consortium based at the Liverpool School of Tropical Medicine. The secretariat collates all SAE and AE reports from each of the trials for review by an Independent Safety Panel of the MiP Consortium that will meet at regular intervals (at least yearly) to review all SAEs reported for the different drugs used to treat and prevent malaria in pregnancy in the second and third trimesters of pregnancy as part of the ongoing trials. This will include prospective information from 860 other women treated with DHA-PQ (Zambia, Ghana, Burkina Faso, and Malawi (Chikwawa district hospital)), 860 women treated with amodiaquine artesunate, 1100 women treated with Coartem, and 1300 with mefloquine-artesunate, as part of other treatment trials, and information on approximately 7000 women treated with SP as part of IPTp.

The Independent Safety Panel consists of six members who are impartial and independent to the studies. It includes people with expertise in statistics, perinatal epidemiology, pharmacology, teratology/toxicology, pharmacovigilance, ethics and trials in developing countries. If so requested by the Data Monitoring Committee of individual trials, the Independent Safety Panel is able to provide advice to them.

19 Quality assurance procedures

The study will be conducted in accordance with the current approved protocol, ICH GCP, relevant regulations and standard operating procedures. Regular monitoring will be performed according to ICH GCP.

The Research Support Centre of the College of Medicine will provide the clinical monitoring on behalf of the sponsor. Prior to participant enrolment, the monitor will visit the study sites to assess the adequacy of the facilities, review the protocol and data collection procedures and discus the responsibilities of the investigator and other study site personnel. The monitor will also make a visit shortly after the start of enrolment, and at periodic intervals during the trial, to ensure that it is being conducted according to the published protocol and any amendments.

The trial Data Monitoring Committee will meet at least annually, with additional meetings being held if there are any specific issues arising.

Direct access to the trial source data will be granted to authorised representatives from the sponsor, host institution and the regulatory authorities to permit trial-related monitoring, audits and inspections. This will be explained to the participants on the consent form or participant information sheet.

20 Procedures for handling and accounting for study drugs

20.1 Labelling of study drugs

Drug labelling will be in the local language and in accordance with local regulations. Standard GMP required labelling will be used including:

- Name, address and mobile telephone number of the Principal Investigator
- Study drugs, dosage form, route of administration, and quantity of dosage form
- Batch number of code to identify the contents
- Directions for use
- Expiry date
- Storage conditions
- ‘for clinical study use only’
- ‘keep out of reach of children’

20.2 Storage of study drugs and RDTs

All study drugs and RDTs will be stored in secure areas with access limited to the investigator and authorised study personnel, and under appropriate storage conditions. A description of the appropriate product-specific storage conditions will be specified on the product pack labels. The duration period of the trial will not exceed the shelf-life of the study drugs or RDTs.

20.3 Accountability for the study drugs

The Principal Investigator will be responsible for establishing a system for the correct handling of study drugs and RDTs to ensure that:

- Deliveries of study drug from the sponsor are correctly received by a responsible person (e.g. pharmacist assistant)
- Accurate records are maintained for receipt of the study drug, for the dispensing of the study drug to subjects, and for returned drugs
- Certificates of delivery and return are signed by the investigator or authorised study personnel and copies retained in the investigator file
- Study drugs are handled and stored safely and properly an in agreement with the given storage instructions
- Study drugs are prescribed only by the principal investigator, co-investigators or study site personnel authorised to do so by the principal investigator
- Study drugs are dispensed only to study participants in accordance with the protocol
- Delivery records are reconciled with records of usage and returned stock, and any discrepancies accounted for in writing
- Once accounted for, any unused study drugs are destroyed or donated as agreed by the sponsor

21 Record keeping and archiving

During the study, an Investigator Study File will be used to store documentation pertaining to the study and it will be kept in a secure location with access only to authorised individuals. It is the Principal Investigator's responsibility to continuously update the file. It will be available to the Monitor during monitoring visits.

Following study closure, the investigator study file, CRFs, medical records and other study specific source documents (where appropriate) will be retained at the study site according to regulatory obligations and thereafter destroyed only with the agreement of the sponsor.

22 Ethics

22.1 ICH Guidelines for Good Clinical Practice

The Principal Investigator will ensure that this study is conducted in full conformity with relevant regulations and with the International Conference on Harmonization (ICH) guidelines for Good Clinical Practice (GCP) (CPMP/ICH/135/95) July 1996.

22.2 Approvals

The protocol, informed consent form and participant information sheets will be submitted to the research ethics committees at the College of Medicine (Blantyre, Malawi) and Liverpool School of Tropical Medicine for approval:

Before initiating the trial, written and dated approval will be obtained from both relevant ethics committees. Any protocol amendments will be submitted to the ethics committees before implementation. Progress reports, SUSAR reports and safety reports will be submitted in accordance with local requirements.

22.3 Informed consent

Informed consent will be obtained before women are enrolled in the study. See section 11 and Appendix 2 for further details.

22.4 Participant confidentiality

The study staff will ensure that the participants' anonymity is maintained. The participants will be identified only by their study identification number and initials on the CRF and any electronic database. All documents will be stored securely and will only be accessible by study staff and other authorised personnel. All participant data will be anonymized as soon as it is practical to do so.

22.5 Other ethical considerations

22.5.1 Safety of the study drugs in pregnancy

Both SP and DHA-PQ are currently thought to be safe for the mother and foetus during the second and third trimesters of pregnancy. However, adverse events, particularly those associated with the study medication, will be recorded and monitored throughout the trial. An interim analysis of safety outcomes will be undertaken approximately half way through the trial and the trial will be stopped if there are any apparent safety concerns arising from this analysis. The trial may be stopped or temporarily suspended by the sponsor at any stage due to any arising safety concerns.

22.5.2 Blood sampling

All examinations undertaken as part of this study will be non-invasive, with the exception of blood sampling. Wherever possible, blood sampling will be by finger-prick only. Venous blood samples of 5 ml will be taken before enrolment, for eligibility screening/study-specific baseline testing. The sample will be taken at the screening stage because relatively few women are expected to be excluded from the trial based on blood test results (if HIV-positive or severely anaemic); thus while a few women will have a venous sample (rather than a finger-prick sample) taken unnecessarily, a far greater proportion will be saved from having a finger prick sample followed at the same visit by a venous sample.

Another 5 ml venous sample will be taken at the final antenatal visit and at birth, where routine care and study-specific samples will be combined in a single blood draw.

Blood sampling may be inconvenient to the participants, and may cause minor discomfort and bruising. In some aspects of the trial, blood sampling has the potential to directly benefit the participants or their babies, as any malaria infection or anaemia detected as a result of the sampling will be treated. In other aspects of the trial, such as drug level monitoring and the taking of drug for blood smears during pregnancy, there will be no direct benefit to the individual woman, but the potential knowledge gained will eventually be used to benefit all pregnant women and their babies in areas with similar characteristics to southern Malawi.

The volume of blood collected from each participant will be small, a maximum of 20 ml per woman over the course of the study. Only well trained nursing and laboratory staff will be employed on the trial. Only new disposable needles and lancets will used for blood taking procedures, and these will be safely discarded immediately after their use.

22.5.3 Shipping and Storage of blood samples

Two novel assays developed by Professor Rogerson and colleagues from the University of Melbourne will be used in immunology studies; antibodies that block adhesion to placental vesicles; and antibodies that opsonise malaria infected cells for phagocytic clearance. The former assay is subject of a grant application in Australia, which includes funding for a Malawian scientist to travel to Australia for three months to learn the assay, and to then take it back to Blantyre. The other assay requires large amounts of parasite and cell culture, at levels which are beyond the capacity of the laboratories in Malawi, due to difficulties in sourcing fresh uninfected blood and malaria-naive serum for culture, so it is only feasible to perform these in Melbourne at present. The Malawian scientist will learn this assay, but will focus primarily on the first.

22.5.4 Benefits to study participants

By taking part in this trial, participants will receive either IPTp-SP on a three-dose schedule, which is usual care in Malawi; or they will receive ISTp with DHA-PQ, which we anticipate to be superior to IPTp. Participants who do not attend scheduled appointment will receive reminders and active follow-up. Participants experiencing illness between visits will be seen and treated free of charge as part of the study; however this is available to all pregnant women in Malawi.

22.5.5 Inclusion of young people under the age of 18

This study will include young women aged 16 and 17. Girls younger than 16 will not be included because they will be referred for care at tertiary facilities according to local guidelines. In Malawi, young pregnant women aged 15 or older are considered emancipated, and are legally able to consent on their own behalf to be included in a clinical trial. It is important to include young women in the trial, as adolescents are known to be particularly susceptible to malaria in pregnancy, and are therefore one of the groups that may potentially benefit the most from any improvements to practice in preventing adverse outcomes related to malaria in pregnancy.

22.5.6 Reimbursement of costs

The study will provide payment for all study drugs, study procedures, study-related visits and reasonable medical expenses that are incurred as a result of the study. This includes expenses for transport and a meal allowance for any study visits which are conducted over lunch times.

23 Financing and insurance

Funding for this study will be provided by the European and Developing Countries Clinical Trials Partnership (EDCTP). Liverpool School of Tropical Medicine will act as sponsor and has insurance that covers this study.

24 Publication policy

The results of the study will be submitted to and discussed with local and national medical authorities in Malawi, and will then be presented at national and international conferences and submitted for publication in peer-reviewed journals, in accordance with the sponsor's and the University of Malawi College of Medicine's publication policy.

25 References

1. Desai M, ter Kuile FO, Nosten F, McGready R, Asamoa K, Brabin B, Newman RD, 2007. Epidemiology and burden of malaria in pregnancy. Lancet Infect Dis 7: 93-104.
2. Le Hesran JY, Cot M, Personne P, Fievet N, Dubois B, Beyeme M, Boudin C, Deloron P, 1997. Maternal placental infection with Plasmodium falciparum and malaria morbidity during the first 2 years of life. Am J Epidemiol 146: 826-31.
3. Malhotra I, Dent A, Mungai P, Wamachi A, Ouma JH, Narum DL, Muchiri E, Tisch DJ, King CL, 2009. Can prenatal malaria exposure produce an immune tolerant phenotype? A prospective birth cohort study in Kenya. PLoS Med 6: e1000116.
4. Mutabingwa TK, Bolla MC, Li JL, Domingo GJ, Li X, Fried M, Duffy PE, 2005. Maternal malaria and gravidity interact to modify infant susceptibility to malaria. PLoS Med 2: e407.
5. Schwarz NG, Adegnika AA, Breitling LP, Gabor J, Agnandji ST, Newman RD, Lell B, Issifou S, Yazdanbakhsh M, Luty AJ, Kremsner PG, Grobusch MP, 2008. Placental malaria increases malaria risk in the first 30 months of life. Clin Infect Dis 47: 1017-25.
6. Cornet M, Le Hesran JY, Fievet N, Cot M, Personne P, Gounoue R, Beyeme M, Deloron P, 1998. Prevalence of and risk factors for anemia in young children in southern Cameroon. Am J Trop Med Hyg 58: 606-11.
7. van Eijk AM, Ayisi JG, Ter Kuile FO, Misore AO, Otieno JA, Kolczak MS, Kager PA, Steketee RW, Nahlen BL, 2002. Malaria and human immunodeficiency virus infection as risk factors for anemia in infants in Kisumu, western Kenya. Am J Trop Med Hyg 67: 44-53.
8. Reed SC, Wirima JJ, Steketee RW, 1994. Risk factors for anemia in young children in rural Malawi. Am J Trop Med Hyg 51: 170-4.
9. Owens Sea, 2009. Placental malaria and gravidity interact to modify infant susceptibility to malaria. Arch Dis Child 91: 507-8.
10. WHO, 2004. A strategic framework for malaria prevention and control during pregnancy in the African Region. Brazzaville: World Health Organization Regional Office for Africa.
11. ter Kuile FO, van Eijk AM, Filler SJ, 2007. Effect of sulfadoxine-pyrimethamine resistance on the efficacy of intermittent preventive therapy for malaria control during pregnancy: a systematic review. JAMA 297: 2603-16.
12. WHO, 2006. Guidelines on co-trimoxazole prophylaxis for HIV-related infections among children, adolescents and adults: recommendations for a public health approach. Geneva.
13. WHO, 2010. Guidelines for the treatment of malaria: second edition. Geneva: World Health Organization.
14. Harrington WE, Mutabingwa TK, Muehlenbachs A, Sorensen B, Bolla MC, Fried M, Duffy PE, 2009. Competitive facilitation of drug-resistant Plasmodium falciparum malaria parasites in pregnant women who receive preventive treatment. Proc Natl Acad Sci U S A 106: 9027-32.
15. Feng G, Simpson JA, Chaluluka E, Molyneux ME, Rogerson SJ, Submitted. Decreasing burden of malaria in pregnancy in Malawian women and its relationship to use of intermittent preventive therapy or bed nets
16. Nkhoma S, Molyneux M, Ward S, 2007. Molecular surveillance for drug-resistant Plasmodiumfalciparum malaria in Malawi. Acta Trop 102: 138-42.
17. Njagi JK, Magnussen P, Estambale B, Ouma J, Mugo B, 2003. Prevention of anaemia in pregnancy using insecticide-treated bednets and sulfadoxine-pyrimethamine in a highly malarious area of Kenya: a randomized controlled trial. Trans R Soc Trop Med Hyg 97: 277-82.
18. Greenwood B, Alonso P, ter Kuile FO, Hill J, Steketee RW, 2007. Malaria in pregnancy: priorities for research. Lancet Infect Dis 7: 169-74.

19. Worrall E, Morel C, Yeung S, Borghi J, Webster J, Hill J, Wiseman V, Mills A, 2007. The economics of malaria in pregnancy--a review of the evidence and research priorities. Lancet Infect Dis 7: 156-68.
20. Helitzer-Allen DL, McFarland DA, Wirima JJ, Macheso AP, 1993. Malaria chemoprophylaxis compliance in pregnant women: a cost-effectiveness analysis of alternative interventions. Soc Sci Med 36: 403-7.
21. Schultz LJ, Steketee RW, Chitsulo L, Macheso A, Kazembe P, Wirima JJ, 1996. Evaluation of maternal practices, efficacy, and cost-effectiveness of alternative antimalarial regimens for use in pregnancy: chloroquine and sulfadoxine-pyrimethamine. Am J Trop Med Hyg 55: 87-94.
22. Schultz LJ, Steketee RW, Chitsulo L, Wirima JJ, 1995. Antimalarials during pregnancy: a cost-effectiveness analysis. Bull World Health Organ 73: 207-14.
23. Briand V, Bottero J, Noel H, Masse V, Cordel H, Guerra J, Kossou H, Fayomi B, Ayemonna P, Fievet N, Massougbodji A, Cot M, 2009. Intermittent treatment for the prevention of malaria during pregnancy in Benin: a randomized, open-label equivalence trial comparing sulfadoxine-pyrimethamine with mefloquine. J Infect Dis 200: 991-1001.
24. Clerk CA, Bruce J, Affipunguh PK, Mensah N, Hodgson A, Greenwood B, Chandramohan D, 2008. A randomized, controlled trial of intermittent preventive treatment with sulfadoxine-pyrimethamine, amodiaquine, or the combination in pregnant women in Ghana. J Infect Dis 198: 1202-11.
25. NCT01103063, (accessed 12/05/2010). Evaluate azithromycin plus chloroquine and sulphadoxine plus pyrimethamine combinations for intermittent preventive treatment of falciparum malaria infection in pregnant women in Africa. http://clinicaltrials.gov/ct2/show/NCT01103063.
26. Villar J BP, 2003. WHO antenatal care randomized trial; manual for the implementation of the new model Geneva: WHO.
27. Tagbor H, Bruce J, Agbo M, Greenwood B, Chandramohan D, mansucript in preparation. Effectiveness of malaria rapid diagnostic testing and treatment compared with intermittent preventive treatment in the control of malaria in pregnancy: a randomised, controlled, non-inferiority trial.
28. Smith LA, Jones C, Adjei RO, Antwi GD, Afrah NA, Greenwood B, Chandramohan D, Tagbor H, Webster J, 2010. Intermittent screening and treatment versus intermittent preventive treatment of malaria in pregnancy: user acceptability. Malar J 9: 18.
29. NCT01084213, 2010. Intermittent preventive treatment versus scheduled screening and treatment of malaria in pregnancy. http://clinicaltrials.gov/ct2/show/NCT01084213.
30. Mockenhaupt FP, Ulmen U, von Gaertner C, Bedu-Addo G, Bienzle U, 2002. Diagnosis of placental malaria. J Clin Microbiol 40: 306-8.
31. Leke RF, Djokam RR, Mbu R, Leke RJ, Fogako J, Megnekou R, Metenou S, Sama G, Zhou Y, Cadigan T, Parra M, Taylor DW, 1999. Detection of the Plasmodium falciparum antigen histidine-rich protein 2 in blood of pregnant women: implications for diagnosing placental malaria. J Clin Microbiol 37: 2992-6.
32. Smithuis F, Kyaw MK, Phe O, Win T, Aung PP, Oo AP, Naing AL, Nyo MY, Myint NZ, Imwong M, Ashley E, Lee SJ, White NJ, 2010. Effectiveness of five artemisinin combination regimens with or without primaquine in uncomplicated falciparum malaria: an open-label randomised trial. Lancet Infect Dis 10: 673-81.
33. Sinclair D, Zani B, Donegan S, Olliaro P, Garner P, 2009. Artemisinin-based combination therapy for treating uncomplicated malaria. Cochrane Database Syst Rev: CD007483.
34. Myint HY, Ashley EA, Day NP, Nosten F, White NJ, 2007. Efficacy and safety of dihydroartemisinin-piperaquine. Trans R Soc Trop Med Hyg 101: 858-66.
35. Rijken MJ, McGready R, Boel ME, Barends M, Proux S, Pimanpanarak M, Singhasivanon P, Nosten F, 2008. Dihydroartemisinin-piperaquine rescue treatment of multidrug-resistant

Plasmodium falciparum malaria in pregnancy: a preliminary report. Am J Trop Med Hyg 78: 543-5.
36. Parise ME, Ayisi JG, Nahlen BL, Schultz LJ, Roberts JM, Misore A, Muga R, Oloo AJ, Steketee RW, 1998. Efficacy of sulfadoxine-pyrimethamine for prevention of placental malaria in an area of Kenya with a high prevalence of malaria and human immunodeficiency virus infection. Am J Trop Med Hyg 59: 813-22.
37. Filler SJ, Kazembe P, Thigpen M, Macheso A, Parise ME, Newman RD, Steketee RW, Hamel M, 2006. Randomized Trial of 2-Dose versus Monthly Sulfadoxine-Pyrimethamine Intermittent Preventive Treatment for Malaria in HIV-Positive and HIV-Negative Pregnant Women in Malawi. J Infect Dis 194: 286-93.
38. WHO, 2010. Delivering HIV test results and messages for re-testing and counselling in adults. Geneva: World Health Organization.
39. Gamble C, Ekwaru JP, ter Kuile FO, 2006. Insecticide-treated nets for preventing malaria in pregnancy. Cochrane Database Syst Rev: CD003755.
40. Landis SH, Lokomba V, Ananth CV, Atibu J, Ryder RW, Hartmann KE, Thorp JM, Tshefu A, Meshnick SR, 2009. Impact of maternal malaria and under-nutrition on intrauterine growth restriction: a prospective ultrasound study in Democratic Republic of Congo. Epidemiol Infect 137: 294-304.
41. CPMP, 2001. Points to consider on switching between superiority and non-inferiority (Committee for Proprietory Medicinal Products). British Journal of Clinical Psychology 52: 223-8.

Appendix 1: Definitions of adverse events

Adverse Event

Any untoward medical occurrence in a study participant administered a study intervention.

Adverse Reaction

An adverse reaction is an adverse event judged by either the reporting medically qualified professional or the sponsor as having a reasonable suspected causal relationship to the study medication.

Severe Adverse Event

The term "severe" is used to describe the intensity (severity) of a specific event (as in mild, moderate, or severe). The event itself, however, may be of relatively minor medical significance (such as severe headache).

Serious Adverse Event or Serious Adverse Reaction (SAE/SAR)

Any untoward medical occurrence that:

- Results in death
- Is life-threatening
- Requires hospitalisation or prolongation of existing hospitalisation
- Results in persistent or significant disability/incapacity
- Is a significant congenital anomaly

Suspected Expected Serious Adverse Reactions (SSAR)

An adverse reaction that is classed as serious and which is consistent with the information about the study drug set out in the Summary of Product Characteristics (SP) or Investigator's Brochure (DHA-PQ).

Suspected Unexpected Serious Adverse Reactions (SUSAR)

A serious adverse reaction, the nature or severity of which is not consistent with the applicable product information described above.

Appendix 2: Participant information and consent forms

PATIENT INFORMATION SHEET ISTp STUDY

The University Of Malawi College Of Medicine and the Liverpool School of Tropical Medicine are jointly doing a research study. We invite you to take part in this study. Before you decide whether to take part, it is important for you to understand why the study is being done and what it will involve. Please take the time to read the following information carefully. Ask us if there is anything that is not clear and if you would like more information.

What is the purpose of the study?

Malaria is a common cause of anaemia (lack of blood) in pregnancy and can cause the baby to be born small and weak. Sometimes you can have malaria without feeling sick. To help prevent malaria in pregnancy, you would usually be given three tablets of sulphadoxine-pyrimethamine (SP or Fansidar) three or four times during pregnancy when you visit the clinic after you first feel the baby move. This strategy is called IPTp-SP, which stands for **I**ntermittent **P**reventive **T**reatment in **p**regnancy with **S**ulphadoxine-**P**yrimethamine.

Malaria parasites are now becoming resistant to SP, meaning that the drug may not work as well as it used to. We want to evaluate a new strategy for control of malaria in pregnancy called ISTp. ISTp stands for **I**ntermittent **S**creening and **T**reatment in **p**regnancy, and consists of testing women for malaria three or four times during pregnancy, and, if they have malaria, treating them with a drug called dihydroartemisinin-piperaquine (DHA-PQ), which is used to cure malaria. We want to compare the new strategy (ISTp) with IPTp-SP given three or four times.

Why have I been chosen?

We need women who are between 16 and 28 weeks pregnant, and who would normally be scheduled to receive IPTp-SP, to take part in this study. If you are less than 16 weeks pregnant, we would like you to join the study after you are 16 weeks pregnant, when you next come to the clinic.

What will happen if I want to take part?

If you think you would like to be in this study, we will first invite you to be screened, to see if you are suitable. If you agree we will first take a small amount of blood from your arm (about a teaspoon) and test it for anaemia and HIV, unless you have already had an HIV test in this pregnancy. Either then or later (perhaps after you join the study) we will do an ultrasound scan of your baby. An ultra-sound scan is a test which shows a moving picture of the baby inside you. You will be asked to lie down, a cool gel will be spread over your belly and a small machine will be moved gently over it. We will use the ultrasound picture to know when your baby is due to be born. Ultra-sound scans are completely painless and harmless to you and the baby. They are very common in Europe and America.

If the blood tests show that you are suitable for this study, and you agree to take part, we will ask you some questions about your health. We will do a physical examination, measure your

weight, and copy some information from your health passport and from the lab books. We will use the rest of blood sample that we have already taken to find out whether you have malaria, and the nurse will also do all the other routine tests that pregnant women usually have, unless you have already had these tests. If you consent, we will also send some of this blood sample abroad for special tests of your body' resistance against the malaria parasite (immunity) and the resistance of the parasite. We will try not to prick your arm again at this appointment. The examination and tests will take about ten minutes altogether.

You will then open an envelope with a piece of paper inside which describes which treatment you will get. The treatment you get is decided by chance, like flipping a coin. It will be either:

- ***Group 1 - ISTp***

  If you are in this group, we will test the blood sample for malaria using a rapid diagnostic test. The test results will take about 15 minutes. If the results show that you have malaria, we will give you DHA-PQ tablets to take in the clinic and some more tablets to take at home on the following day and the day after that. If you are given DHA-PQ, a fieldworker may visit you at home on the second day after your appointment to ask whether you have taken the tablets and whether you had any problems with them. You will only receive DHA-PQ if you have malaria.

  Or

- ***Group 2 -* IPTp-SP** (current standard of care)

  If you are in this group you will be given three tablets of SP to take at the clinic. You will not be tested for malaria.

You will then be asked to return to the study clinic around two or three more times, when you will be given the same treatment that you received on your first visit. If you are tested for malaria at one of these visits we will take only a tiny amount of blood from a finger prick. At one of these visits, we will ask you for an additional blood sample, taken from a finger, which we will test for malaria at the end of the study. We will also use this blood sample to test you for anaemia. If we test you and find that you have anaemia, we will treat your anaemia at this visit.

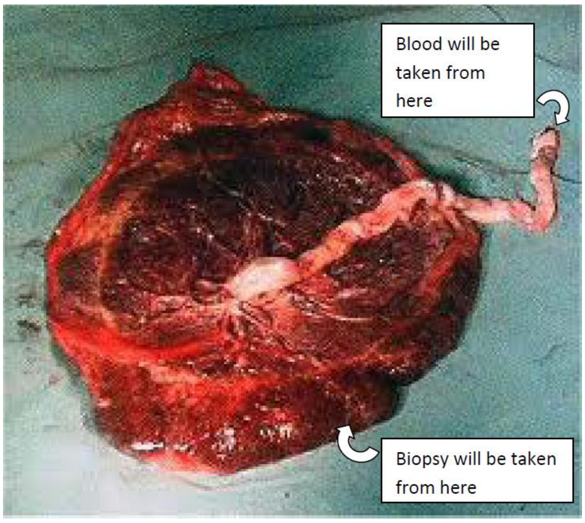

When you come for your clinic visits we will give you enough money for transport to and from the clinic and, if you miss lunch, for a light meal. You will also receive all other health care that pregnant women attending the clinic would usually receive.

We would like you to give birth to your baby in the maternity ward of the clinic so that we can check how you and the baby are doing. After your baby is born we will check the placenta for malaria (see picture).

We will take a blood sample from your arm and test it for malaria, your level of immunity to malaria, and anaemia. We will measure your

baby's weight and give the baby a health check. If you want to give birth to your baby at home, we would like you to inform us as soon as possible when the baby is born, so we can visit you at home and collect information for the study.

We would like to see you again with your baby when baby is seven days old and again when they are about six to eight weeks old, to check that you and your baby are still doing well. You will not need to make an extra journey to the clinic, as we will see you when you bring your baby for their routine vaccinations.

If you are in the study, we would prefer that you do not take any medicine that is not provided by the clinic. If you feel unwell during the study you can come to the clinic. You will be seen by a qualified member of staff free of charge and will be given any treatment that you need.

The information collected will be used by staff involved in the study and by scientists in Malawi and abroad. It will be kept confidential. Names and addresses will not appear on any of the study reports.

What if I don't want to be in the study?

If you decide not to be in the study, you will receive all the usual health care that pregnant women attending the clinic usually receive. This will include getting three tablets of SP when you visit the clinic at least two times during pregnancy after you first feel the baby move, and again several times later in your pregnancy. You can change your mind and withdraw from the study at any time, and you do not have to give a reason if you do not want to.

What are the possible disadvantages and risks of taking part?

DHA-PQ is a very effective treatment for malaria. It has already been tested in pregnant women after 13 weeks of pregnancy and it does not seem to cause any harm to women or their babies. However, it is still quite a new drug and we cannot be completely sure that it will not have any side-effects. If you appear to suffer bad side-effects during the study, we will stop your treatment with DHA-PQ. All babies will be given a thorough health check and any problems will be recorded. The study will be stopped if we suspect that DHA-PQ is causing health problems in the babies

SP has been widely used during pregnancy, is very safe, and is given to all pregnant women who attend clinics in Malawi. You will also be given SP if you do not take part in the study.

If do you become ill after taking DHA-PQ or SP, and we think it might be because of the drug, we may stop your treatment (not give you the same drug again) but if this happens we would still like you to continue in the study.

When we take blood, you may get a small bruise or mild pain on the finger or arm where the blood is taken. There is also a very small chance of infection; the chance is very small because we always use clean materials.

The study will require you to make more trips to the clinic than usual, which may be inconvenient. However, we will give you money to pay for the travel costs.

Thank you very much for your time. Would you like to be screened to join the study?

Study consent form (English)

CONSENT FOR SCREENING

Screening will involve taking a small sample of blood from your arm for testing, and possibly also an ultrasound scan.

If you agree to be screened, this does not mean that you have to agree to be in the study, you can change your mind at any time.

The above study has been explained to me and I agree to be screened to see if I am suitable to be in the study.

Participant's statement: sign or thumbprint on the appropriate line. If the participant does not wish to consent, form should not be signed.

The above has been explained to me	I agree to be part of this study	
Participant's name:	Participant 's signature:	Date: (day/month/year)

Witness' statement (optional, except for illiterate participants):

The above consent was explained and the woman agreed to be screened:

Witness' name	Witness' signature	Date: (day/month/year)

Investigator's name:	Investigator 's signature:	Date: (day/month/year)

CONSENT FOR INCLUSION IN THE STUDY

The above study has been explained to me and I agree to join.

- I have been told about the risks of and benefits of being in the study.
- I have been told that it is up to me if I want to join the study and that I can leave the study any time I want without any consequences for me and my baby.
- I agree to have a home visit by the study staff if I am not able to come to the study clinic.

Participant's statement: sign or thumbprint on the appropriate line. If the participant does not wish to consent, form should not be signed.

The above has been explained to me	I agree to be part of this study	
Participant's name:	Participant 's signature:	Date: (day/month/year)

Witness' statement (optional, except for illiterate participants):
The above consent was explained and the woman agreed to join the study:

Witness' name	Witness' signature	Date: (day/month/year)

Investigator's name:	Investigator 's signature:	Date: (day/month/year)

<u>Contacts</u>

If you later have questions or concerns about your participation in this study, you may speak with one of our staff. If you have any questions about the study, or you feel your child has been harmed, please contact Dr. Linda Kalilani-Phiri at the College of Medicine, Private Bag 360, Blantyre 3 or at phone number 01919776**.** If you have any questions about your rights

as a study participant, or if you want to talk about the study with someone who is not part of this research project, please contact Prof Mfutso-Bengo at the College of Medicine, Private Bag 360, Blantyre 3 or phone number 01877291**.**

Study consent form continued (English)

CONSENT FOR TRANSPORT AND STORAGE OF BLOOD SAMPLES

We would like to send part of the blood sample abroad for special tests of your body' resistance against the malaria parasite (immunity) and the resistance of the parasite. We do not have the equipment to be able to do these tests in Malawi at present.

We would also like to store a part of the blood sample collected in this research in case there are additional tests that we wish to perform after the study is over. The sample will be sent to the USA and stored there. We will not store your samples without your permission.

If you allow us to transport and store your samples, you may change your mind and withdraw up to one month after you complete the study. If you would like your samples to be removed from storage, you may contact Dr. ______________________ at phone number _______________.

One month after you complete the study, we will remove your name from the blood samples sent for storage. After your name is removed, we will not be able to take your samples out of storage if you change your mind. Also, we will not be able to report any future test results to you.

You can be in the study even if you do not want blood samples stored.

Please check one of the boxes below to indicate whether you do or do not allow us to store your blood.

- ☐ NO, I wish my blood samples to be destroyed immediately.
- ☐ YES, I give permission for my blood samples to be stored anonymously

Participant's statement: sign or thumbprint on the appropriate line. If the participant does not wish to consent, form should not be signed.

The above has been explained to me	I agree to be part of this study	
Participant's name:	Participant 's signature:	Date: (day/month/year)

Witness' statement (optional, except for illiterate participants):
The above consent was explained and the woman agreed to join the study:

Witness' name	Witness' signature	Date: (day/month/year)

Investigator's name:	Investigator 's signature:	Date: (day/month/year)

PATIENT INFORMATION SHEET ISTp STUDY (Chichewa)

A ku University of Malawi College of Medicine ndi aku Liverpool School of Tropical Medicine akugwirira ntchito limodzi mukafukufukuyi. Tikukupemphani kuti mutenge nawo mbali mukafukufukuyi. Musanasankhe kutenga nawo mbali mukafukufukuyi, ndikofunikira kuti mumvetsetse chifukwa chomwe kafukufukuyi akupangidwira ndi zomwe zizichitika mukafukufukuyi. Chonde mutenge nthawi kuwerenga bwinobwino mfundo zomwe zalembedwazi. Mutifunse ngati pali chilichonse chomwe simukumvetsa ndiponso ngati mukufuna tikulongosolereni chilichonse.

Cholinga cha kafukufukuyi ndi chani?

Malungo amapangitsa amayi oyembekezera kutha magazi ndiponso kuti mwana abadwe ochepa thupi ndiponso ofooka. Nthawi zina mukhoza kukhala ndi malungo koma osamva kudwala mthupi. Kuti mupewe malungo mukakhala oyembekezera, mumapatsidwa ma tabuleti atatu a mankhwala a sulphadoxine-pyrimethamine (SP kapena Fansidar) mukabwera kuchipatala; mumalandira mankhwalawa koyamba mukamva mwana kusuntha ndiponso kenanko nthawi ina pakapita nthawi. Izi timazitchula kuti **I**ntermittent **P**reventive **T**reatment in **p**regnancy with **S**ulphadoxine-**P**yrimethamine.

Kachilombo kamene kamapangitsa matenda a malungo kapeza njira yogonjetsa mankhwala a SP, motero mankhwalawa akhoza osagwira ntchito mmene ankagwirira kale. Tikufuna tiyeze njira ina yotetezera malungo mu azimayi oyembekezera yotchedwa ISTp. ISTp imatanthauza **I**ntermittent **S**creening and **T**reatment in **p**regnancy, ndipo tikamagwiritsira njirayi timayeza amayi malungo katuta panthawi yomwe akuyembekezera, ndipo akapezeka kuti ali ndi malungo timawapatsa mankhwala otchedwa dihydroartemisinin-piperaquine (DHA-PQ), kuti achilitse malungo. Tikufuna tisiyanitse mmene njira yatsopano ya (ISTp) kuyerekeza ndi kupereka fansidar katatu kwa a mayi ammimba imagwirira ntchito kuti tione njira yomwe ili yoposa inzake. Kafukufuku waonetsa kuti SP amagwira bwino ntchito akaperekedwa katatu kwa a zimayi oyembekezera osati kawiri.

Chifukwa chain ndasankhidwa?

Tikufuna azimayi amene ali oyembekezera pakati ma milungu khumi isanu ndi umodzi ndi milungu makumi awiri isanu ndi itatu, amene amayenekera kulandira IPTp-SP, kuti atenge nawo mbali mukafukufuku. Mukakhala kuti mimba yanu ndiyochepera milungu khumi isanu ndi umodzi, tikupemphani kuti muzalowe mukafukufuku mimba yanu ikaposa milungu khumi isanu ndi umodzi, mukadzabweranso kuchipatala.

Ndondomeko yake ikhala yotani ndikatenga nawo mbali mukafukufukuyi?

Ngati mukuona ngati mufuna kutenga nawo mbali mukafukufuku, tikupemphani kuti tiunike kuti tione ngati muli oyenera kutenga nawo mbali mukafukufukuyi. Ngati mulole poyamba titenga magazi pang'ono kuchokera pamkkono panu (okwanira supuni imodzi) kuti tiyeze ngati muli ndi magazi ochepa, ndi kuyeza matenda a HIV. Koma sitizakuyezani HIV ngati munayezedwa kale matenda a HIV muli ndi mimbayi. Tizakuyezani kugwiritsira ntchito machini a ultrasound kuti tione mwana wanu. Ultra-sound scan imaonetsa mmene mwana wanu akukhalira mmimba. Tizakupemphani kuti mugone pa bedi lamuchipinda choyeza

ndipo tizayika mavuta pang'ono pamimba panu ndipo makina tizawayendetsa pamimbapo pang'ono pang'ono. Tizagwiritsira ntchito zithunzi cha ultrasound kuti tiyeze kuti mwana wanu abadwa liti. Kukuyezani kugwiritsira Ultra-sound ndikosapweteka ndipo sikungapweteke mwana wanu munjira ili yonse. Ultra-sound imagwiritsidwa ntchito nthawi zambiri ntchito ku Europe ndi ku America.

Ngati zoyeza zamagazi ndi ultrasound scan iwonetse kuti muli oyenera kutenga nawo mbali mukafukufuku, ndipo ngati mulole kutenga mbali mukafukufukuyi, tikufunsani mafunso angapo okhudza zaumoyo wanu. Tizakuyezani mthupi, ndikuyeza kuti mukulemera bwanji, ndiponso tizatenga mfundo zina kuchokera mu bukhu lanu lakuchipatala ndi kalata yakusikelo koma mubukhu lomwe amayika zotsatira zakuyeza. Tidzatenganso magazi otsalira amene tinatenga poyamba aja kuti tiyeze ngati muli ndi malungo ndiponso kuti muli ndichitetezo chokwanira bwanji mthupi wanu choteteza kumalungo. A namwino azayezanso zinthu zina zomwe zimayezedwa nthawi zonse kusikelo, azayeza kupatula zomwe mwayezedwa kale. Tiyeseta kuti tizakubayeni pamkono kawiri ulendo uno. Kukuyezani ndikutenga maganiza okayeza kuzatenga mphindi khumi.

Tikapanga izi muzatsegula envelopu ndipo mkati mwake muzalembedwa chithandizo chomwe mulandire. Chithandizo chomwe mulandire chizasankhidwa mwa mwayi ngati mmene umapangira kusankha munthu poponya khobiri mwamba kenako kuona kuti ndi mbali iti wasankha munthu. Mukhala mugulu limodzi mwa awiri tatchula aja.

Gulu loyamba - ISTp

Ngati mukhale mugulu ili, tizayeza magazi anu kuti tione ngati muli ndi malungo kugwiritsira ntchito rapid diagnostic test. Muzamva zotsatria mu mphindi makumi ndi asanu. Ngati zotsatira zionetse kuti muli ndi malungo, tidzakupatsani mankhwala a DHA-PQ kuti mumwe. Mankhwala ena mumwera kuchipatala konkuno, ena mukamwera kunyumba tsiku lotsatira tsiku lomwe mwayamba kumwa mankhwalawo. Ngati mupatsidwe DHA-PQ, ogwira ntchito zaumoyo kumamidzi akhoza kuzakuyenderani kunyumba tsiku lachiwiri kuchokera mmene takuonani kuchipatala kuti adzawone ngati mwamwa mankhwala tikupatseniwa ndiponso adzafuna kudziwa ngati muli ndi mavuto ena ali wonse ndi mankhwalawa. Muzizalandira mankhwala a DHA-PQ pokhapokha ngati muzapezeke ndi malungo nthawi ya kafukufuku.

Kapena

Gulu lachiwiri - **IPTp-SP** (lopanda kuyeza malungo)

Tizakupemphani kuti muzabwere kuchipatala kawiri kapena katatu, ndipo muzalandira mankhwala amene mulandire koyambirira kwa kafukufuku. Ngati muzayezedwe malungo, tizatenga magazi pang'ono kuchokera ma nkono wanu. Tizakupemphani nthawi ina kuti titenge magazi pang'ono pachala panu kuti tizayese malungo kumapeto kwakafukufuku. Tizagwiritsa ntchito magaziwa kuti tiyeze azimayi okwanira zikwi zisanu oyambirira kuti tiyeze ngati ali ndi magazi okwanira. Tikakuyezani ndipo ngati mupezeke kuti mulibe magazi okwanira tizakupatsani mankhwala oyenera panthawiyo.

Mukabwera kuchipatalako ndizakupatsani ndalama zoyendera kubwera kuchipatala ndi kubwerera kunyumba ndiponso ndalama zazakudya ngati tizakusungeni ku chipatala kuno

nthawi yokudya masana. Muzandalira chithandizo chonse chimene mzimayi oyembekezera amayenera kulandira kuchipatala.

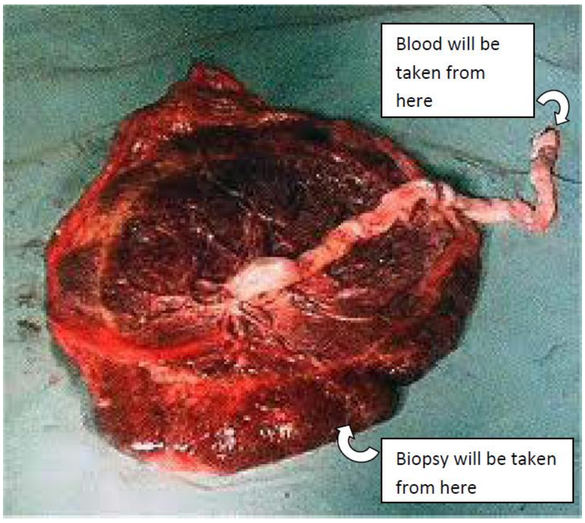

Tikufuna kuti muzaberekere kuchipatala kuno kuti tizaone kuti inu ndi mwana wanu muli bwanji. Mwana wanu akabadwa tizayeza malungo mu msengwa. Tizatenga magazi kuchokera pamkono panu kuti tiyeze ngati muli ndi malungo, ndikuyezanso kuti tione kuti chitetezo chanu ndichokwanira bwanji ku malungo, ndiponso tizayeza kuti tione kuti muli ndi magazi okwanira bwanji mthupi. Tizamuyezanso mwana wanu kuti akulemera bwanji ndiponso kuti tione kuti mthupi mwake muli bwanji. Ngati mukufuna kukaberekera mwana kunyumba, tikupemphani kuti muzatiuze msanga mwanayo akabadwa kuti tizakuyendereni kunyumba kuti tizatenge mfundo zina zokhuzana ndi kafukufukuyi.

Tifuna tizakuoneninso mwana akakwanitsa masiku asanu ndi awiri kenakonso akazakwanitsa milungu isanu nid umodzi kuti tione kuti inu ndi mwana wanu mukupeza bwanji. Simazafunika kubwera kambirimbiri chifukwa tifuna tizakuoneni masiku amene mumabwera kale kusikelo kuti mwana kuti azalandire akatemera.

Ngati mutenge nawo mbali mukafukufuku, tifuna kuti musatenge mankhwala ena ali onse amene simunapatsidwe kuchipatala kuno. Ngati simukupeza bwino mthupi nthawi yomwe muli mukafukufuku, mukhoza kubwera kuchipatala. Muzaonedwa ndi ogwira ntchito pachipatala pano osalipilira kalikonse ndipo muzapatsiwa chithandizo choyenera.

Mfundo zonse zomwe titenge zainu zizagwiritsidwa ntchito ndi ofufuza kuno ku Malawi ndi kunja. Zonse zizasungidwa mwachinsinsi. Maina anu sadzalembedwa pazotsatira zakafukufukuyi.

Nanga ngati sindikufuna kutenga nawo mbali mukafukufuyi?

Ngati simufuna kutenga nawo mbali mukafukufukuyi, mulandira chithandizo chomwe mumayenereka kulandira nthawi zonse mukakhala oyembekezera. Izi ndi matabuleti atatu a SP amene muzapatsidwe mukamumva mwana kusuntha ndi nthawi inanso mudakali oyembekezera. Mukhoza kusintha maganizo ndikusiya kutenga nawo mbali mukafukufuku nthawi iliyonse, ndipo simukusowa kulongosola chifukwa chomwe simukufunira kutenga nawo mbali mukafukufukuyi ngati musakufuna.

Kodi pali zovuta zanji zotenga nawo mbali mukafukufukuyi?

DHA-PQ ndimankhwala amene amagwira bwino ntchito kugonjetsa malungo. Anayezedwapo kale muazimayi oyembekezera kuchokera milungu khumi ndi itatu ndipo anapeza kuti sabweretsa zovuta pa mwana kapena azimayi. Komabe, ndimankhwala atsopano ndiye sitingathe kutsimikiza kuti ngati sapangitsa chovuta chilichonse. Ngati muzapezeke kuti mankhwalawa akudwalitsani muli mukafukufukuyi, tidzasiya kukupatsani DHA-PQ. Ana onse azayezedwa bwinobwino ndipo mavuto onse tizawalemba. Kafukufukuyi azasiyidwa pakapezeka kuti DHA-PQ siwabwino kwa ana.

SP wakhala akugwiritsidwa ntchito muazimayi oyembekezera, ndi wabwino ndipo amaperekedwa kwa amayi onse oyembekezera akapita kusikelo. Inunso mupatsidwa SP ngati simutenga nawo mbali mukafukufukuyi.

Ngati mudwale mukamwa mankhwala a DHA-PQ kapena SP, ndipo ngati tidzawone kuti kudwalako kukhoza kukhala chifukwa cha mankhwala mwamwawo, tikhoza kuzakusiyitsani kumwa mankhwalawo (ndipo osakupatsaninso mankhwala amenewo) koma ngati zizachitike, tidzafunabe kuti muzapitirize kutenga nawo mbali mukafukufuku.

Tikatenga magazi, mukhoza kumva kupweteka kapena pamkono pakena pachala pakhoza kutupa. Komanso pali mwayi oti pamalopo pakhoza kulowa matenda, koma izi sizichitika chitika chifukwa timagwiritsira ntchito zida zoyera.

Mukafukufukumu tifuna kuti muzabwere kuchipatala nthawi zochulukirapo kuposa mmene mungabwerere mukakhala kuti simuli mukafukufuku zoti zikhoza kusokoneza ntchito yanu. Ife tizakupatsani ndalama zolipirira ulendo wanu wobwera kuno kuchipatala.

Tikuthokozani potipatsa nthawi yanu. Mungafune kulowa nawo mukafukufukuyi?

Study consent form (Chichewa)

Kalata yovomereza kuyezedwa ngati muli oyenera

Andilongosolera za kafukufukuyi ndipo ndalolera kuti andiyeze ngati ndili oyenera kulowa mukafukufukuyi.

Kuyezedwaku kukhudza kutenga magazi pang'ono pamkono kuti akayezedwa ndipo mwina kuyezedwa kugwiritsa ntchito ultrasound scan

Ngati ndalolela kuyezedwa kuti aona ngati ndili oyenera, sikuti zitanthauza kuti ndalolera kutenga nawo mbali mukafukufukuyi, ndikhoza kusintha maganizo ntahwi ili yonse.

Zolemba zaotenga nawo mbali mukafukufuku: saini kapena chidindo pa mzere oyenera. Ngati munthu sakufuna kupereka chilolezo, kalatayi isasainidwa.

Andilongosolera zili pamwazi	Ndalolera kutenga mbali mukafukufuku	
Dzina la otenga mbali :	Saini ya otenga mbali :	Date: (tsiku/mwezi/chaka)

Zolemba zamboni (zilembedwe ngati munthu otenga mbali satha kuwerenga):
Kalata ili pamwambayi yalongosoleredwa ndipo mzimayi walolera kutenga mbali mafukufuku:

Dzina la mboni	Saini ya mboni	Date: (tsiku/mwezi/chaka)

Dzina lamkulu wakafukufuku:	Saini ya mkulu wakafukufuku:	Date: (tsiku/mwezi/chaka)

Chilolezo cholowela mukafukufuku

Andilongosolera zakafukufukuyi ndipo ndalolera kutenga nawo mbali mukafukufukuyi.

- Andiuza zonse zovuta ndi phindu lotenga nawo mbali mukafukufukuyi
- Andiuza kuti zili kwaine kusankha kutenga nawo mbali mukafukufukuyi ndipo ndikhoza kusiya nthawi ina iliyonse popanda chovuta chilichonse kwaine ndi mwana wanga
- Ndalolera kuti anthu ogwira ntchito mukafukufukuyi akhoza kuzandiyendera kunyumba kwanga ngati ndilephera kubwera kuchipatala.

Zolemba zaotenga nawo mbali mukafukufuku: saini kapena chidindo pa mzere oyenera. Ngati munthu sakufuna kupereka chilolezo, kalatayi isasainidwa.

Andilongosolera zili pamwazi	Ndalolera kutenga mbali mukafukufuku	
Dzina la otenga mbali :	Saini ya otenga mbali :	Date: (tsiku/mwezi/chaka)

Zolemba zamboni (zilembedwe ngati munthu otenga mbali satha kuwerenga):
Kalata ili pamwambayi yalongosoleredwa ndipo mzimayi walolera kutenga mbali mafukufuku:

Dzina la mboni	Saini ya mboni	Date: (tsiku/mwezi/chaka)

Dzina lamkulu wakafukufuku:	Saini ya mkulu wakafukufuku:	Date: (tsiku/mwezi/chaka)

Anthu owapeza

Ngati muzakhale ndi mafunso ngati mwakhudzidwa ndikutenga mbali mukafukufukuyi, mukhoza kulankhula ndi mmodzi wa anthu amene akugwira ntchito mukafukufukuyi. Ngati muli ndimafunso okhuza kafukufukuyi, kapena ngati mukuona ngati mwana wanu wapweteka, muwapeze a Dr Linda Kalilani-Phiri ku College of Medicine, Private Bag 360, Balntyre 3 kapena pa phoni nambala iyi 01919776. Ngati muli ndi mafunso okhuza zaufulu wanu ngati munthu otenga nawo mbali mukafukufukuyi, kapena ngati mukufuna kulankhula zokhuza kafukufukuyi ndi munthu amene sakutenga nawo mbali mukafukufukuyi mukhoza kulankhul andi Prof Mfutso-Bengo ku College of Medicine, Private Bag 360, Blantyre 3 kapena pa phone nambala iyi 01877291.

Kalata yachilolezo yowonjezera – Kusunga magazi

Tikufuna kuti tisungeko maganize ena amene tatenga kwainu kuti mwina pazafunika kuyeza zinthu zina kafukufukuyi akazatha. Sitisunga magaziwa ngati inuyo simutiloleza.

Ngati mutilolere kuti tisunge magazi anthu, mukhoza kusintha maganizo anthu ndiponso kutiuza mpakana patatha mwezi umodzi chimalizireni kafukufuku. Ngati mufune kuti tisasunge magazi anu, mukhoza kuwapeza a Dr Linda Kalilani pa phone number iyi 01919776.

Pakatha mwezi umodzi kafukufuku atatha, tizachotsa dzina lanu pazosungazi ndiye sitizatha kuchotsa zinthu zanu ngati musinthe maganizo. Ndiponso sitidzatha kukuuzani zosatirazi zazoyeza patsogolo.

Mukhoza kutenga nawo mbali mukafukufuku ngakhale mutakhala kuti simukufuna kuti tisunge magazi anu.

Chonde sankhani malo amodzi pansipa kusonyeza ngati mukufuna kuti tisunge magazi anu kapena ayi.

- ☐ AYI, musasunge magazi anga.
- ☐ EYA, mukhoza kusunga magazi anga koma moti sadziwika kuti ndianga ndipo

Zolemba zaotenga nawo mbali mukafukufuku: saini kapena chidindo pa mzere oyenera. Ngati munthu sakufuna kupereka chilolezo, kalatayi isasainidwa.

Andilongosolera zili pamwazi	Ndalolera kutenga mbali mukafukufuku	
Dzina la otenga mbali :	Saini ya otenga mbali :	Date: (tsiku/mwezi/chaka)

Zolemba zambonni (zilembedwe ngait munthu otenga mbali satha kuwerenga):
Kalata ili pamwambayi yalongosoleredwa ndipo mzimayi walolera kutenga mbali mafukufuku:

Dzina la mboni	Saini ya mboni	Date: (tsiku/mwezi/chaka)

Dzina lamkulu wakafukufuku:	Saini ya mkulu wakafukufuku:	Date: (tsiku/mwezi/chaka)

Appendix 3: Participant information sheet economic study (exit survey among pregnant women at the antenatal clinic) and consent form

PATIENT INFORMATION SHEET ECONOMIC STUDY

The University Of Malawi College Of Medicine and the London School of Hygiene and Tropical Medicine are jointly doing a research study. We invite you to take part in this study. Before you decide whether to take part, it is important for you to understand why the study is being done and what it will involve. Please take the time to read the following information carefully. Ask us if there is anything that is not clear and if you would like more information.

What is the purpose of the study?

Malaria is a common cause of anaemia (lack of blood) in pregnancy and can cause the baby to be born small and weak. Sometimes you can have malaria without feeling sick. It is, therefore, fundamental to prevent malaria during pregnancy. The current strategy to prevent malaria during malaria consists of three tablets of sulphadoxine-pyrimethamine (SP or Fansidar) given three or four times during pregnancy when you visit the clinic after you first feel the baby move. This strategy is called IPTp-SP, which stands for Intermittent Preventive Treatment in pregnancy with Sulphadoxine-Pyrimethamine. Malaria parasites are now becoming resistant to SP, meaning that the drug may not work as well as it used to. For this reason, the University Of Malawi College Of Medicine and the Liverpool School of Tropical Medicine are currently carrying out a clinical study to evaluate a new strategy for control of malaria in pregnancy called ISTp. ISTp stands for Intermittent Screening and Treatment in pregnancy.

Although it can be more efficacious to avoid malaria during pregnancy, a new preventative strategy may imply additional costs for both the health system and the women. Alongside the ISTp clinical study and in order to help choose among strategies to prevent malaria in pregnancy, we want to inform policy makers about the costs pregnant women incur for its treatment and prevention.

Why have I been chosen?

We need to interview pregnant women when they leave the antenatal clinic (ANC) after a visit. To make comparisons, we need to enrol both women who experienced at least one episode of malaria and women who did not experienced any episode of malaria during their current pregnancy.

What will happen if I take part?

You will be administered with a questionnaire and will be asked questions about how many times you have attended the ANC during your current pregnancy and about health tests, care and medical attention you may have received. You will be asked about malaria prevention tools you may have be given at the ANC or that you may have bought or received somewhere else. You will be asked about health related costs incurred during your current pregnancy. Finally, we will ask you information about your family, your occupation, your sources of income, about your house and the assets you and your family own.

What if I don’t want to be in the study?

If you decide not to participate you will not suffer any repercussion as you and your baby will receive all the regular health care that pregnant women and newborns attending the clinic usually receive. If you decide to participate you can also change your mind and withdraw from the study at any time, and you do not have to give a reason if you do not want to.

What are the possible disadvantages and risks of taking part?

This study does not imply either taking biological samples, such as blood, out of your body or administering drugs. Therefore, there is no risk at all for your health or for the health of your baby. This study implies asking personal information such as your age, the composition of your family, your income, the expenses you have incurred and about the assets you and your family own. Participation in the study is completely anonymous: the questionnaire does not include any information, such as name or address, that may help identifying the participants.

Thank you very much for your time. Would you like to participate in the study and reply to questions included in the questionnaire?

Study consent form (English)

CONSENT FOR INCLUSION IN THE STUDY

Participating in the study will involve only replying to questions included in a questionnaire.

Even if you agree to be reply you can change your mind at any time and stop replying questions.

The above study has been explained to me and I agree to reply questions included in the questionnaire.

Participant's statement: sign or thumbprint on the appropriate line. If the participant does not wish to consent, form should not be signed.

The above has been explained to me	I agree to be part of this study	
Participant's name:	Participant 's signature:	Date: (day/month/year)

Witness' statement (optional, except for illiterate participants):

The above consent was explained and the woman agreed to join the study:

Witness' name	Witness' signature	Date: (day/month/year)

Investigator's name:	Investigator 's signature:	Date: (day/month/year)

Contacts

If you later have questions or concerns about your participation in this study, you may speak with one of our staff. If you have any questions about the study, or you feel your child has been harmed, please contact Dr. Victor Mwapasa at the College of Medicine, Private Bag 360, Blantyre 3 or at phone number 01919776.

If you have any questions about your rights as a study participant, or if you want to talk about the study with someone who is not part of this research project, please contact ~~Prof Mfutso-Bengo at the College of Medicine, Private Bag 360, Blantyre 3 or phone number~~

~~01877291~~the National Health Sciences Research Committee, Ministry of Health, P.O.Box 30377, Lilongwe 3 or phone number 01 789 400 .
