# Supplementary material for: Scheduled Intermittent Screening with Rapid Diagnostic Tests and Treatment with Dihydroartemisinin-Piperaquine versus Intermittent Preventive Therapy with Sulfadoxine-Pyrimethamine for Malaria in Pregnancy in Malawi: An Open-Label Randomized Controlled Trial
Source: PLoS Med. 2016 Sep 13;13(9):e1002124. doi: 10.1371/journal.pmed.1002124 (PMC5021271; doi:10.1371/journal.pmed.1002124)
Supplement: S4 Text — (PDF) [file pmed.1002124.s021.pdf]

BioMed Central
The Open Access Publisher

ISRCTN registry

Search Advanced Search

View all studies | Why register? | Register your study | Login | Sign up

ISRCTN69800930 DOI 10.1186/ISRCTN69800930

Scheduled screening versus preventive treatment for the control of malaria in pregnancy in Malawi: a randomized controlled trial

CrossMark

Condition category
Infections and Infestations

Prospective/Retrospective
Prospectively registered

Date applied
14/03/2011

Overall trial status
Completed

Date assigned
07/04/2011

Recruitment status
No longer recruiting

Last edited
01/08/2011

Plain English Summary

Not provided at time of registration

Trial website

Contact information

Type

Scientific

Primary contact

Prof Feiko ter Kuile

ORCID ID

Contact details

Liverpool School of Tropical Medicine
Pembroke Place
Liverpool
L3 5QA
United Kingdom

Additional identifiers

EudraCT number

ClinicalTrials.gov number

Protocol/serial number

Prot 10.74 LSTM; P.07/10/955 (COMREC)

Study information

Scientific title

Scheduled intermittent screening and treatment in pregnancy (ISTp) versus intermittent preventive treatment with sulphadoxine-pyrimethamine (IPTp-SP) in women protected by insecticide treated nets (ITNs) for the control of malaria in pregnancy in Malawi: a randomized controlled trial

Acronym

ISTp-Malawi

Study hypothesis

Scheduled intermittent screening with malaria rapid diagnostic tests (RDTs) and treatment of RDT-positive women with dihydroartemisinin-piperaquine (ISTp-DP) is more effective in prevention malaria associated adverse outcomes in pregnancy than the current strategy of intermittent preventive treatment with sulphadoxine-pyrimethamine (IPTp-SP) in the second and third trimesters among HIV-negative women protected by insecticide-treated bed nets.

Ethics approval

Liverpool School of Tropical Medicine (LSTM) Research Ethics Committee: 28/02/2011; P10.74
College of Medicine research Ethics Committee (COMREC): 26 Nov 2010; P.07/10/955

Study design

Open-label two-arm multicentre randomised controlled superiority trial

Primary study design

Interventional

Secondary study design

Randomised controlled trial

Trial setting

Hospitals

Trial type

Prevention

Patient information sheet

Not available in web format, please use the contact details below to request a patient information sheet

Condition

Control of malaria in pregnancy

Intervention

Participants will receive one of the following two interventions during the second and third trimesters of pregnancy which will be provided at each of three scheduled visits between four and six weeks apart:
1. IPTp-SP Group: Treatment with a three tablets of sulphadoxine-pyrimethamine, each containing sulphadoxine (500 mg) and pyrimethamine (25 mg). This is the standard and only drug for IPTp in Africa.
2. ISTp-DP Group: Screening for malaria using a combined HRP-2/ pLDH (P. falciparum/ pan-malaria) rapid diagnostic test (First Response® Malaria pLDH/HRP2 Combo Test, target antigen pLDH (pan); HRP2; Premier Medical Corporation Ltd, USA), and treatment if RDT-positive with dihydroartemisinin-piperaquine (Sigma Tau). Each tablet will contain 40 mg dihydroartemisinin and 320 mg piperaquine.
3. Treatment will be given for three days, with the daily number of tablets depending on the weight of the woman to the nearest half tablet; dosage being 2 mg/kg/day of dihydroartemisinin and 16 mg/kg/day piperaquine

Intervention type
Other

Phase
Not Specified

Drug names

Primary outcome measures
1. In women in their first or second pregnancy: composite endpoint of adverse birth outcomes, defined as any of:
1.1. Small for gestational age defined as a binary outcome of <10th percentile of fetal weight for attained gestational age
1.2. Preterm birth (spontaneous birth before 37 weeks gestation)
1.3. Low-birth-weight (birth weight under 2,500 grams)
2. In women in their third to fifth pregnancies: Malaria infection at term and delivery will be the primary endpoint, defined as evidence of current or recent infection assessed at delivery by placental histopathology (active or past infection) or rapid diagnositc tests (RDT) (pLDH or HRP2 positive, any species) or PCR positive (any species)

Secondary outcome measures
1. Placental malaria (any species)
1.1. Past infection detected by histopathology
1.2. Active infection detected by:
1.2.1. Histopathology
1.2.2. Microscopy
1.2.3. Rapid diagnostic test
1.2.4. Polymerase chain reaction (PCR)
2. Maternal malaria infection (peripheral blood) at delivery, detected by:
2.1. Microscopy
2.2. RDT
2.3. PCR
3. Peripheral malaria infection during pregnancy detected by:
3.1. Microscopy
3.2. PCR
4. Birth weight
4.1. Mean birth weight (grams)
4.2. Low birth weight (<2,500 grams)
5. Gestational age
5.1. Mean gestational age at birth (grams)
5.2. Pre-term birth (<37 weeks)
6. Small for gestational age
7. Maternal haemoglobin and anaemia at delivery:
7.1. Mean maternal haemoglobin (g/dL)
7.2. Anaemia (Hb ≤ 11 g/dL)
7.3. Moderate to severe anaemia (Hb ≤ 8g/dL)
8. Maternal haemoglobin and anaemia during third trimester:
8.1. Mean maternal haemoglobin (g/dL)
8.2. Anaemia (Hb ≤ 11 g/dL)
8.3. Moderate to severe anaemia (Hb ≤ 8g/dL)
9. Miscarriage (loss of foetus before 28 weeks gestation)
10. Stillbirth (birth at 28 weeks or later showing no signs of life)
11. Composite endpoint of the primary endpoint plus fetal loss (miscarriage or stillbirths)
12. Infant death
13. Perinatal death (stillbirth or death within 7 days of birth)
14. Neonatal death (death within 28 days of birth)
15. Malaria infection of the newborn, detected by analysis of umbilical cord blood with:
15.1. RDT
15.2. Microscopy
15.3. PCR
16. Foetal haemoglobin and anaemia by sampling of umbilical cord blood at birth:

[illegible]
16.1. Mean foetal haemoglobin (g/dL)
16.2. Foetal anaemia (Hb ≤ 12.5 g/dL)
16.3. Moderate to severe foetal anaemia
17. Incidence of documented clinical malaria episodes during the second and third trimesters of pregnancy (history of fever in last 24 hours and documented malaria microscopy or RDT positive)
18. Presence of any evidence of malaria infection at term (last antenatal visit), identified through microscopy or PCR, or at delivery, identified through peripheral and placental RDT, microscopy or PCR, or placental histopathology (active or past infection).
19. Incidence of other illness episodes apparent at scheduled antenatal clinic visits or resulting in unscheduled clinic visits
20. Incidence and prevalence of clinical malaria in infants by seven days and six to eight weeks determined by:
20.1. RDT
20.2. Microscopy
20.3. PCR
21. Prevalence of symptomatic infant anaemia at seven days and six to eight weeks:
21.1. Anaemia
21.2. Moderate to severe anaemia
22. Incidence of other illness episodes in the infants, apparent at scheduled postnatal clinic visits or resulting in unscheduled postnatal clinic visits
23. Safety outcomes:
23.1. Severe cutaneous skin reaction in the mothers within 30 days of drug intake
23.2. Other serious adverse events in the mothers
23.3. Congenital malformations identified by six weeks after birth
23.4. Neonatal jaundice within 24 hours and at seven days
23.5. Laboratory test results outside of normal range
24. Tolerability outcomes:
24.1. Non-serious adverse events in the mothers
24.2. Adherence to study medication
25. Immunology outcomes:
25.1. Concentration of antibodies known to be associated with protection against malaria in pregnancy and in general, including antibodies recognizing variant surface antigens on P. falciparum infected erythrocytes that block parasite adhesion to chondroitin sulphate A.
26. Economic outcomes (sub-study):
26.1. The economics sub study will be conducted alongside the main clinical trial. Health facility and exit surveys will be carried out to estimate the costs of the intervention to the health services and households respectively. To capture costs incurred during the first six to eight weeks after delivery, including the costs of caring for low birth weight babies, questions about use of health services will be integrated into the clinical health assessment at six weeks.
26.2. Costs of the two intervention arms to the health facility and household up to six to eight weeks after delivery. Household data will be collected by questionnaire at the six-week clinic visit; health facility data directly from the health facility
26.3. Cost-effectiveness of ISTp-DP versus IPTp-SP measured in terms of cost per each of the following endpoints averted, which are measured in the main trial:
26.4. Adverse birth outcome (still birth, preterm birth or low birth weight)
26.5. Active and past malaria infection of the placenta (detected by histopathology, microscopy or RDT)
26.6. Maternal anaemia
26.7. Peripheral malaria at delivery
26.8. Neonatal deaths
26.9. Cost per disability adjusted life year (DALY) averted will be estimated using the cost data collected and effectiveness data generated by the trial, with necessary adjustments made to the DALY to accommodate outcomes in pregnant women and their newborns
The household and facility data from the cost and cost-effectiveness analysis will be used (alongside data from other studies) to populate a model of the economic burden of malaria in pregnancy.
27. Model the long-term costs and consequences of malaria in pregnancy to the household and health facility in both trial arms
28. Model the costs of scaling up the intervention at regional/national level and investigate affordability in Malawi
29. Acceptability, feasibility, implementability and scale up outcomes
29.1. The overall aim of this sub study is to explore the acceptability, feasibility, implementability and potential for scale-up of ISTp-DP

29.2. Social, cultural and economic determinants of demand, access and use for malaria in pregnancy interventions
29.3. Acceptability of ISTp for provider and user
29.4. Preferences for malaria in pregnancy interventions at the user and provider level
29.5. Factors at facility and district levels which influence the delivery of malaria in pregnancy interventions and in particular the feasibility and implementability of ISTp in the context of other reproductive health interventions (e.g. prevention of mother to child transmission)
29.5. Major barriers to the scale-up and use of interventions to control malaria in pregnancy, specifically of ISTp

Overall trial start date

01/05/2011

Overall trial end date

01/11/2013

Reason abandoned

Eligibility

Participant inclusion criteria

1. Viable singleton pregnancy
2. Gestational age 16 to 28 weeks (inclusive) by LMP (if available) or fundal height
3. No history of IPTp use during this pregnancy
4. Willing to participate and complete the study schedule
5. Has provided written informed consent
6. Resident of study area and intending to stay in the area for the duration of the follow-up
7. Willing to deliver in the labour ward of the study clinic or hospital

Participant type

Patient

Age group

Adult

Gender

Female

Target number of participants

1655 consisting of two strata: 1155 primigravidae, and secundigravidae (G1+2) and 500 multigravidae (G3+)

Participant exclusion criteria

1. HIV positive or unknown HIV status
2. Multiple gestations
3. High risk pregnancy resulting in referral to tertiary delivery facilities according to local guidelines
4. Severe anaemia requiring blood transfusion (Hb ≤ 7.0 g/dL) at enrolment
5. Known allergy or previous adverse reaction to any of the study drugs
6. Unable to give informed consent (for example due to mental disability)
7. Previous inclusion in the same study

Recruitment start date

01/05/2011

Recruitment end date

01/11/2013

Locations

Countries of recruitment
Malawi

Trial participating centre
Liverpool School of Tropical Medicine
Liverpool
L3 5QA
United Kingdom

Sponsor information

Organisation
Liverpool School of Tropical Medicine (UK)

Sponsor details
c/o Sian Roberts
Pembroke Place
Liverpool
L3 5QA
United Kingdom

Sponsor type
University/education

Website
http://www.liv.ac.uk/lstm/

Funders

Funder type
Government

Funder name
European and Developing Countries Clinical Trial Partnership (EDCTP)

Alternative name(s)

Funding Body Type

Funding Body Subtype

Location

Results and Publications

Publication and dissemination plan
Not provided at time of registration

Intention to publish date

Participant level data
Not provided at time of registration

Not provided at time of registration

Results - basic reporting

Publication summary

Publication citations

Additional files

Editorial Notes

About
Governance
Why register?
Search tips
Resources
FAQs
Follow us on Twitter
Publish with BioMed Central
Contact us

© 2016 BioMed Central Ltd unless otherwise stated. Part of Springer Science+Business Media.

Terms & Conditions and Privacy Policy

Springer
